# MitoStores: chaperone‐controlled protein granules store mitochondrial precursors in the cytosol

**DOI:** 10.15252/embj.2022112309

**Published:** 2023-01-27

**Authors:** Lena Krämer, Niko Dalheimer, Markus Räschle, Zuzana Storchová, Jan Pielage, Felix Boos, Johannes M Herrmann

**Affiliations:** ^1^ Cell Biology University of Kaiserslautern Kaiserslautern Germany; ^2^ Molecular Genetics University of Kaiserslautern Kaiserslautern Germany; ^3^ Zoology and Neurobiology University of Kaiserslautern Kaiserslautern Germany; ^4^ Present address: Cellular Biochemistry Max Planck Institute of Biochemistry Martinsried Germany; ^5^ Present address: Department of Genetics Stanford University Stanford CA USA

**Keywords:** chaperones, mitochondria, proteasome, protein aggregates, protein translocation, Organelles, Translation & Protein Quality

## Abstract

Hundreds of nucleus‐encoded mitochondrial precursor proteins are synthesized in the cytosol and imported into mitochondria in a post‐translational manner. However, the early processes associated with mitochondrial protein targeting remain poorly understood. Here, we show that in *Saccharomyces cerevisiae*, the cytosol has the capacity to transiently store mitochondrial matrix‐destined precursors in dedicated deposits that we termed MitoStores. Competitive inhibition of mitochondrial protein import via clogging of import sites greatly enhances the formation of MitoStores, but they also form during physiological cell growth on nonfermentable carbon sources. MitoStores are enriched for a specific subset of nucleus‐encoded mitochondrial proteins, in particular those containing N‐terminal mitochondrial targeting sequences. Our results suggest that MitoStore formation suppresses the toxic potential of aberrantly accumulating mitochondrial precursor proteins and is controlled by the heat shock proteins Hsp42 and Hsp104. Thus, the cytosolic protein quality control system plays an active role during the early stages of mitochondrial protein targeting through the coordinated and localized sequestration of mitochondrial precursor proteins.

## Graphical abstract

Transient storage in newly identified cytosolic granules protects yeast cells from the toxic effects of excess mitochondrial matrix‐destined precursors.

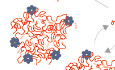

## Introduction

Mitochondria consist of hundreds of different proteins that are synthesized in the cytosol and subsequently imported into mitochondria by translocases in the outer and inner membranes (Chacinska *et al*, 2009). In nerve, muscle, or brown adipose cells, but also in respiring yeast cells, mitochondrial precursors represent a considerable fraction of all nascent protein chains. Only a minor fraction of the nuclear‐encoded mitochondrial proteins is synthesized on the mitochondrial surface (Williams *et al*, 2014). Owing to the post‐translational import mode of most mitochondrial proteins (Wienhues *et al*, 1991), cytosolic precursors explore the cytosol, strain on the cytosolic chaperone system (Deshaies *et al*, 1988; Jores *et al*, 2018), and pose a threat to cellular proteostasis (Wang & Chen, 2015; Wrobel *et al*, 2015; Sorrentino *et al*, 2017; Weidberg & Amon, 2018; Nowicka *et al*, 2021; Schafer *et al*, 2022). Mitochondrial proteins of the outer membrane, the intermembrane space, the inner membrane, and the matrix differ in their import routes and targeting signals; in the following, we refer to the cytosolic native forms of all of these proteins as precursor proteins or precursors (Chacinska *et al*, 2009).

Eukaryotic cells carefully sense the levels of mitochondrial precursor proteins in the cytosol and respond to accumulating mitoprotein levels with adaptations in gene expression, which are summarized with the umbrella term mitoprotein‐induced stress response (Boos *et al*, 2020). In animal cells (in particular those of the nematode *Caenorhabditis elegans*), this is achieved via dedicated transcription factors containing a mitochondrial targeting sequence; under conditions that prevent an unconstrained uptake of these proteins into mitochondria, they enter the nucleus and launch specific transcriptional responses. This unfolded protein response of mitochondria (UPR^mt^) increases the import and folding capacity of mitochondria and results in the expansion of the mitochondrial network in general (Nargund *et al*, 2012; Fiorese *et al*, 2016; Labbadia *et al*, 2017; Aras *et al*, 2020; Shpilka *et al*, 2021; Xin *et al*, 2022).

**Figure 1:**
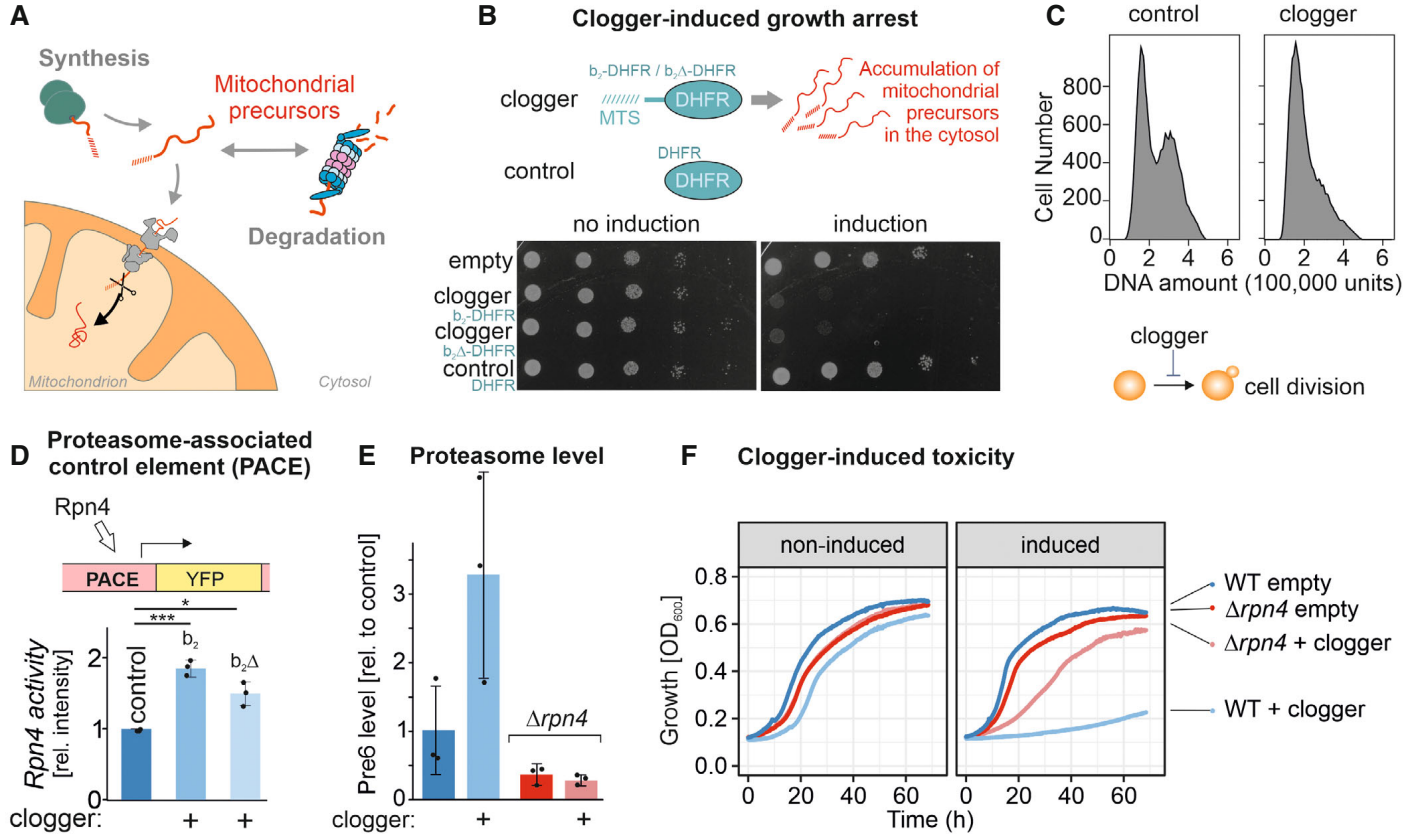
Rpn4‐mediated proteasome induction represents one arm of the mitoprotein‐induced stress response Mitochondrial protein import is under the surveillance of the proteasome.Competitive inhibition of mitochondrial protein import by clogger proteins inhibits cell growth. Yeast cells expressing clogger proteins (cytochrome *b*_2_‐DHFR and cytochrome *b*_2_Δ_19_‐DHFR) or cytosolic DHFR for comparison under the control of the galactose‐inducible promoter were grown to mid‐log phase on lactate medium. Ten‐fold serial dilutions were dropped on lactate (no induction) or lactate with 0.5% galactose medium (induction). MTS, matrix‐targeting signal.After expression of the clogger *b*_2_‐DHFR or the DHFR control, cellular DNA was stained with propidium iodide and DNA content was assessed by flow cytometry. Please note that the first peak shows cells in G1 phase, and the second peak represents cells in the G2 phase.Rpn4‐driven gene induction was measured using a reporter that expressed the yellow fluorescence protein under control of a PACE element. Cells were grown in lactate medium, and clogger expression was induced for 4.5 h with galactose before starting the experiment (Boos *et al*, 2019). Data are displayed as mean ± standard deviations from *n* = 3 independent biological replicates. Significance was assessed using a two‐sided, paired Student's *t*‐test. *P*‐values are indicated as asterisks. *P ≤ 0.05, ***P ≤ 0.001.Levels of the proteasome protein Pre6 were detected upon the expression of cytosolic DHFR or clogger by Western blotting and quantified from three replicates. Cells were grown in lactate medium and clogger expression was induced for 4.5 h with 0.5% galactose before samples were harvested and lysed. Data are displayed as mean ± standard deviations from *n* = 3 independent biological replicates.Cells of the indicated strains were grown to log phase and diluted in lactate (no induction) or lactate with 0.5% galactose (induction) to 0.1 OD_600_. Growth was monitored upon constant agitation at 30°C. Source data are available online for this figure.

In yeast, no specific transcription factors were identified, but the general accumulation of nonimported precursor proteins launches the Unfolded Protein Response activated by mistargeting of proteins (UPRam), which increases the capacity of the cytosolic ubiquitin–proteasome system (UPS) by an upregulation of constituents of the degradation machinery and by stimulating their assembly (Wrobel *et al*, 2015; Boos *et al*, 2019). This upregulation of the proteasome is mediated by the transcription factor Rpn4 (Boos *et al*, 2019). Rpn4 serves as the master regulator of proteasome levels in baker's yeast and is tightly controlled by the proteasome; if the proteasome system is overstrained, Rpn4 accumulates and induces a 2–4 times upregulation of components of the ubiquitin–proteasome system compared with nonstressed conditions (Xie & Varshavsky, 2001; Metzger & Michaelis, 2009). Thereby, nonimported mitochondrial precursor proteins are under tight surveillance of the proteasome in order to prevent an overload of the mitochondrial protein import system (Fig 1A).

Cellular protein homeostasis, called proteostasis, is of central relevance for cellular functionality and organismal health (Labbadia & Morimoto, 2015). Proteostasis relies on the dynamic interplay of two closely cooperating systems, the network of molecular chaperones facilitating protein folding (Hipp *et al*, 2019) and the cellular protein degradation systems, particularly the ubiquitin–proteasome system (UPS; Dikic, 2017; Varshavsky, 2017). Failure of either of these systems results in the accumulation of misfolded proteins, a characteristic hallmark of aging and numerous diseases, including but not restricted to neurodegenerative amyloid disorders such as Alzheimer's or Huntington's disease (Hartl, 2017; Pilla *et al*, 2017).

When chaperones and the proteasome are overstrained, cells use a second line of defense: to suppress their cellular toxicity, misfolded proteins can be spatially sequestrated at dedicated locations. Several types of quality control compartments were previously defined based on their intracellular location and protein composition (Sontag *et al*, 2017). In the baker's yeast *Saccharomyces cerevisiae*, sudden shifts to high temperature, proteasome impairments, or defects in chaperone function lead to a transient accumulation of Q‐bodies (also referred to as CytoQs or stress granules), which, upon prolonged stress conditions, can coalesce into larger JUNQs and INQs (juxtanuclear and intranuclear quality control compartments, respectively) on both sides of the nuclear envelope (Escusa‐Toret *et al*, 2013). Whereas the proteins of Q‐bodies, JUNQs, and INQs are rapidly released and degraded once stress conditions are relieved, insoluble protein deposits (IPODs), for example, those formed upon expression of polyQ‐expanded huntingtin, are highly stable and pose a severe threat to cellular function (Gruber *et al*, 2018; Mogk *et al*, 2018). In addition, different, less defined types of protein aggregates were observed on the surface of mitochondria and of the endoplasmic reticulum (ER; Zhou *et al*, 2014; Andreasson *et al*, 2019). Recent studies suggest that nonimported mitochondrial precursor proteins contribute to the formation of cytosolic aggregates (Nowicka *et al*, 2021; Schlagowski *et al*, 2021; Xiao *et al*, 2021).

In this study, we elucidated the consequences of competitive inhibition of mitochondrial protein import in an Rpn4‐deficient mutant in which the proteasome cannot be upregulated. To our surprise, we observed that these mutants escape the mitoprotein‐induced growth arrest and tolerate the accumulation of mitochondrial precursors better than wild‐type cells. Apparently, these strains employ an alternative mechanism to cope with nonimported protein precursors: they form cytosolic granules that we named MitoStores. We show that these structures contain a specific subset of mitochondrial precursor proteins and store them transiently until the stress is resolved. Thus, in contrast to previous assumptions (Chen & Douglas, 1987; Neupert & Herrmann, 2007), the cytosol has the capacity to store mitochondrial precursor proteins in dedicated storage granules that are controlled by the cytosolic chaperone system.

## Results

### Cells lacking Rpn4 can bypass the clogger‐induced growth arrest

**Figure EV1:**
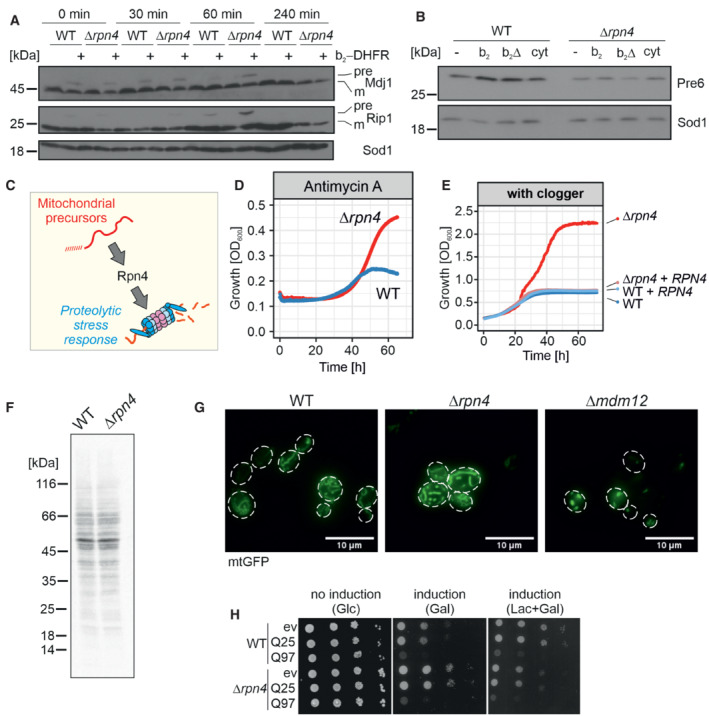
Rpn4‐dependent proteasome induction After 4.5 h of clogger expression in wild‐type and *Δrpn4* cells, the medium was exchanged for a noninducing lactate medium. Precursor (pre) and mature (m) forms of the mitochondrial proteins Mdj1 and Rip1 were visualized by Western blotting. Sod1 was used as a loading control.After 4.5 h expression of clogger and control, protein levels of the proteasome subunit Pre6 were visualized by Western Blotting. Please note, that in *Δrpn4* cells, clogger expression does not lead to Pre6 induction. Sod1 served as a loading control.Schematic representation of Rpn4‐dependent proteasome induction after mitoprotein‐induced stress.Cells of the indicated strains were grown to log phase and diluted in lactate medium to 0.1 OD_600_. After the addition of 100 μg/ml antimycin A, growth was monitored upon constant agitation. Since antimycin A loses its activity over time, cells start growing once the membrane potential reaches a level that allows mitochondrial biogenesis. *Δrpn4* cells escaped the antimycin A‐mediated growth inhibition more efficiently than wild‐type cells.Clogger‐expressing wild‐type and *Δrpn4* cells harboring empty or Rpn4‐expression plasmids were grown over time in lactate plus 0.5% galactose medium.Wild‐type and *Δrpn4* cells were grown in lactate medium to log phase. 2 μl of 22 μCi of ^35^S‐methionine was added to the cell suspension. After 5 min, cells were harvested, lysed, and subjected to SDS gel electrophoresis and autoradiography.The indicated strains were transformed with plasmids expressing mitochondria‐targeted GFP (mtGFP). The mitochondrial network was visualized by microscopy upon growth on galactose. Cells lacking Mdm12 were used as a control for a strain showing defective mitochondrial network formation (Dimmer *et al*, 2002). Scale bars, 10 μm.Yeast cells expressing a fusion protein of the N‐terminal region of human huntingtin with different lengths of glutamine residues (Q25‐GFP and Q97‐GFP, respectively; Schlagowski *et al*, 2021) and GFP under control of the galactose‐inducible promoter, or a control for comparison were grown to mid‐log phase on noninducing glucose medium. Ten‐fold serial dilutions were dropped on glucose (no induction), galactose (induction), or lactate with 0.5% galactose medium (induction). Source data are available online for this figure.

Overexpression of slowly imported precursor proteins, so‐called cloggers, causes growth arrest of yeast cells by activating a block of cell division (Fig 1B and C). The accumulation of mitochondrial precursor proteins in the yeast cytosol (Fig EV1A) induces an Rpn4‐mediated induction of genes that are under the control of a proteasome‐associated control element, PACE (Boos *et al*, 2019; Zöller *et al*, 2020). Accordingly, expression of clogger proteins destined to the mitochondrial matrix or the IMS (b_2_Δ‐DHFR or b_2_‐DHFR, respectively; here driven by a galactose‐inducible promoter) increase the level of the proteasome system in an Rpn4‐dependent fashion (Figs 1D and E, and EV1B and C).

However, to our surprise, we observed that Rpn4 deletion mutants showed a markedly increased clogger resistance. Clogger expression in *Δrpn4* cells was better tolerated, and these cells grew efficiently when the mitochondrial import system was challenged by clogger expression (Fig 1F) or after inhibition of the respiratory chain (Fig EV1D). This resistance was abolished upon re‐expression of Rpn4 in the *Δrpn4* mutant (Fig EV1E). The absence of Rpn4 did neither affect protein synthesis rates in these cells (Fig EV1F) nor the morphology of the mitochondrial network (Fig EV1G). In contrast to the protective effects against mitochondrial dysfunction, deletion of Rpn4 did not increase the resistance to the expression of aggregation‐prone model proteins in the cytosol such as the huntingtin‐derived polyglutamine protein Q97‐GFP, luciferase^R188Q,R261Q^, or Ubc9^Y68L^ (Figs EV1H and EV3A and B). Hence, ablation of the master regulator of the ubiquitin–proteasome system renders cells resistant to mitochondrial stress but not against proteotoxic stress in general.

### *Δrpn4* cells respond to clogger induction by upregulation of Hsp42 and Hsp104

**Figure 2:**
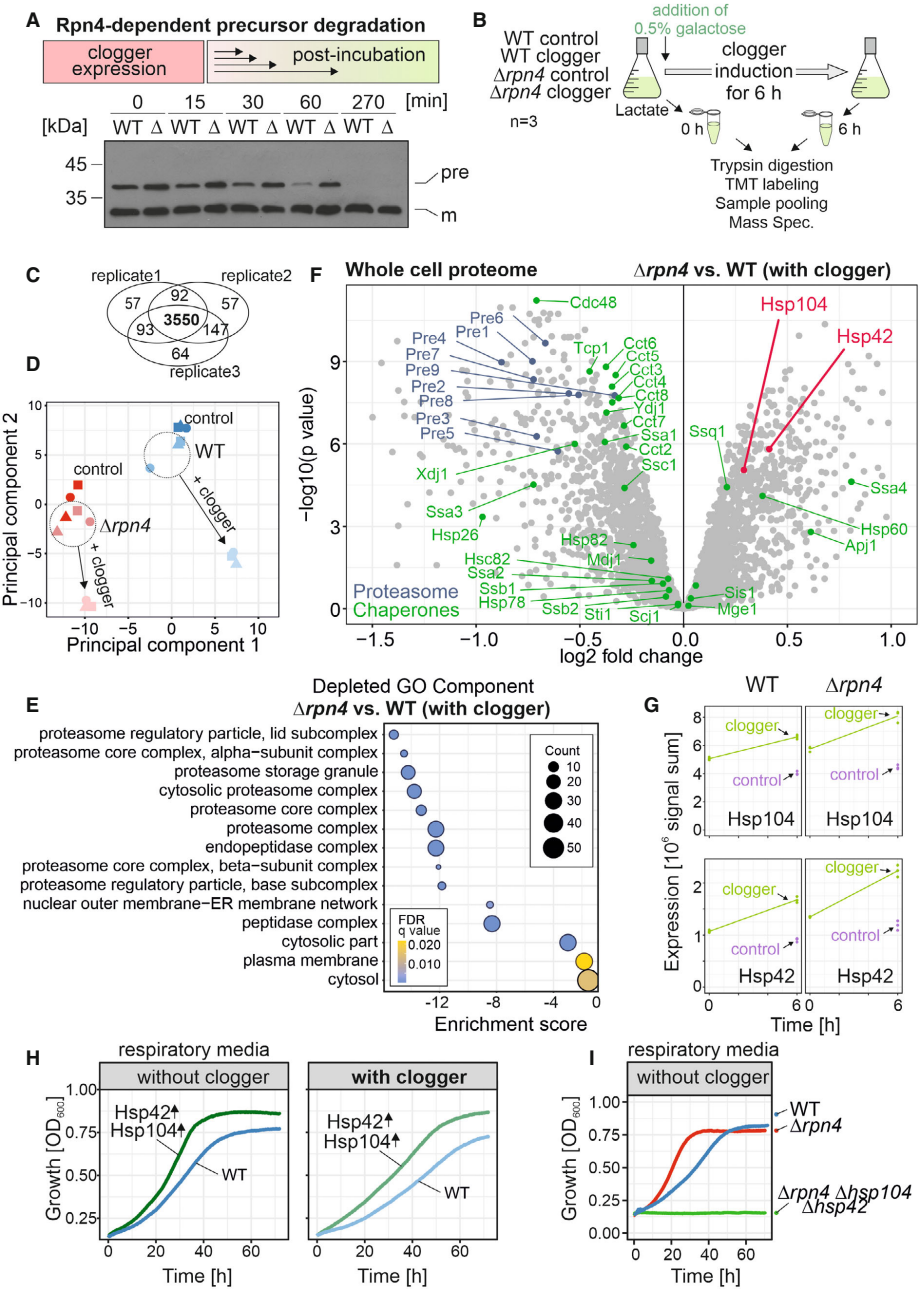
Competitive inhibition of mitochondrial protein import in *Δrpn4* cells remodels the cytosolic chaperone system The clogger was expressed in wild‐type (WT) and *Δrpn4* (Δ) cells for 4.5 h with medium containing 0.5% galactose. Medium was exchanged for a noninducing lactate medium. Precursor (pre) and mature (m) forms of the *b*_2_Δ‐DHFR clogger were visualized by Western blotting using a DHFR‐specific antibody.The clogger was induced for 6 h with 0.5% galactose before cellular proteomes were measured by mass spectrometry. See Materials and Methods for further details.3,550 proteins were measured in all samples of the three replicates.Principal component analysis. Presence of Rpn4 and clogger expression caused specific changes in the proteome. The different shapes of the data points indicate the three different biological replicates.GO Term Analysis was done using the GOrilla tool (http://cbl‐gorilla.cs.technion.ac.il/; Eden *et al*, 2009). The absence of Rpn4 caused a broad reduction in the components of the proteasome‐ubiquitin system. The number of proteins in the indicated GO components is stated as “Counts.”Comparison of the proteome of wild‐type and *Δrpn4* cells after clogger expression for 6 h. Positions of proteins of the proteasome and chaperone systems are indicated in blue and green, respectively. Please note that Hsp104 and Hsp42 (indicated in magenta) show a higher abundance in *Δrpn4* cells. See Dataset EV1 for details.Signals of Hsp104 and Hsp42 before and after clogger induction in wild‐type and *Δrpn4* cells. The cytosolic soluble DHFR protein was expressed for control. The strains were grown in lactate medium and clogger expression was induced with 0.5% galactose for 6 h.Wild‐type carrying plasmids for the GAL‐induced expression of the clogger and for the constitutive expression (from *TPI* promoter) of Hsp42 and Hsp104 were grown on lactate medium to mid‐log phase and used to inoculate cultures with synthetic lactate (no clogger expression) or lactate plus 0.5% galactose (with clogger). Cell growth was measured over time.The respective strains were grown in lactate medium. Growth was monitored upon constant agitation at 30°C. Source data are available online for this figure.

How do *Δrpn4* cells escape the mitoprotein‐induced toxicity? First, we tested whether Rpn4 influences the expression of the clogger protein. To this end, we expressed the clogger for 4.5 h and then switched cells back from galactose to lactate to monitor clogger stability. As expected, the reduced proteasome capacity in *Δrpn4* cells impaired the proteolytic removal of the clogger protein from mitochondria (Fig 2A) and thereby even further increased the accumulation of mitochondrial precursor proteins in the cytosol (Fig EV1A). Thus, yeast cells normally respond to mitoprotein‐induced stress by an upregulation of the proteasome system; however, if this response is prevented, a second line of defense exists, that even more efficiently alleviates the toxicity arising from accumulating mitochondrial precursor proteins in the cytosol.

**Figure EV2:**
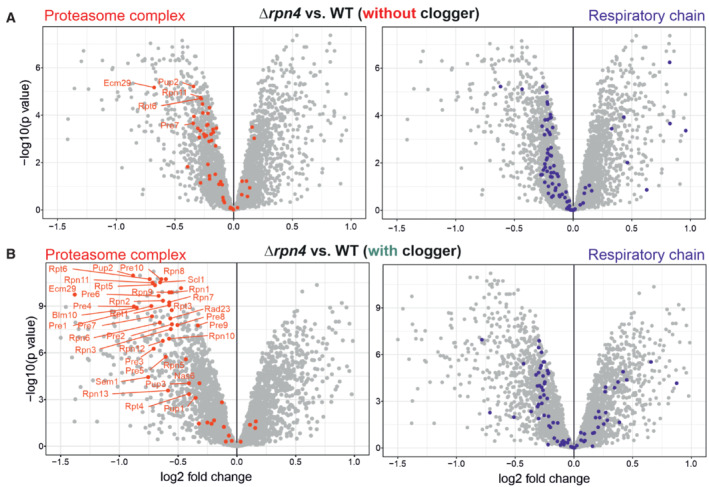
Rpn4 deletion leads to a reduction in proteasomal and respiratory proteins A, B
Comparison of the proteome of wild‐type and *Δrpn4* cells before and after clogger expression for 6 h. Positions of proteins of the proteasome and the respiratory chain systems are indicated in red and purple, respectively (Morgenstern *et al*, 2017; Boos *et al*, 2019). Please note especially the proteasomal proteins show a lower abundance in *Δrpn4* cells.

To identify the underlying molecular mechanism, we compared the cellular proteomes of wild‐type and *Δrpn4* cells before and upon clogger induction (Fig 2B) using isobaric mass tag TMT labeling and quantitative mass spectrometry (Dayon *et al*, 2008). More than 3,800 proteins were quantified in at least two out of three replicates across all samples (Fig 2C, Dataset EV1). This showed that the proteomes of wild‐type and *Δrpn4* cells differed considerably already before clogger expression (Fig 2D) and that the loss of Rpn4 leads not only to reduced levels of proteasome proteins but also to lower amounts of proteins of the mitochondrial respiratory chain (Figs 2E and EV2A and B). Our observations confirmed that Rpn4 is crucial for the induction of the proteasome system, and clogger induction did not bypass the relevance of Rpn4 for proteasome upregulation (Fig EV2B). This again suggests that *Δrpn4* cells employ a proteasome‐independent mechanism to cope with cytosolic‐located precursor proteins.

While the levels of most chaperones were unaffected or even reduced in *Δrpn4* cells, the disaggregase Hsp104 and the small heat shock protein Hsp42 were considerably upregulated (Fig 2F and G), potentially as a proteasome‐independent protection against accumulating mitochondrial precursor proteins. Indeed, constitutive overexpression of Hsp42 and Hsp104 in wild‐type cells improved cell growth under nonfermentative growth conditions and provided additional resistance to clogger expression (Fig 2H). On the contrary, the deletion of Hsp42 and Hsp104 in *Δrpn4* cells abolished their ability to grow on nonfermentable carbon sources (Fig 2I). Apparently, if cells have to use respiration, they either need to upregulate the proteasome or to induce the cytosolic chaperones Hsp42 and Hsp104.

### Hsp104 binds to cytosolic granules containing mitochondrial precursor proteins

**Figure EV3:**
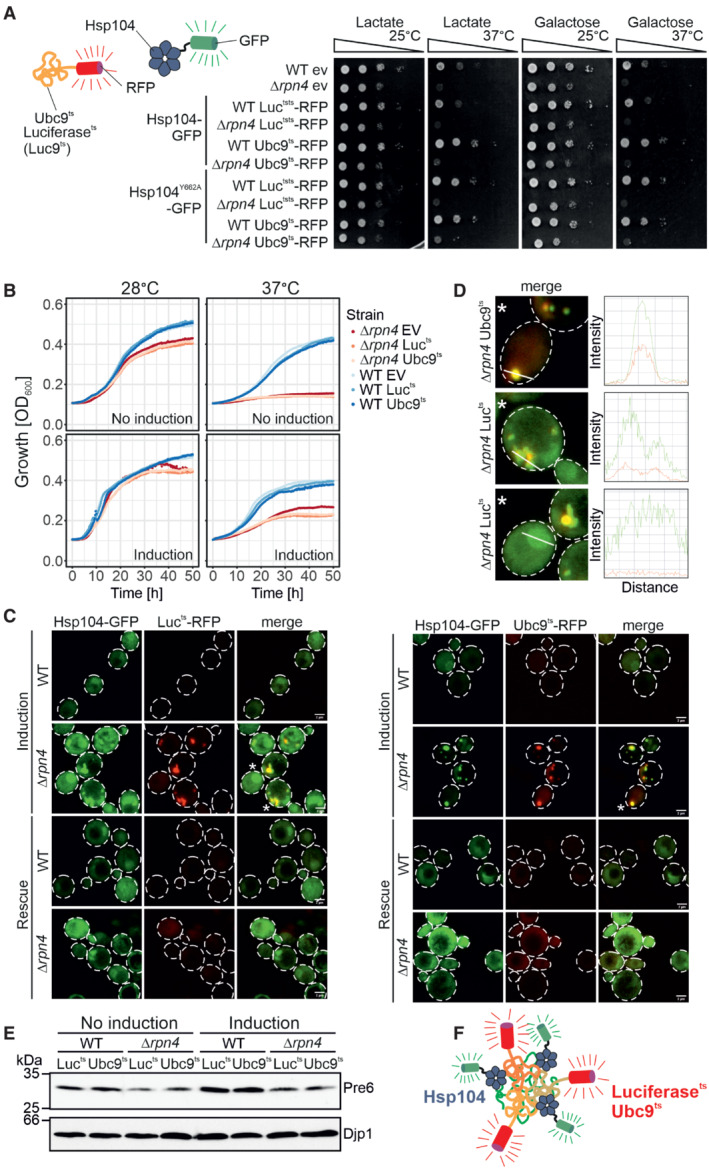
Protein aggregation in the yeast cytosol can be efficiently measured by visualization of the distribution of Hsp104‐GFP Yeast cells expressing the temperature‐sensitive, aggregation‐prone proteins luciferase^ts^‐RFP (Luc^ts^‐RFP) and Ubc9^ts^‐RFP under galactose‐inducible promoters together with constitutively expressed Hsp104‐GFP or Hsp104^Y662A^‐GFP were grown to mid‐log phase on lactate medium. Ten‐fold serial dilutions were dropped on lactate (no induction) or galactose medium (induction) and incubated at 25°C or 37°C as indicated.Cells of the indicated strains were grown to log phase and diluted in lactate (no induction) or galactose (induction) to 0.1 OD. Growth was monitored upon constant agitation at 28°C and 37°C, respectively.After 2 h of Luc^ts^‐RFP or Ubc9^ts^‐RFP expression together with constitutive Hsp104‐GFP expression at 37°C (induction), cells were incubated for 4 h in the absence of galactose at 30°C (rescue). Scale bars, 2 μm.Fluorescence intensity profiles of cells marked with an asterisk in (C). The measured area is indicated with a white line.After 4 h growth on lactate medium (no induction) or galactose medium (induction), the protein level of the proteasomal protein Pre6 was visualized by Western Blotting. Djp1 was used as a loading control.Schematic representation of the interaction of aggregation‐prone proteins with Hsp104. Source data are available online for this figure.

Hsp104 is a hexameric chaperone in the yeast cytosol, which binds to misfolded or aggregated proteins and disentangles them in an ATP‐dependent manner (Sanchez & Lindquist, 1990; Gates *et al*, 2017). Thereby, it associates with different types of cellular aggregates (Sontag *et al*, 2017), including transiently forming benign condensates of dynamic nature (Yoo *et al*, 2022) but also insoluble protein deposits (IPODs) that pose a threat to cellular functionality (Gruber *et al*, 2018; Mogk *et al*, 2018). Consistent with previous studies (Kaganovich *et al*, 2008; Ruan *et al*, 2017), we found that fusion proteins of Hsp104 or an Hsp104^Y662A^ trapping mutant with GFP are well tolerated by yeast cells and can serve as powerful tools to visualize condensates formed by the aggregation‐prone model proteins luciferase^R188Q,R261Q^ and Ubc9^Y68L^ (Fig EV3A–F).

**Figure 3:**
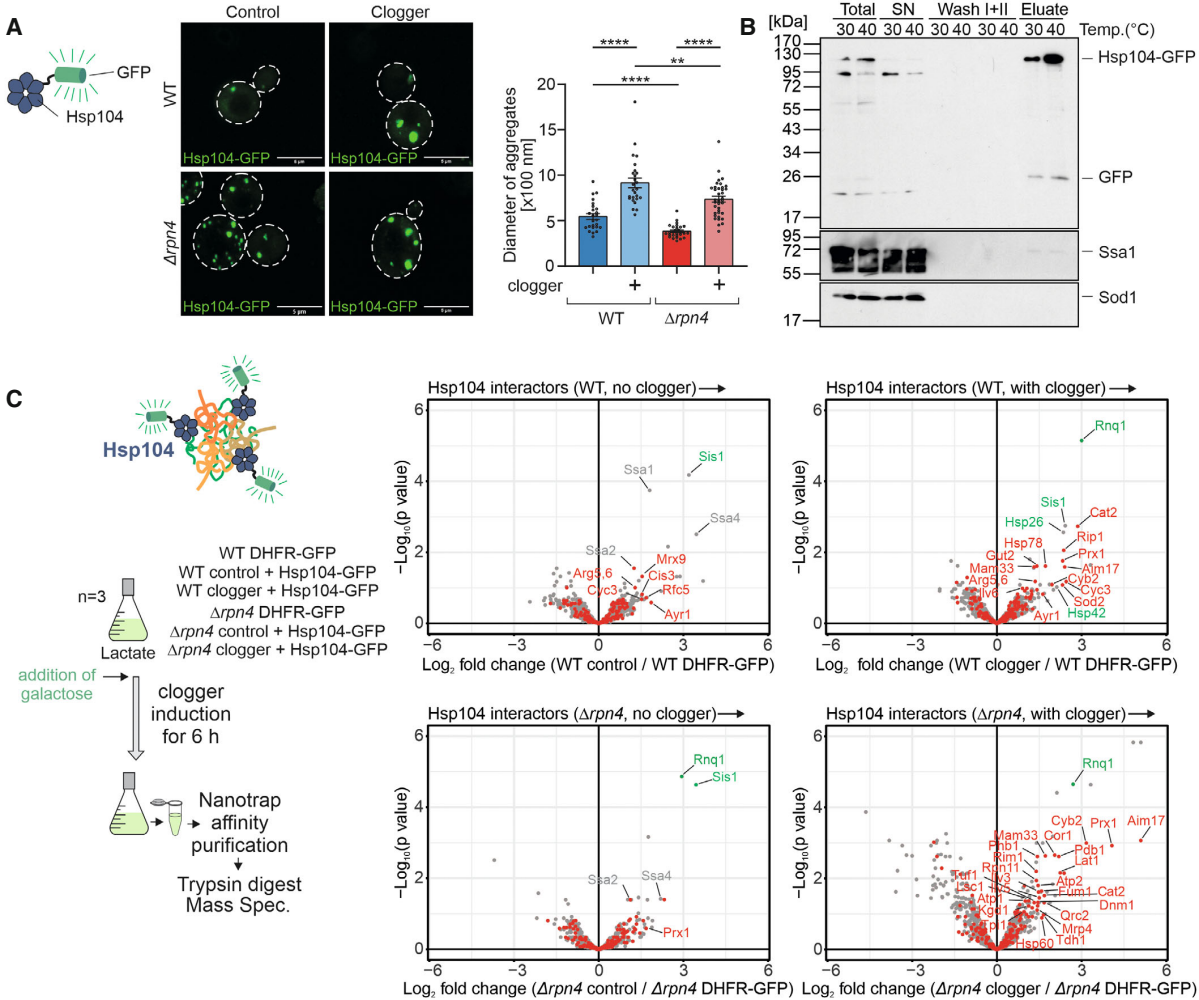
Mitoprotein‐induced stress induces the formation of MitoStores in the cytosol Cytosolic DHFR (control) or the clogger protein *b*_2_‐DHFR was expressed in wild‐type and *Δrpn4* cells for 4.5 h. The distribution of constitutively expressed Hsp104‐GFP was visualized by fluorescence microscopy. Diameter of Hsp104‐GFP‐bound aggregates was quantified. Data are displayed as mean ± standard deviations from *n* = 36 independent biological replicates. Significance was assessed using a two‐sided, paired Student's *t*‐test. *P*‐values are indicated as asterisks **P ≤ 0.01, ****P ≤ 0.0001. See also Fig EV4A for quantification of the number of aggregates per cell. Scale bars, 5 μm.Hsp104‐GFP was purified on nanotrap sepharose in wild‐type cells that were grown at the indicated temperatures. SN, supernatant representing the nonbound fraction. The signals for Ssa1 and Sod1 are shown for control.Hsp104‐GFP or DHFR‐GFP were co‐expressed with clogger or the DHFR control in wild‐type and *Δrpn4* cells. Clogger expression was induced with 0.5% galactose for 6 h before cells were lysed, and the extracts were subjected to nanotrap sepharose chromatography. Purified proteins were analyzed by tandem mass spectrometry. Mitochondrial proteins are highlighted in red, specific chaperones and Rnq1 are indicated in gray or green. The Hsp104 data point was omitted for better scaling of the figure. See Dataset EV2 for details. Source data are available online for this figure.

**Figure EV4:**
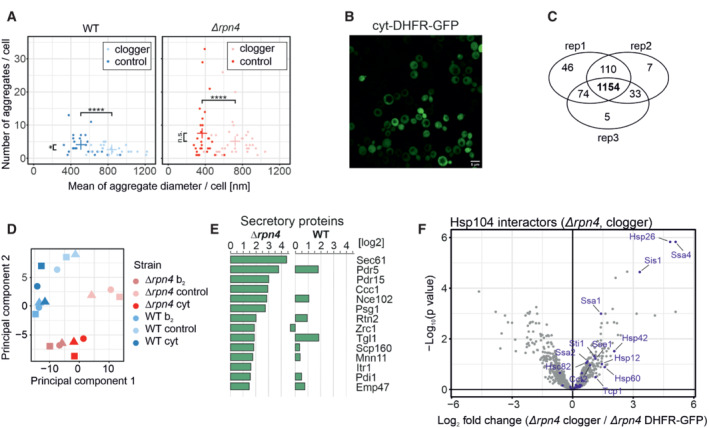
Deletion of Rpn4 remodels the cytosolic chaperone network Quantification of aggregate formation in WT and *Δrpn4* cells expressing either the clogger or a cytosolic DHFR as a control for 4.5 h. Plotted are the mean diameters of the individual aggregates in nm, as well as the number of aggregates per cell. Data are displayed as mean ± standard deviations from *n* = 36 independent biological replicates. Significance was assessed using a two‐sided, paired Student's *t*‐test. *P*‐values are indicated as asterisks. asterisks **P ≤ 0.01, ****P ≤ 0.0001.After expressing the cytosolic DHFR‐GFP construct for 4.5 h in WT cells, the expression was visualized by confocal microscopy. Scale bars, 10 μm.Identification overview of the mass spectrometry. 1,154 proteins were measured in all samples of the three replicates.Principal component analysis. Rpn4 deletion and clogger expression caused specific changes in the proteome. The different shapes of the individual data points indicate the three biological replicates.Purified proteins of the secretory pathway interacting with Hsp104‐GFP in *Δrpn4* expressing the clogger normalized to the control.Hsp104 interactors in the presence of clogger relative to interactors of DHFR‐GFP. Chaperones are shown in purple. Source data are available online for this figure.

We grew Hsp104‐GFP‐expressing cells in lactate medium to mid‐log phase and then shifted them to galactose‐containing medium for 4.5 h. In wild‐type cells, Hsp104‐GFP was mainly evenly distributed throughout the cells and, in addition, formed a small number of aggregates (4 on average; Fig 3A). Upon clogger induction, the number of aggregates per cell did not increase (Fig EV4A), but the mean diameters of Hsp104‐GFP‐bound foci increased significantly 1.7‐fold (545–915 nm; Fig 3A). A comparable size increase (382–724 nm) was also observed in *Δrpn4* cells, which generally contained more aggregates (7.5 on average).

To identify the content of the Hsp104‐GFP‐bound structures, we developed a purification procedure by affinity chromatography with nanotraps after gentle lysis of yeast cells (Fig 3B). For comparison of the Hsp104‐GFP pulldown, we used isolates of a cytosolic DHFR‐GFP fusion protein as this soluble and nontoxic protein accumulated to similar levels as Hsp104‐GFP in the yeast cytosol (Fig EV4B). We then identified the co‐eluted proteins by quantitative proteomics (Figs 3C and EV4C and D).

Established interactors of the Hsp104 disaggregase such as the yeast prion protein Rnq1 or the J‐domain protein Sis1 (Krobitsch & Lindquist, 2000; Higurashi *et al*, 2008; Kryndushkin *et al*, 2011; Ho *et al*, 2019; Wyszkowski *et al*, 2021) were pulled down with Hsp104‐GFP under all conditions tested. Upon clogger induction, a large number of mitochondrial proteins were co‐isolated with Hsp104, indicating that the increase in the size of the Hsp104‐bound granules is, at least in part, due to the association of mitochondrial proteins with these structures. In the absence of Rpn4, this association was even further increased indicating that precursor proteins that escape proteolytic degradation in the cytosol can be efficiently incorporated into these dedicated structures. These structures are reminiscent to structures that were previously observed and sometimes described as a specific type of stress granules, Q‐bodies, or cytoQs (Miller *et al*, 2015; Böckler *et al*, 2017; Grousl *et al*, 2018; Nowicka *et al*, 2021; Shakya *et al*, 2021; Xiao *et al*, 2021). Owing to the striking acquisition of nuclear‐encoded mitochondrial proteins in these structures, we termed them MitoStores.

### MitoStores contain proteins with mitochondrial presequences

**Figure 4:**
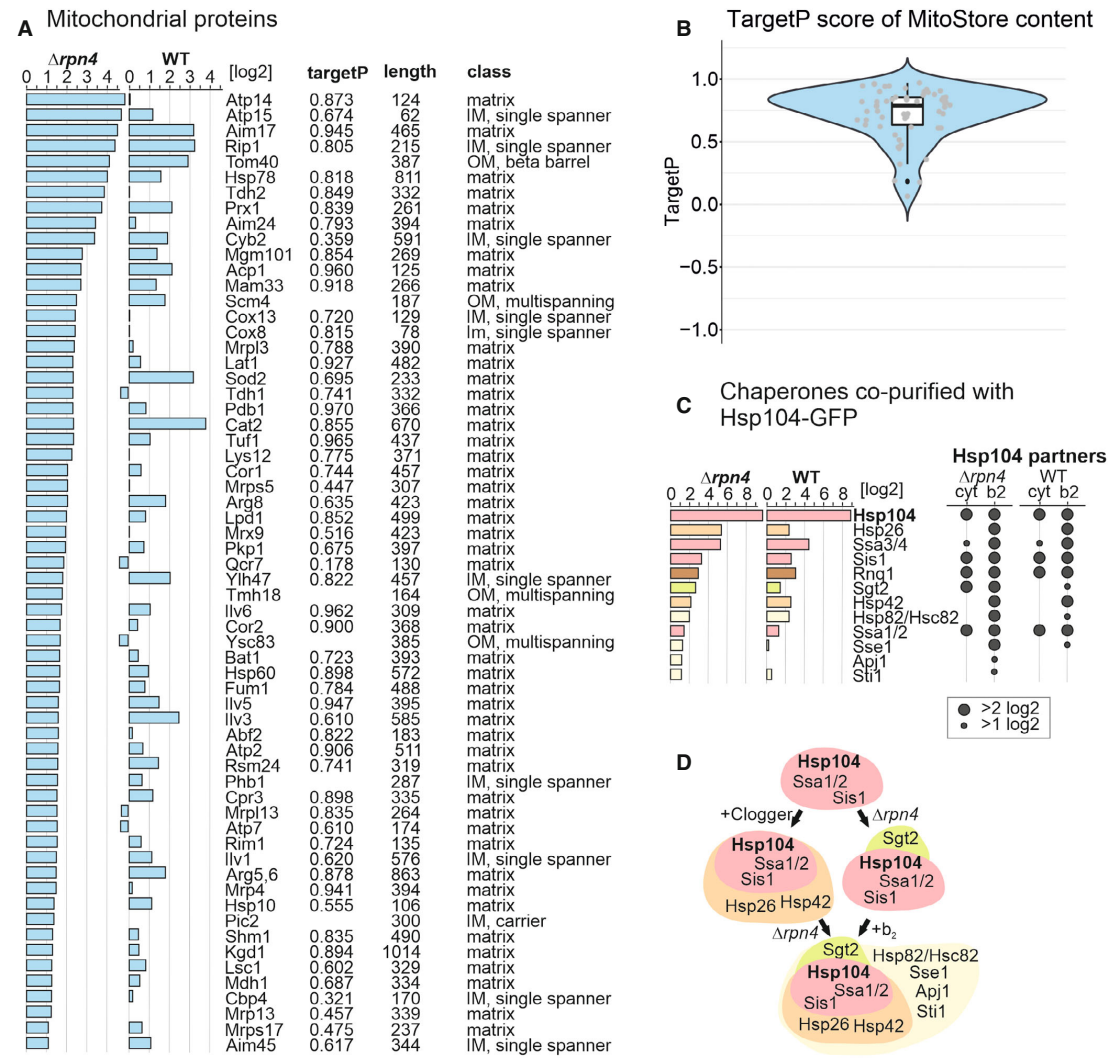
MitoStores contain mitochondrial proteins with N‐terminal matrix‐targeting sequences A
Mitochondrial precursor proteins co‐purified with Hsp104‐GFP upon clogger expression in *Δrpn4* and wild‐type cells. Proteins were listed according to their enrichment in the Hsp104‐GFP sample in comparison to the DHFR‐GFP control. Scores for matrix‐targeting probabilities (targetP; Emanuelsson *et al*, 2000) and mitochondrial location of the proteins are shown. IM, inner membrane; OM, outer membrane. TargetP values were calculated for matrix and presequence‐containing IM proteins.B
MitoStore clients are characterized by high probability scores for mitochondrial targeting. The central band denotes the median, the box denotes the first and third quartiles, and the whiskers extend to the largest value no further than 1.5 times the interquartile range from the box.C, D
Chaperones co‐isolated on the nanotrap beads with the Hsp104‐GFP protein (in comparison to DHFR‐GFP) in the presence of the clogger or the control. Hsp26, Hsp42, and Sse1 are co‐isolated with Hsp104 in the presence of clogger but not upon expression of the DHFR control.

Next, we analyzed the Hsp104‐associated proteins in more detail (Fig 4A). To this end, we listed proteins that were considerably enriched in the Hsp104‐GFP pulldowns (in comparison to the DHFR‐GFP control). Many mitochondrial proteins were found as clients of Hsp104 both in the absence or presence of Rpn4. Examples of this group are Aim17, Mam33, Prx1, Ilv3, Arg5,6, Sod2, or Rip1. These proteins are thus associated with MitoStores irrespective of the proteasomal activity. However, another large group of mitochondrial proteins was only found in MitoStores when the upregulation of proteasomal activity was prevented by the deletion of Rpn4. These proteins, including Pdb1, Atp14, Tdh2, Cox8, Cox13, or Mrp3, are presumably tightly controlled by the proteasome and only found as clients of Hsp104 in *Δrpn4* cells. Interestingly, several of these proteins were recently found recently in a proteome‐wide screen for mitochondrial proteins that accumulate in the cytosol when the mitochondrial import is blocked by treatment with an uncoupler of the membrane potential (Shakya *et al*, 2021).

We noticed that almost all mitochondrial proteins found in the MitoStores contained matrix‐targeting sequences resulting in high scores in mitochondrial targeting prediction programs such as targetP (Emanuelsson *et al*, 2000; Fig 4A and B). This is surprising as only about 60% of all mitochondrial proteins contain N‐terminal matrix‐targeting sequences, which are absent from many proteins of the outer membrane, the IMS, and the inner membrane (Vögtle *et al*, 2009; Morgenstern *et al*, 2017). Apparently, the presence of mitochondrial targeting sequences enhances the incorporation of clients into MitoStores, either by a specific uptake mechanism or by the kinetic properties of their cytosolic targeting (Fig 4B). Thus, Hsp104 particularly binds to proteins with mitochondrial presequences under these conditions, whereas proteins of the outer membrane, the IMS, or carrier proteins of the inner membrane were considerably underrepresented. Also, several proteins of the endomembrane system were enriched in this dataset, however, by far not as many as mitochondrial proteins (Fig EV4E).

**Figure EV5:**
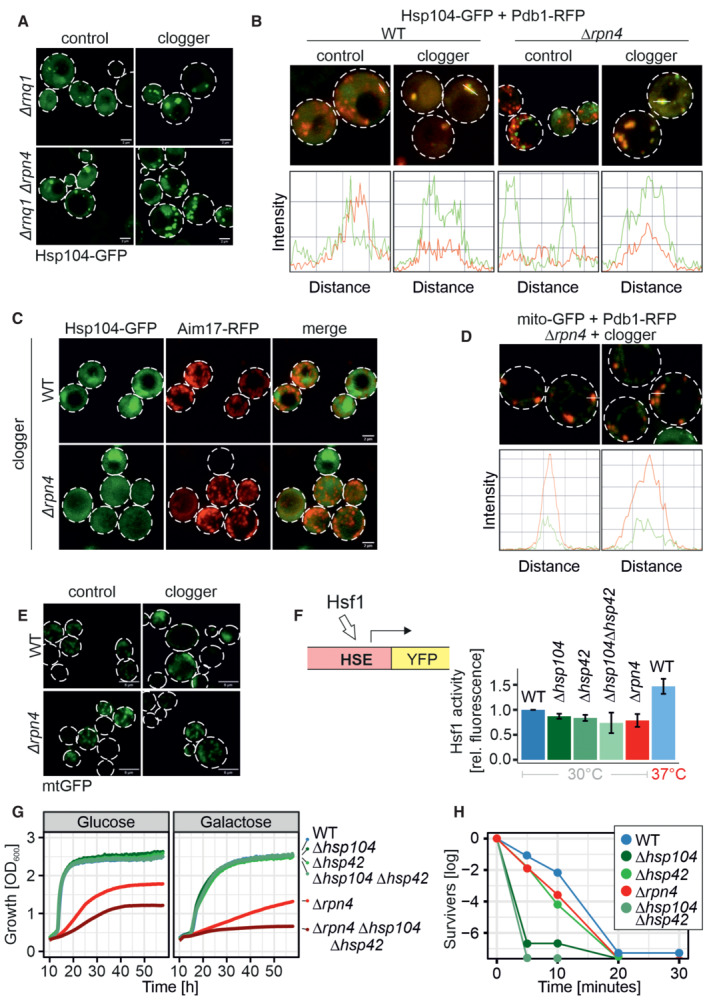
MitoStore formation depends on the Hsp42/Hsp104 chaperone system Microscopy of *Δrnq1* and *Δrpn4Δrnq1* cells expressing Hsp104‐GFP constitutively and the clogger or cytosolic control for 4.5 h. Please note that the formation of MitoStores did not depend on the yeast prion protein Rnq1. Scale bars, 2 μm.Quantification of the colocalization of Hsp104‐GFP with Pdb1‐RFP after clogger expression for 4.5 h.After 4.5 h of clogger and Aim17‐RFP expression, WT and *Δrpn4* cells were incubated for 4 h in the absence of galactose. Whereas Hsp104‐GFP was distributed throughout the cytosol after this chase period, Aim17‐RFP showed a distribution pattern characteristic of mitochondrial proteins.Quantification of the colocalization of mito‐GFP with Pdb1‐RFP after clogger expression for 4.5 h in the *Δrpn4* mutant.Cells expressing mitochondria‐targeted GFP (mtGFP) were grown in lactate medium containing 0.5% galactose for 4.5 h and visualized. Whereas *Δrpn4* cell mtGFP shows a typical mitochondrial staining even upon clogger expression, the fluorescence in the clogger‐expressing wild‐type cells is more patchy and less defined. Scale bars, 5 μm.Expression of a heat shock response reporter that uses a YFP cassette expressed under the control of a heat shock element in the different strains indicated (Zheng *et al*, 2016; Boos *et al*, 2019). Cells were grown in lactate containing medium. Note that induction to 37°C activated the heat shock promoter, but the deletion of Hsp42, Hsp104, or Rpn4 under the nonchallenged conditions of the experiment did not. Data are displayed as mean ± standard deviations from *n* = 3 independent biological replicates.Cells of the indicated strains were grown to log phase and diluted in glucose or galactose medium to 0.1 OD_600_. Growth was monitored upon constant agitation at 37°C, respectively.Cells were grown in lactate to mid‐log phase before 50°C heat stress was performed for the indicated time points. After each time aliquots were removed, and the number of living cells was assessed by a plating assay. Source data are available online for this figure.

Several factors of the cytosolic chaperone system were co‐eluted with Hsp104‐GFP, and some of these proteins were strongly enriched upon clogger induction (Figs 4C and D, and EV4F). Most strikingly, the small heat shock proteins Hsp26 and Hsp42 co‐eluted with Hsp104‐GFP once mitochondrial precursors accumulated in the cytosol, suggesting that these small heat shock proteins (Mogk & Bukau, 2017; Haslbeck *et al*, 2020) are MitoStore constituents. Other proteins, such as the prion protein Rnq1, were recovered with Hsp104‐GFP independently of clogger expression. Rnq1 might therefore be part of another Hsp104‐bound complex. Consistently, Rnq1 was also not necessary for MitoStore formation (Fig EV5A).

### Mitochondrial precursors and Hsp104 colocalize in MitoStores

**Figure 5:**
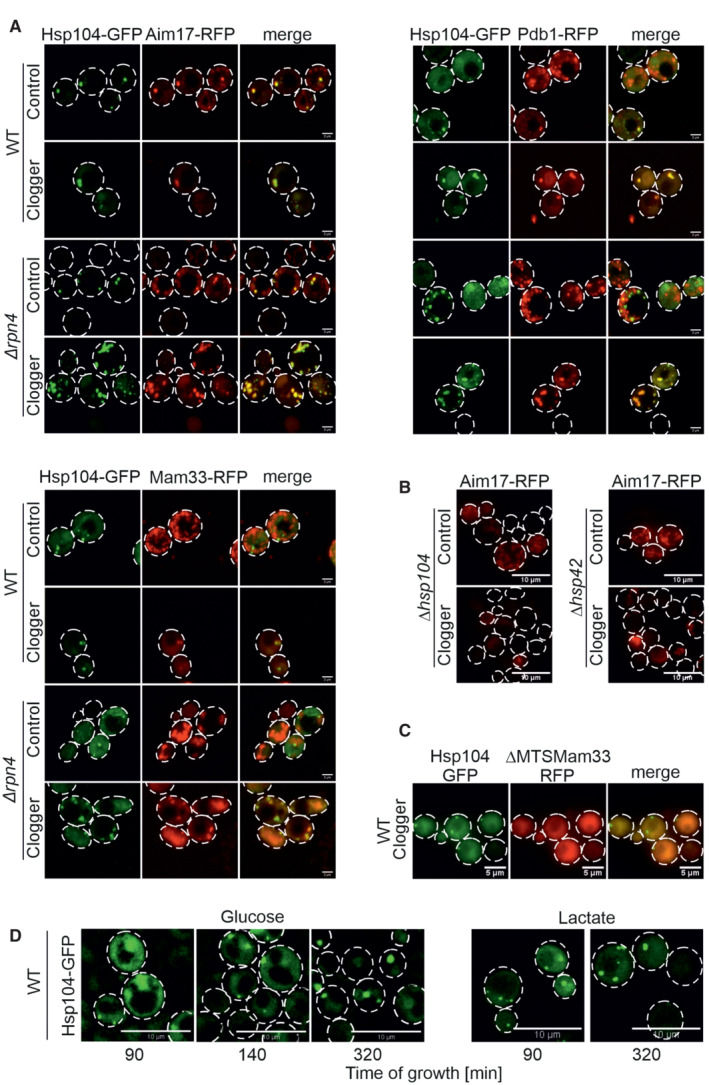
Mitochondrial proteins colocalize with Hsp104 in MitoStores The mitochondrial proteins Pdb1, Mam33, and Aim17 were co‐expressed for 4.5 h with clogger as fusions with the red fluorescent protein RFP (mCherry). Fluorescence microscopic images of the indicated mitochondrial proteins visualized 4.5 h after induction of the clogger or cytosolic DHFR for control. For quantification of colocalization see Fig EV5D. Scale bars, 2 μm.Mutants lacking Hsp104 and Hsp42 were transformed with Aim17‐expressing plasmids and grown to mid‐log phase in lactate medium. Clogger expression was induced for 4.5 h with 0.5% galactose before microscopy was performed. Please note that in the absence of Hsp42 and Hsp104, no Aim17‐RFP‐containing granules are formed. Scale bars, 10 μm.Wild‐type cells were transformed with plasmids for the simultaneous expression of the clogger and an N‐terminally truncated version of Mam33‐RFP (both under control of *GAL* promoter) together with constitutively expressed Hsp104‐GFP (*TPI* promoter). The cells were grown on lactate plus 0.5% galactose‐containing medium for 4.5 h. The distribution of the fluorescent proteins was visualized by microscopy. Scale bars, 5 μm.Wild‐type cells expressing Hsp104‐GFP were grown on glucose or lactate medium to log phase. Cells were diluted and visualized by life cell imaging. Please note, that in glucose media, Hsp104‐GFP is initially dispersed in cells and only aggregates when cells become stationary. By contrast, Hsp104‐GFP‐containing granules are frequent on lactate medium even at the log phase. See Movie EV1 for life cell imaging of glucose‐grown cells. Scale bars, 10 μm. Source data are available online for this figure.

To verify that mitochondrial precursors and Hsp104 indeed colocalize in cytosolic granules, we fused the three mitochondrial Hsp104 clients Aim17, Pdb1, and Mam33 to the red fluorescent protein (RFP) mCherry and co‐expressed them with Hsp104‐GFP (Figs 5A and B, and EV5B). Upon clogger induction, the RFP signal formed defined punctae that colocalized with Hsp104‐GFP. Whereas colocalization of Aim17‐RFP with Hsp104‐GFP was observed even without clogger induction, Pdb1 and Mam33 were only found in cytosolic granules when import was slowed down by clogger expression. Thus, the association of individual proteins with cytosolic MitoStores likely depends on the individual protein and on the prevailing cellular conditions.

The presence of Hsp104 and Hsp42 was crucial for MitoStore formation (Fig 5B). Furthermore, the removal of the N‐terminal mitochondrial targeting sequence from Mam33 prevented its incorporation into MitoStores (Fig 5C), again emphasizing the relevance of the mitochondrial presequence as a critical determinant for MitoStore uptake. It should be noted that Hsp104‐GFP‐containing granules were frequently found in yeast cells even in the absence of the clogger or uncouplers. Upon growth on glucose, Hsp104‐GFP granules were only observed when cells reached the stationary phase; however, on nonfermentable carbon sources, which induce the expression of mitochondrial proteins, yeast cells form Hsp104‐GFP‐bound granules also in the log phase (Fig 5D). This is consistent with previous reports (Sanchez *et al*, 1992).

### MitoStores facilitate the productive import into mitochondria

**Figure 6:**
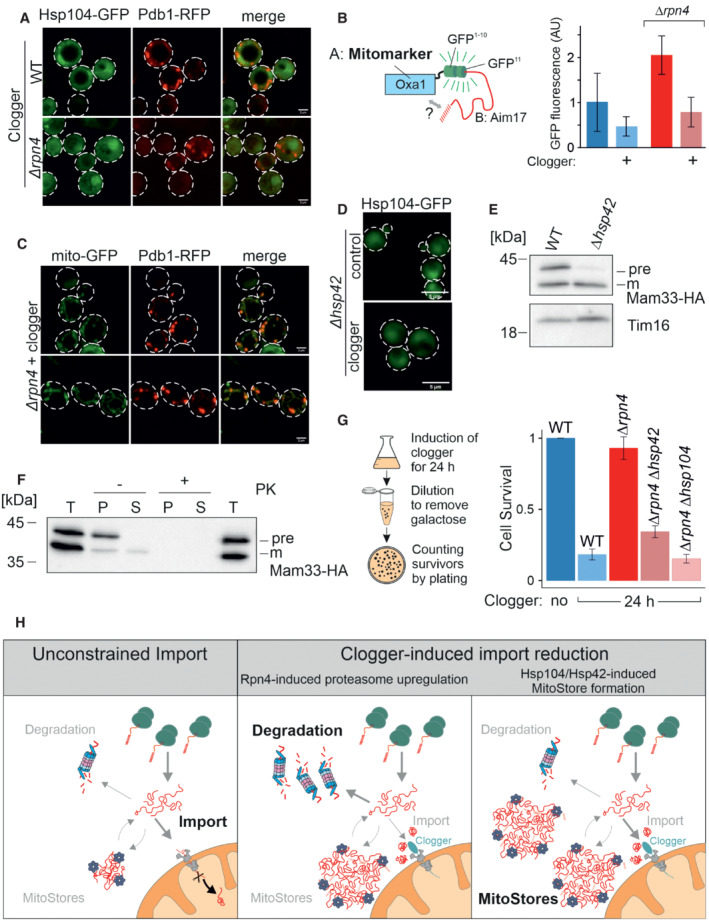
MitoStores form proximity to mitochondria After 4.5 h of the galactose‐induced expression of clogger and Pdb1‐RFP, cells were incubated for 4 h in the absence of galactose. Whereas Hsp104‐GFP was distributed throughout the cytosol after this chase period, Pdb1‐RFP showed a distribution pattern characteristic of mitochondrial proteins. Scale bars, 2 μm.To verify that stored proteins are chased into the mitochondrial matrix, Aim17‐GFP11 was co‐expressed with a clogger for 4.5 h in cells containing mitochondria with Oxa1‐GFP1‐10. Cells were washed in galactose‐free buffer and further incubated for 4 h before fluorescence was measured to quantify the matrix‐localized Aim17 proteins. Data are displayed as mean ± standard deviations from *n* = 3 independent biological replicates.Clogger was expressed for 4.5 h before colocalization of MitoStores (visualized by Pdb1‐RFP), and mitochondria (stained with matrix‐residing mito‐GFP) were assessed. Scale bars, 2 μm.Clogger was expressed for 4.5 h in *Δhsp42* cells before Hsp104‐GFP was visualized. Please note that in the absence of Hsp42, no aggregates were detected. Scale bars, 5 μm.Clogger and Mam33‐HA were co‐expressed for 4.5 h in wild‐type and *Δhsp42* cells. Mitochondria were isolated and subjected to Western blotting to detect mature (m) and precursor (pre) forms of Mam33‐HA. The matrix protein Tim16 served as a loading control.The mitochondria used for (E) were treated with proteinase K (PK) to remove surface‐exposed proteins. Mitochondrial membranes were lysed with NP‐40 and soluble (S) and aggregated (P) proteins were separated by centrifugation. T, total.To measure the toxicity of clogger expression, clogger proteins were induced for 24 h with lactate medium containing 0.5% galactose in the mutants indicated. Aliquots were removed, and the number of living cells was assessed by a plating assay on glucose‐containing plates. The resistance of *Δrpn4* to clogger expression depends on Hsp104 and Hsp42. Data are displayed as mean ± standard deviations from *n* = 3 independent biological replicates.Schematic representation of cytosolic MitoStores. Source data are available online for this figure.

Next, we asked whether cells could resolve MitoStores once the stress conditions are relieved. To this end, we shifted clogger‐inducing cells after 4.5 h continuous growth on galactose (i.e., inducting conditions) to lactate medium lacking galactose for 4 h. Once clogger induction was stopped, Hsp104 was again evenly distributed throughout the cytosol, and Pdb1‐RFP and Aim17‐RFP displayed a punctate distribution characteristic for mitochondria (Figs 6A and EV5C). To verify that mitochondrial proteins that are synthesized during the clogger‐mediated import inhibition reach the mitochondria once the block is relieved, we developed an assay relying on a split‐GFP approach. To this end, we constitutively expressed the first 10 beta sheets of superfolder GFP in mitochondria (Oxa1‐GFP^1‐10^) and co‐expressed the 11^th^ beta sheet of GFP fused to Aim17 under a galactose‐regulatable promoter. Thus, this Aim17‐GFP^11^ fusion protein was co‐expressed with the clogger for 4.5 h, before galactose was removed, and cells were further incubated for 4 h. As shown in Fig 6B, clogger expression reduced the access of this reporter into mitochondria; nevertheless, considerable amounts of the protein were still imported after the chase reaction (about 50% of the control sample without the clogger). Interestingly, in the absence of Rpn4, considerably higher levels were imported into mitochondria indicating that Aim17 is indeed stabilized in the cytosol in an import‐competent fashion, particularly if proteasome upregulation is prevented.

Next, we addressed the localization of MitoStores within cells. Double staining of mitochondrial GFP and Pdb1‐RFP indicated that MitoStores reside in close proximity to mitochondria (Figs 6C and EV5D). We even observed nonimported Mam33‐HA precursor co‐isolating with purified wild‐type mitochondria from clogger‐stressed cells. In MitoStore‐deficient *Δhsp42* cells, no Mam33‐HA precursor was co‐isolated with mitochondria under these conditions (Fig 6E). This precursor was protease‐accessible and found in high‐speed pellets after solubilizing mitochondrial membranes with detergent, indicating that MitoStores remain associated with the mitochondrial surface even after cellular fractionation procedures (Fig 6F).

Since Hsp42 was crucial for the formation of MitoStores (Fig 6D), we tested whether deletion of Hsp42 suppressed the clogger resistance observed in *Δrpn4* mutants (Fig 6G). Indeed, *Δrpn4* cells required Hsp42 and Hsp104 to survive clogger stress (Fig 6G) and even maintained a wild‐type‐like mitochondrial network under these conditions (Fig EV5E). Deletion of Hsp42, Hsp104, or Rpn4 does not induce a heat shock response upon nonchallenged growth conditions (30°C, glucose, Fig EV5F). Thus, Hsp42 and Hsp104 protect against clogger stress in the absence of Rpn4, which is reminiscent of their role during heat stress (Fig EV5G and H). In summary, we describe here that the cytosol of yeast cells has the capacity to transiently store mitochondrial matrix proteins in Hsp42‐induced and Hsp104‐bound structures, which we name MitoStores (Fig 6H). These structures reduce the toxic effects arising from nonimported mitochondrial precursor proteins.

## Discussion

To our surprise, we observed that yeast cells lacking Rpn4 can tolerate the accumulation of mitochondrial precursors in their cytosol even better than wild‐type cells. Instead of degrading these precursors, these cells efficiently and selectively package precursors into cytosolic granules that are bound by the disaggregase Hsp104. Hsp42 and Hsp104 are upregulated in *Δrpn4* cells even before clogger expression explaining the increased resistance against mitoprotein‐induced stress of these cells.

These MitoStores resemble stress granules or Q‐bodies in their overall appearance (Escusa‐Toret *et al*, 2013; Roth & Balch, 2013; Hill *et al*, 2017; Samant *et al*, 2018; Eisele *et al*, 2021); however, they largely contain matrix‐destined mitochondrial precursor proteins and precursors of secretory proteins. We observed that MitoStores transiently accumulate during phases in which the synthesis of mitochondrial proteins exceeds the capacity of the mitochondrial import system. This is most extreme upon clogger‐mediated competitive inhibition of protein import, but also apparent when cells are grown on nonfermentative carbon sources such as lactate or glycerol, as well as at late stages on glucose media when cells undergo a diauxic shift to switch to ethanol consumption (Di Bartolomeo *et al*, 2020).

The specific upregulation of Hsp42 and Hsp104 in Rpn4‐deficient cells indicates that MitoStore formation and proteasome‐mediated degradation are two alternative strategies to deal with the accumulation of mitochondrial precursors in the cytosol (Fig 6H). The balance of both processes is not clear, but the immediate Rpn4‐mediated induction of the proteasome system (Boos *et al*, 2019) indicates that cytosolic precursors are under tight proteolytic control. The proteasome plays a direct and crucial role in the removal of stalled import intermediates from the mitochondrial outer membrane translocase or from other cellular nonproductive binding sites (Itakura *et al*, 2016; Weidberg & Amon, 2018; Mårtensson *et al*, 2019; Mohanraj *et al*, 2019; Basch *et al*, 2020; Murschall *et al*, 2020; Shakya *et al*, 2021; Rodl *et al*, 2023). Hence, proteasomal upregulation appears to be the primary and immediate cellular response to mitoprotein‐induced stress conditions. Nevertheless, cells actively promote the formation of MitoStores in the cytosol by employing small heat shock proteins such as Hsp26 and Hsp42 as nucleation sites (Mogk & Bukau, 2017; Haslbeck *et al*, 2020), presumably to sequester precursors to reduce their toxicity. Interestingly, MitoStores apparently accommodate a specific subset of proteins, in particular proteins with N‐terminal presequences. The proteins that are typically used in the field to detect import defects by detection of precursor proteins in Western Blots, such as Rip1, Mdj1, Hsp60, or Sod2, were all found highly enriched in MitoStores (Gallas *et al*, 2006; Sinha *et al*, 2010; Mårtensson *et al*, 2019; Poveda‐Huertes *et al*, 2020; Nowicka *et al*, 2021). Strikingly, members of the carrier family were not detected in MitoStores even though this group of inner membrane proteins is very abundant (Morgenstern *et al*, 2017) and exhibits a highly toxic potential (Wang & Chen, 2015; Backes *et al*, 2021; Xiao *et al*, 2021). Apparently, the import of carrier proteins deviates from that of presequence‐containing precursors at the early stages of the cytosolic targeting. Consistently, previous studies reported that these two groups employ different factors of the cytosolic chaperone system during their targeting to mitochondria (Komiya *et al*, 1997; Hansen *et al*, 2018; Opalinski *et al*, 2018; Backes *et al*, 2021). These variations in the premitochondrial life of mitochondrial proteins might explain the differences between the mitoprotein‐induced stress reactions towards accumulating presequence‐containing proteins (typically referred to as UPRam) and accumulating carriers (referred to as mitochondrial precursor over‐accumulation stress, mPOS; Wang & Chen, 2015; Wrobel *et al*, 2015). The accumulation of carriers is apparently much more toxic, potentially because they are not sequestered in MitoStores (Hoshino *et al*, 2019; Pouikli *et al*, 2021; Wang *et al*, 2022) but rather interfere with nonmitochondrial membranes (Xiao *et al*, 2021).

We observed that MitoStores are transient in nature and dissolve once the mitoprotein‐inducing stress conditions are ameliorated. This is different from what was reported for IPODs or aggresomes, which are toxic aggregates that form for example from insoluble polyQ proteins (Tyedmers *et al*, 2010; Spokoini *et al*, 2012; Hill *et al*, 2014; Schlagowski *et al*, 2021). Whereas IPOD formation uses the yeast prion Rnq1 for nucleation, we observed no relevance of Rnq1 for the formation of MitoStores (Fig EV5A). We therefore favor the idea that MitoStores are benign structures, which safeguard cellular proteostasis during overload of the mitochondrial import system or metabolic remodeling.

Our observation that mitochondrial precursor proteins can be selectively incorporated into dedicated storage granules in the cytosol adds another step into the cascade of reactions that mediate the targeting and import of mitochondrial proteins. Further studies will have to elucidate the relevance of cytosolic factors and of the ubiquitination machinery to control the stress‐induced formation and poststress resolution of MitoStores, and to characterize their physical and functional association with proteins of the mitochondrial surface.

## Materials and Methods

### Strains and growth conditions

The yeast strains and plasmids used in this study are described in detail in Tables EV1 and EV2, respectively. Unless specified, all strains were derived from YPH499*Δarg4* (MATa *ura3 lys2 ade2 trp1 his3 leu2*).

The strains were grown at 30°C either in yeast complete medium (YP) containing 1% (w/v) yeast extract, 2% (w/v) peptone, and 2% (w/v) of the respective carbon source or in minimal synthetic respiratory medium containing 0.67% (w/v) yeast nitrogen base and 2% lactate as carbon source. To induce the clogger from the *GAL1* promoter, 0.5% galactose was added. The temperature‐sensitive strains and their corresponding wild‐type were grown at 25°C and the phenotype was induced with growth at 37°C.

### Growth assays and viability tests

For spot analysis, the respective yeast strains were grown in liquid‐rich or synthetic media. Total yeast cells equivalent to 0.5 OD_600_ were harvested at the exponential phase. The cells were washed in sterile water and subjected to 10‐fold serial dilutions. From each dilution, 3 μl was spotted on the respective media followed by incubation at 30°C or 37°C. Pictures were taken after different days of the growth.

Growth curves were performed in a 96‐well plate, using the automated ELx808™ Absorbance Microplate Reader (BioTek®). The growth curves started at 0.1 OD_600_ and the OD_600_ were measured every 10 min for 72 h at 30°C or 37°C. The mean of technical triplicates was calculated and plotted in R.

For viability assays, yeast cells were pregrown in lactate medium at 30°. At the mid‐log phase (OD_600_ 0.6–0.8), either clogger expression was induced with 0.5% galactose or the cells were exposed to 50°C for the indicated time points. After each timepoint, 100 μl of 0.001 OD_600_ cell suspension was equally plated on lactate plates. The plates were incubated at 30°C for 3 days to assess the number of colonies, which survived the mitoprotein‐induced or, respectively, the heat stress.

### Antibodies

The antibodies against Sod1, GFP, and Djp1 were raised in rabbits using recombinant purified proteins. The antibody against Pre6 was kindly gifted by Dieter Wolf (University of Stuttgart, Germany). The antibody against mouse DHFR was kindly provided by Martin van der Laan (Saarland University, Germany). The antibodies against Rip1 and Mdj1 were kindly gifted by Thomas Becker (University of Freiburg, Germany). The antibody against Ssa1 was kindly provided by Günter Kramer (ZMBH Heidelberg, Germany). The antibody against Tim16 was kindly gifted by Dejana Mokranjac (LMU München, Germany). The horseradish‐peroxidase coupled HA antibody was ordered from Roche (Anti‐HA‐Peroxidase, High Affinity 3F10, #12013819001). The secondary antibodies were ordered from Biorad (Goat Anti‐Rabbit IgG (H + L)‐HRP Conjugate #172‐1019). Antibodies were diluted in 5% (w/v) nonfat dry milk‐TBS (Roth T145.2) with the following dilutions: anti‐Sod1 1:1,000, anti‐Rip1 1:750, anti‐Mdj1 1:125, anti‐Ssa1 1:10,000, anti‐Rabbit 1:10,000.

### YFP reporter assays

The PACE‐YFP or HSE‐YFP reporter gene was integrated into the *LEU2* locus of the yeast genome. Cells were grown to mid‐log phase (OD 0.6–0.8) under noninducing conditions and shifted to inducing conditions by the addition of 0.5% galactose for 4.5 h. Four OD_600_ of cells were harvested by centrifugation (20,000 *g*, 3 min, RT) and resuspended in 400 μl H_2_O. 100 μl of cell suspension was transferred to flat‐bottomed black 96‐well imaging plates (BD Falcon, Heidelberg, Germany) in technical triplicates. Cells were sedimented by gentle spinning (30 g, 5 min, RT), and fluorescence (excitation 497 nm, emission 540 nm) was measured using a ClarioStar Fluorescence Platereader (BMG‐Labtech, Offenburg, Germany). The corresponding wild‐type strain not expressing YFP was used for background subtraction of autofluorescence.

Fluorescence intensities were normalized to the value obtained from the wild‐type empty vector control in each of three independent biological replicates. Statistical significance was assessed using a paired one‐tailed Student's *t*‐test where a *P*‐value of below 0.05 was interpreted as a significant difference.

### Cell lysates

For whole cell lysates, yeast strains were cultivated in selective lactate media to mid‐log phase. Clogger expression was induced by the addition of 0.5% galactose. For recovery assays, the medium was exchanged for a noninducing lactate medium. After the indicated timepoints 4 OD_600_ were harvested by centrifugation (17,000 *g*, 2 min). Cells were washed with water and resuspended in reducing loading buffer. Cells were transferred to screw‐cap tubes containing 1 mm glass beads. Cell lysis was performed using a FastPrep‐24 5G homogenizer (MP Biomedicals, Heidelberg, Germany) with 3 cycles of 30 s, speed 8.0 m/s, 120 s breaks, glass beads. Lysates were boiled at 96°C for 5 min, centrifuged (17,000 *g*, 2 min), and stored at −20°C until further use. The equal amount of OD_600_ was loaded on an SDS gel.

### Isolation of mitochondria

Mitochondria were isolated essentially as described before (Hansen *et al*, 2018). For the isolation of mitochondria cells were grown in selective lactate media. Cells were harvested (1,800 *g*, JA10 Beckmann rotor, 5 min, RT) in the exponential phase. After a washing step, cells were treated for 10 min with 2 ml per g wet weight MP1 buffer (10 mM Tris–pH unadjusted and 100 mM DTT) at 30°C. After washing with 1.2 M sorbitol, yeast cells were resuspended in 6.7 ml per g wet weight MP2 buffer (20 mM KPi buffer pH 7.4, 1.2 M sorbitol, 3 mg/g wet weight zymolyase from Seikagaku Biobusiness) and incubated for 1 h at 30°C. Spheroplasts were collected via centrifugation at 4°C and resuspended in ice‐cold homogenization buffer (13.4 ml/g wet weight; 10 mM Tris–pH 7.4, 1 mM EDTA pH 8, 0.2% fatty acid‐free bovine serum albumin (BSA), 1 4 mM PMSF, 0.6 M sorbitol). Spheroplasts were disrupted by 10 strokes with a cooled glass potter. Cell debris was removed via centrifugation at 1,200 *g* in a JA10 Beckmann rotor. The supernatant was centrifuged for 12 min at 11,000 *g* to collect mitochondria. Mitochondria were resuspended in 10 ml of ice‐cold SH buffer (0.6 M sorbitol, 20 mM Hepes pH 7.4) and centrifuged again at 1,300 *g* in a JA25.50 Beckmann rotor to remove residual cell debris. To harvest the mitochondria, the supernatant was again centrifuged for 12 min at 12,000 *g*. The amount of mitochondria was determined using the Bradford assay.

### Sample preparation and mass spectrometric identification of proteins

For the quantitative comparison of proteomes of *Δrpn4* and WT cells expressing the clogger, the expression of b_2_‐DHFR was induced for 6 h and compared with control cells expressing cytosolic DHFR. 10 OD_600_ of cells were harvested at each time point by centrifugation (17,000 *g*, 3 min, 2°C), washed with prechilled water, snap‐frozen in liquid nitrogen, and stored at −80°C. Cells lysates were prepared in lysis buffer (50 mM Tris–pH 7.5, 2% (w/v) SDS, Tablets mini EDTA‐free protease inhibitor (Roche)) using a FastPrep‐24 5G homogenizer (MP Biomedicals, Heidelberg, Germany) with 3 cycles of 30 s, speed 8.0 m/s, 120 s breaks, glass beads. Lysates were boiled for 5 min at 96°C and centrifuged (17,000 *g*, 3 min, 2°C). Protein concentrations were determined using the Pierce BCA Protein Assay (Thermo Scientific, #23225). 20 μg of each lysate was subjected to an in‐solution tryptic digest using a modified version of the Single‐Pot Solid‐Phase‐enhanced Sample Preparation (SP3) protocol (Hughes *et al*, 2014, 2019). Here, lysates were added to Sera‐Mag Beads (Thermo Scientific, #4515‐2105‐050250, 6515‐2105‐050250) in 10 μl 15% formic acid and 30 μl of ethanol. Binding of proteins was achieved by shaking for 15 min at room temperature. SDS was removed by four subsequent washes with 200 μl of 70% ethanol. Proteins were digested with 0.4 μg of sequencing grade modified trypsin (Promega, #V5111) in 40 μl Hepes/NaOH, pH 8.4 in the presence of 1.25 mM TCEP and 5 mM chloroacetamide (Sigma‐Aldrich, #C0267) overnight at room temperature. Beads were separated and washed with 10 μl of an aqueous solution of 2% DMSO, and the combined eluates were dried down. In total three biological replicates were prepared (*n* = 3). Each replicate included samples of control and clogger‐expressing cells (in total six samples per replicate). Peptides were reconstituted in 10 μl of H_2_O and reacted with 80 μg of TMT10plex (Thermo Scientific, #90111) (Werner *et al*, 2014) label reagent dissolved in 4 μl of acetonitrile for 1 h at room temperature. Excess TMT reagent was quenched by the addition of 4 μl of an aqueous solution of 5% hydroxylamine (Sigma, 438227). Peptides were mixed to achieve a 1:1 ratio across all TMT channels. Mixed peptides were desalted on home‐made StageTips containing Empore C_18_ disks (Rappsilber *et al*, 2007) and subjected to an SCX fractionation on StageTips into three fractions, followed by additional cleanup on C_18_ StageTips. The resulting 12 fractions were then analyzed by LC–MS/MS on a Q Exactive Plus (Thermo Scientific) as previously described (Rappsilber *et al*, 2007).

For IP mass spectrometry, cells were incubated in lactate medium containing galactose to induce the expression of the clogger. After 6 h 10 OD_600_ cells were harvested by centrifugation (5,000 *g* for 5 min), washed with prechilled water, snap‐frozen in liquid nitrogen, and stored at −80°C. Cell lysates were prepared in 200 μl ice‐cold lysis buffer (25 mM Tris–HCl pH 7.5, 50 mM KCl, 10 mM MgCl_2_, 5% (v/v) glycerol, 1% Nonidet P‐40, 1 mM DTT, 1 mM PMSF, 1× cOmplete™ Tablets mini EDTA‐free protease inhibitor (Roche), PhosSTOP phosphate inhibitor (Roche)) using a FastPrep‐24 5G homogenizer (MP Biomedicals, Heidelberg, Germany) with 3 cycles of 30 s, speed 8.0 m/s, 120 s breaks, glass beads. Samples were centrifuged at 5,000 *g* for 5 min and transferred to precooled microtubes to get rid of cell debris. 300 μl dilution buffer (10 mM Tris–HCl pH 7.5, 150 mM NaCl, 0.5 mM EDTA, cOmplete™ Tablets mini EDTA‐free protease inhibitor (Roche), 1 mM PMSF) was added. Diluted cell lysates were used for an IP with GFP‐Trap magnetic agarose beads from Chromotek. The GFP‐Trap beads were equilibrated by washing 25 μl of beads slurry with 500 μl ice‐cold dilution buffer and subsequently centrifuged for 2 min at 2,500 *g*. The washing step was repeated two more times. GFP‐tagged proteins (Hsp104 or DHFR, respectively) were bound to GFP‐Trap beads for 1 h at 4°C tumbling end‐over‐end. Samples were centrifuged (2 min at 2,500 *g*), and the supernatant was discarded by using a magnetic rack. Beads were washed 3x with 800 μl ice‐cold wash buffer I (150 mM NaCl, 50 mM Tris–HCl pH 7.5, 5% (v/v) glycerol, 0.05% Nonidet P‐40) and afterwards 2× with 500 μl ice‐cold wash buffer II (150 mM NaCl, 50 mM Tris–HCl pH 7.5, 5% (v/v) glycerol). Samples for Western Blotting were eluted in 1x Laemmli and boiled for 5 min at 90°C. For elution and trypsin digestion of mass spectrometry samples, 50 μl elution buffer I was added (2 M Urea, 50 mM Tris–HCl pH 7.5, 1 mM DTT, 5 ng/μl trypsin) and incubated for 1 h at RT. Then, 1 μl trypsin (15 ng/μl) was added and incubated for 10 min at RT. Samples were centrifuged (2,500 *g*, 2 min) and supernatants were transferred to fresh tubes. 50 μl elution buffer II (2 M Urea, 50 mM Tris–pH 7.5, 5 mM CAA) was added. Samples were incubated ON in the dark at RT. pH of samples was adjusted to pH < 2 with Tri‐fluoroacetic acid. Desalting/reversed‐Phase cleanup with 3xC18 stage tips. Samples were dried down in speed‐vac and resolubilized in 9 μl buffer A (0.1% formic acid in MS grad water) and 1 μl buffer A* (0.1% formic acid, 0.1% TFA in MS grad water). The samples were analyzed by LC–MS/MS on a Q Exactive HF(Thermo Scientific) as previously described (Sridharan *et al*, 2019).

Briefly, peptides were separated using an Easy‐nLC 1200 system (Thermo Scientific) coupled to a Q Exactive HF mass spectrometer via a Nanospray‐Flex ion source. The analytical column (50 cm, 75 μm inner diameter (NewObjective) packed in‐house with C18 resin ReproSilPur 120, 1.9 μm diameter Dr. Maisch) was operated at a constant flow rate of 250 nl/min. For TMT labeled samples, a 3 h gradient was used to elute peptides (Solvent A: aqueous 0.1% formic acid; Solvent B: 80% acetonitrile, 0.1% formic acid). Peptides were analyzed in positive ion mode applying with a spray voltage of 2.3 kV and a capillary temperature of 250°C. MS spectra with a mass range of 375–1,400 *m/z* were acquired in profile mode using a resolution of 120,000 [maximum fill time of 80 ms or a maximum of 3e6 ions (automatic gain control, AGC)]. Fragmentation was triggered for the top 15 peaks with charge 2–8 on the MS scan (data‐dependent acquisition) with a 30 s dynamic exclusion window (normalized collision energy was 32). Precursors were isolated with a 0.7 *m/z* window and MS/MS spectra were acquired in profile mode with a resolution of 60,000 (maximum fill time of 100 ms, AGC target of 1e5 ions, fixed first mass 100 *m/z*).

For label‐free IP‐MS samples, shorter gradients (90 min) were used to elute peptides (Solvent A: aqueous 0.1% formic acid; Solvent B: 80% acetonitrile, 0.1% formic acid). MS spectra with a mass range of 300–1,650 *m/z* were acquired in profile mode using a resolution of 60,000 [maximum fill time of 20 ms or a maximum of 3e6 ions (automatic gain control, AGC)]. Fragmentation was triggered for the top 15 peaks with charge 2–8 on the MS scan (data‐dependent acquisition) with a 30 s dynamic exclusion window (normalized collision energy was 28). Precursors were isolated with a 1.4 *m/z* window and MS/MS spectra were acquired in profile mode with a resolution of 15,000 (maximum fill time of 80 ms, AGC target of 2e4 ions).

### Analysis of mass spectrometry data

The proteomics data were analyzed essentially as described previously (Backes *et al*, 2021): Peptide and protein identification and quantification were done using the MaxQuant software (version 1.6.10.43; Cox & Mann, 2008; Cox *et al*, 2011; Tyanova *et al*, 2016) and a *Saccharomyces cerevisiae* proteome database obtained from Uniprot. 10plex TMT was chosen in Reporter ion MS2 quantification, up to 2 tryptic miss‐cleavages were allowed, and protein N‐terminal acetylation and Met oxidation were specified as variable modifications and Cys carbamidomethylation as fixed modification. The “Requantify” and “Second Peptides” options were deactivated. The false discovery rate was set at 1% for peptides, proteins, and sites; minimal peptide length was seven amino acids.

The output files of MaxQuant were processed using the R programming language. Only proteins that were quantified with at least two unique peptides were considered for the analysis. Moreover, only proteins that were identified in at least two out of three MS runs were kept. A total of 3,550 proteins for the whole cell proteome and a total of 1,154 for the IP passed the quality control filters. Raw signal sums were cleaned for batch effects using limma (Ritchie *et al*, 2015) and further normalized using variance stabilization normalization (Huber *et al*, 2002). Proteins were tested for differential expression using the limma package for the indicated comparison of strains.

A reference list of yeast mitochondrial proteins was obtained from (Morgenstern *et al*, 2017). Gene set enrichment analysis was performed using the Fisher's exact test. A Benjamini–Hochberg procedure was used to account for multiple testing, where this was performed (Benjamini & Hochberg, 1995).

### Fluorescence microscopy

Manual microscopy was performed using Confocal Zeiss LSM 700 (Axio Examiner.D1) system or a Leica Dmi8 Thunder Imager. Images were acquired using a Plan‐Apochromat 63×/1.4 Oil DIC M27 or an HC PL APO100×/1,44 Oil UV objective. Carl Zeiss with Immersol 518 F immersion oil (ne = 1.518) or Immersion Oil Type A 518 F, with the wavelength of 488 nm (GFP) and the ZEN2009 or LAS X software. Further processing of images was performed in Fiji/ImageJ.

### Measurements of fluorescent intensity profiles

To visualize the colocalization of GFP‐ and RFP‐tagged proteins, fluorescent intensity profiles were measured. Therefore, images were acquired as described previously. Acquired images were imported into ZEN 3.3 (blue edition) software, and fluorescent intensity profiles were generated by drawing a line across the object to be analyzed (in this case across GFP or RFP foci). Profiles could then be exported as TIF files.

### Colocalization analysis via Pearson's correlation coefficient calculation

To provide a quantitative statement on the colocalization of RFP‐ and GFP‐tagged proteins, the Pearson's correlation coefficient was calculated. Therefore, images were acquired, and fluorescent intensity profiles were measured as described above. The obtained data table containing the distance in nm and the intensity values of each selected channel was exported as CSV file. Pearson's correlation coefficient *r* was calculated as follows:

$$r=\frac{\sum_{i}\left(G_{i}-\bar{G}\right)\left(R_{i}-\bar{R}\right)}{\sqrt{\sum_{i}{\left(G_{i}-\bar{G}\right)}^{2}}\sqrt{\sum_{i}{\left(R_{i}-\bar{R}\right)}^{2}}}$$

*G*_*i*_ and *R*_*i*_ refer to the intensity values of the green (488 nm) and red (561 nm) channels, respectively, of pixel *i*, whereas *G̅* and *R̅* refer to the mean intensities of the red and green channels of each image. The Pearson's correlation coefficient can assume values between −1 and 1. A positive linear correlation results in a value of 1 and thereby describing perfectly linear related fluorescence intensities of two images. A negative linear correlation results in a value of −1, and no correlation results in a value of 0 (Dunn *et al*, 2011).

### Distance measurement of Hsp104‐GFP foci

Characteristics of aggregates (Hsp104 foci) were quantified by measuring number and two‐dimensional diameter. Therefore, microscopy images were acquired as described previously and imported into ZEN 3.3 (blue edition) software. Two‐dimensional diameters were measured in nm by drawing a line across each Hsp104 foci. For quantification, the number of aggregates per cell was plotted against the mean of two‐dimensional aggregate diameter per cell.

### Cell cycle analysis by flow cytometry

For cell cycle analysis, cells were grown in selective lactate media. Clogger expression was induced for 4.5 h with 0.5% galactose. Cells were harvested (6,000 *g*, 1 min, RT) in the exponential phase. Cells were fixed by resuspending the pellet in 1 ml 70% EtOH and stored rolling overnight at 4°C. Cells were washed with 1 ml H_2_O, the pellet stained with 500 μl FxCycleTM PI/RNase Staining Solution and stored in the dark for 30 min. Flow cytometry analysis was done using 2018 Attune NxT Flow Cytometer (Thermofisher Scientific) with an excitation of 535 nm. Data were then analyzed with FlowJo 10.6.0 software (Tree Star).

### Radioactive *in vivo* labelling of mitochondrial translation products

Cells were grown in lactate medium lacking methionine to exponential phase. ^35^S‐methionine (2 μl of a 22 μCi solution) was added to the cell suspension. Aliquots of 2 OD_600_ of cells were withdrawn after 5 min of incubation at 30°C. Incorporation of radioactive methionine was quenched by the addition of 8 mM cold methionine. Cells were lysed with 0.3 M NaOH, 1% β‐mercaptoethanol, and 3 mM PMSF. Proteins were precipitated with 12% trichloroacetic acid and analyzed by SDS–PAGE and autoradiography.

## Author contributions

**Lena Kra¨mer:** Conceptualization; data curation; formal analysis; validation; investigation; visualization; methodology; writing – original draft; writing – review and editing. **Niko Dalheimer:** Data curation; validation; visualization; writing – review and editing. **Markus Ra¨schle:** Conceptualization; validation; investigation; methodology; writing – review and editing. **Zuzana Storchova¨:** Conceptualization; data curation; writing – review and editing. **Jan Pielage:** Conceptualization; visualization; writing – review and editing. **Felix Boos:** Conceptualization; data curation; formal analysis; validation; investigation; project administration; writing – review and editing. **Johannes M Herrmann:** Conceptualization; data curation; supervision; funding acquisition; visualization; writing – original draft; project administration. Open Access funding enabled and organized by Projekt DEAL.

## Disclosure and competing interests statement

The authors declare that they have no conflict of interest.

## Supporting information



Expanded View Figures PDF

Table EV1

Table EV2

Movie EV1

Dataset EV1

Dataset EV2

Source Data for Expanded View

PDF+

Source Data for Figure 1

Source Data for Figure 2

Source Data for Figure 3

Source Data for Figure 5

Source Data for Figure 6

## Data Availability

The mass spectrometry proteomics data (see also Tables EV1 and EV2) have been deposited to the ProteomeXchange Consortium via the PRIDE (Perez‐Riverol *et al*, 2019) partner repository with the dataset identifier PXD035762 (http://www.ebi.ac.uk/pride/archive/projects/PXD035762).
