# Supplementary material for: MitoStores: chaperone‐controlled protein granules store mitochondrial precursors in the cytosol
Source: EMBO J. 2023 Jan 27;42(7):e112309. doi: 10.15252/embj.2022112309 (PMC10068336; doi:10.15252/embj.2022112309)
Supplement: Supplementary file 1 — Expanded View Figures PDF [file EMBJ-42-e112309-s014.pdf]

Expanded View Figures

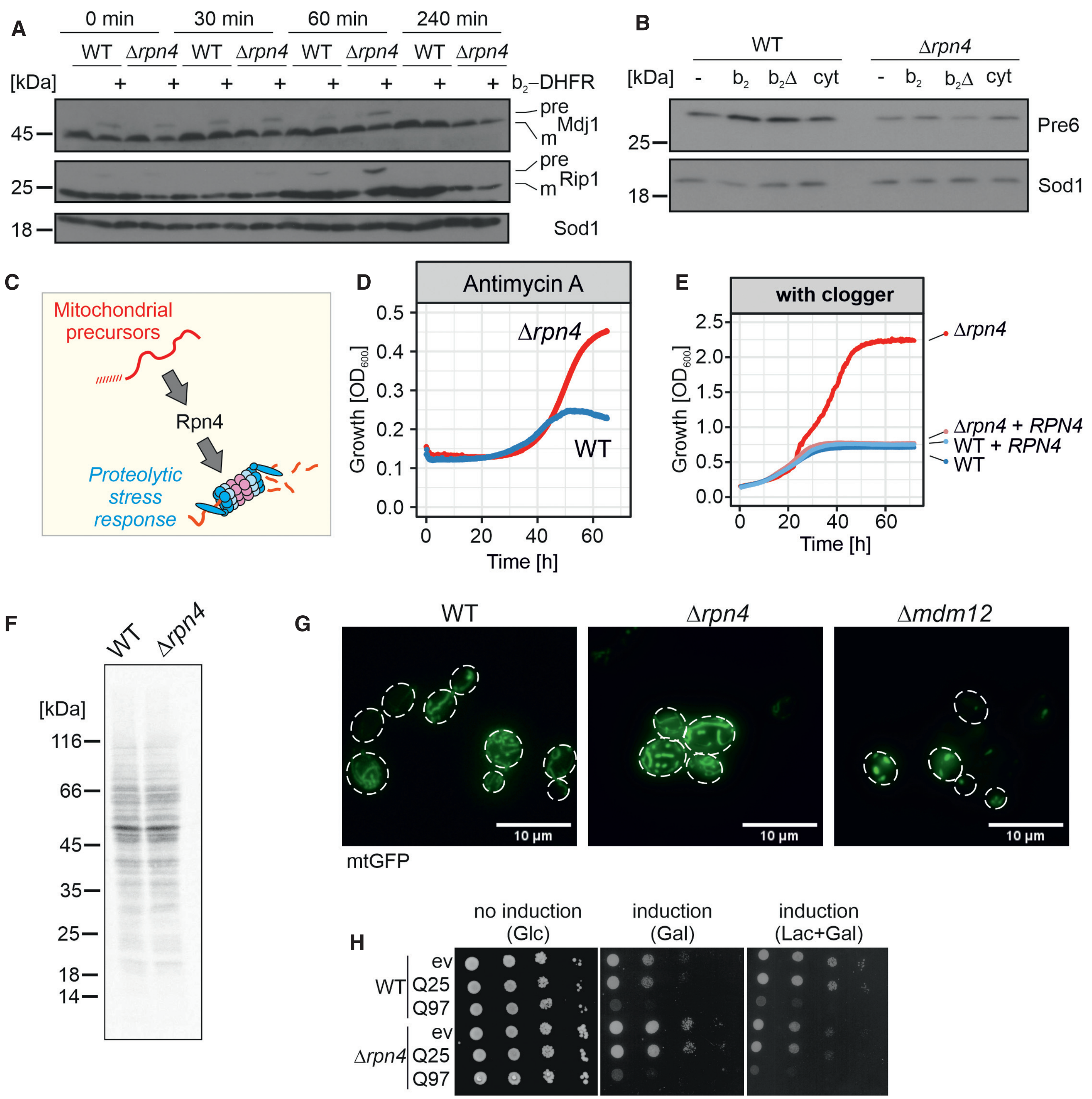

Figure EV1.

Figure EV1. Rpn4-dependent proteasome induction.

A After 4.5 h of clogger expression in wild-type and *Δrpn4* cells, the medium was exchanged for a noninducing lactate medium. Precursor (pre) and mature (m) forms of the mitochondrial proteins Mdj1 and Rip1 were visualized by Western blotting. Sod1 was used as a loading control.

B After 4.5 h expression of clogger and control, protein levels of the proteasome subunit Pre6 were visualized by Western Blotting. Please note, that in *Δrpn4* cells, clogger expression does not lead to Pre6 induction. Sod1 served as a loading control.

C Schematic representation of Rpn4-dependent proteasome induction after mitoprotein-induced stress.

D Cells of the indicated strains were grown to log phase and diluted in lactate medium to 0.1 OD_{600}. After the addition of 100 μg/ml antimycin A, growth was monitored upon constant agitation. Since antimycin A loses its activity over time, cells start growing once the membrane potential reaches a level that allows mitochondrial biogenesis. *Δrpn4* cells escaped the antimycin A-mediated growth inhibition more efficiently than wild-type cells.

E Clogger-expressing wild-type and *Δrpn4* cells harboring empty or Rpn4-expression plasmids were grown over time in lactate plus 0.5% galactose medium.

F Wild-type and *Δrpn4* cells were grown in lactate medium to log phase. 2 μl of 22 μCi of ^{35}S-methionine was added to the cell suspension. After 5 min, cells were harvested, lysed, and subjected to SDS gel electrophoresis and autoradiography.

G The indicated strains were transformed with plasmids expressing mitochondria-targeted GFP (mtGFP). The mitochondrial network was visualized by microscopy upon growth on galactose. Cells lacking Mdm12 were used as a control for a strain showing defective mitochondrial network formation (Dimmer *et al*, 2002). Scale bars, 10 μm.

H Yeast cells expressing a fusion protein of the N-terminal region of human huntingtin with different lengths of glutamine residues (Q25-GFP and Q97-GFP, respectively; Schlagowski *et al*, 2021) and GFP under control of the galactose-inducible promoter, or a control for comparison were grown to mid-log phase on noninducing glucose medium. Ten-fold serial dilutions were dropped on glucose (no induction), galactose (induction), or lactate with 0.5% galactose medium (induction).

Source data are available online for this figure.

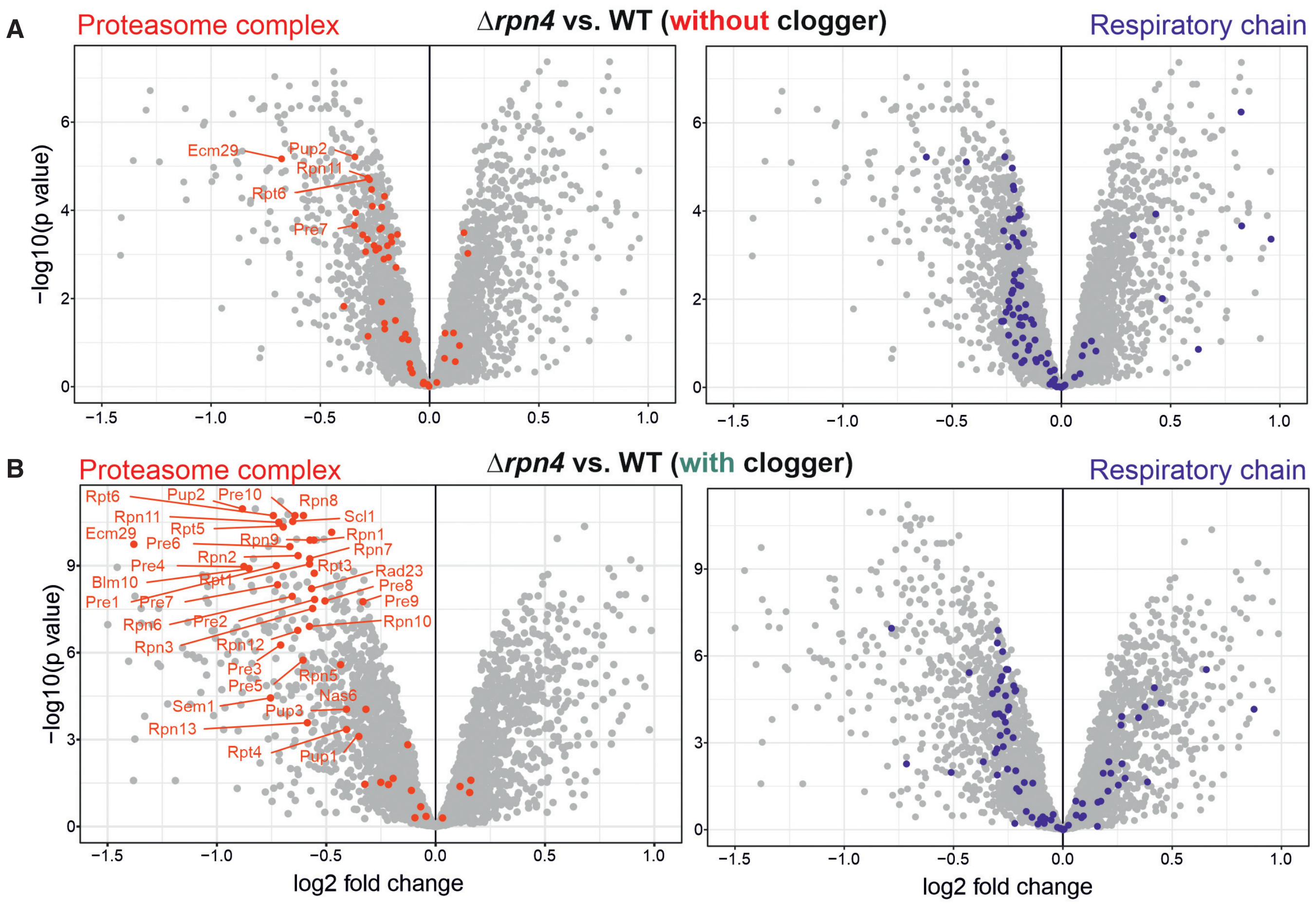

Figure EV2.

© 2023 The Authors

Figure EV2. Rpn4 deletion leads to a reduction in proteasomal and respiratory proteins.

A, B Comparison of the proteome of wild-type and *Δrpn4* cells before and after clogger expression for 6 h. Positions of proteins of the proteasome and the respiratory chain systems are indicated in red and purple, respectively (Morgenstern *et al*, 2017; Boos *et al*, 2019). Please note especially the proteasomal proteins show a lower abundance in *Δrpn4* cells.

Figure EV3. Protein aggregation in the yeast cytosol can be efficiently measured by visualization of the distribution of Hsp104-GFP.

A Yeast cells expressing the temperature-sensitive, aggregation-prone proteins luciferase^{ts}-RFP (Luc^{ts}-RFP) and Ubc9^{ts}-RFP under galactose-inducible promoters together with constitutively expressed Hsp104-GFP or Hsp104^{Y662A}-GFP were grown to mid-log phase on lactate medium. Ten-fold serial dilutions were dropped on lactate (no induction) or galactose medium (induction) and incubated at 25°C or 37°C as indicated.

B Cells of the indicated strains were grown to log phase and diluted in lactate (no induction) or galactose (induction) to 0.1 OD. Growth was monitored upon constant agitation at 28°C and 37°C, respectively.

C After 2 h of Luc^{ts}-RFP or Ubc9^{ts}-RFP expression together with constitutive Hsp104-GFP expression at 37°C (induction), cells were incubated for 4 h in the absence of galactose at 30°C (rescue). Scale bars, 2 µm.

D Fluorescence intensity profiles of cells marked with an asterisk in (C). The measured area is indicated with a white line.

E After 4 h growth on lactate medium (no induction) or galactose medium (induction), the protein level of the proteasomal protein Pre6 was visualized by Western Blotting. Djp1 was used as a loading control.

F Schematic representation of the interaction of aggregation-prone proteins with Hsp104.

Source data are available online for this figure.

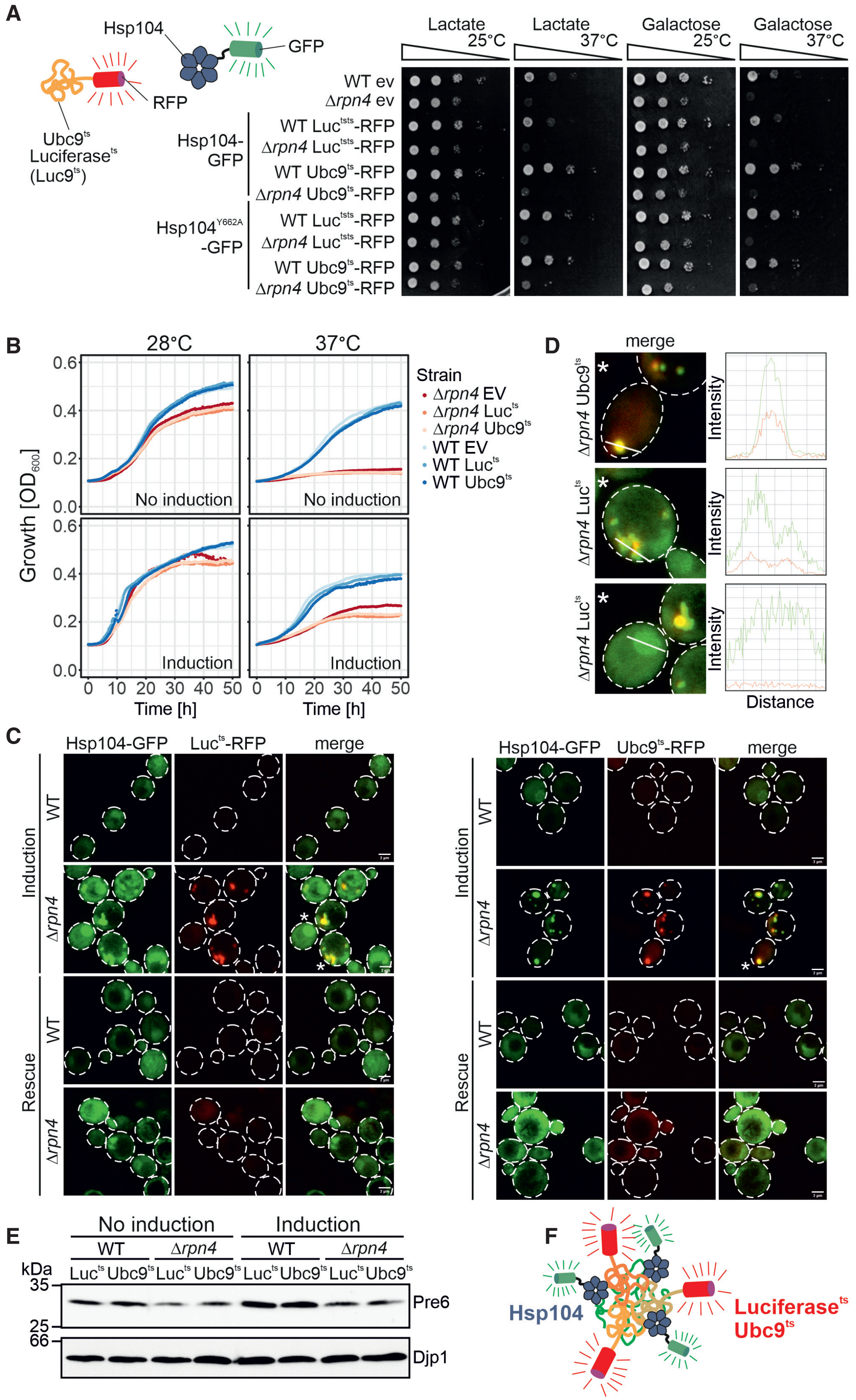

Figure EV3.

© 2023 The Authors

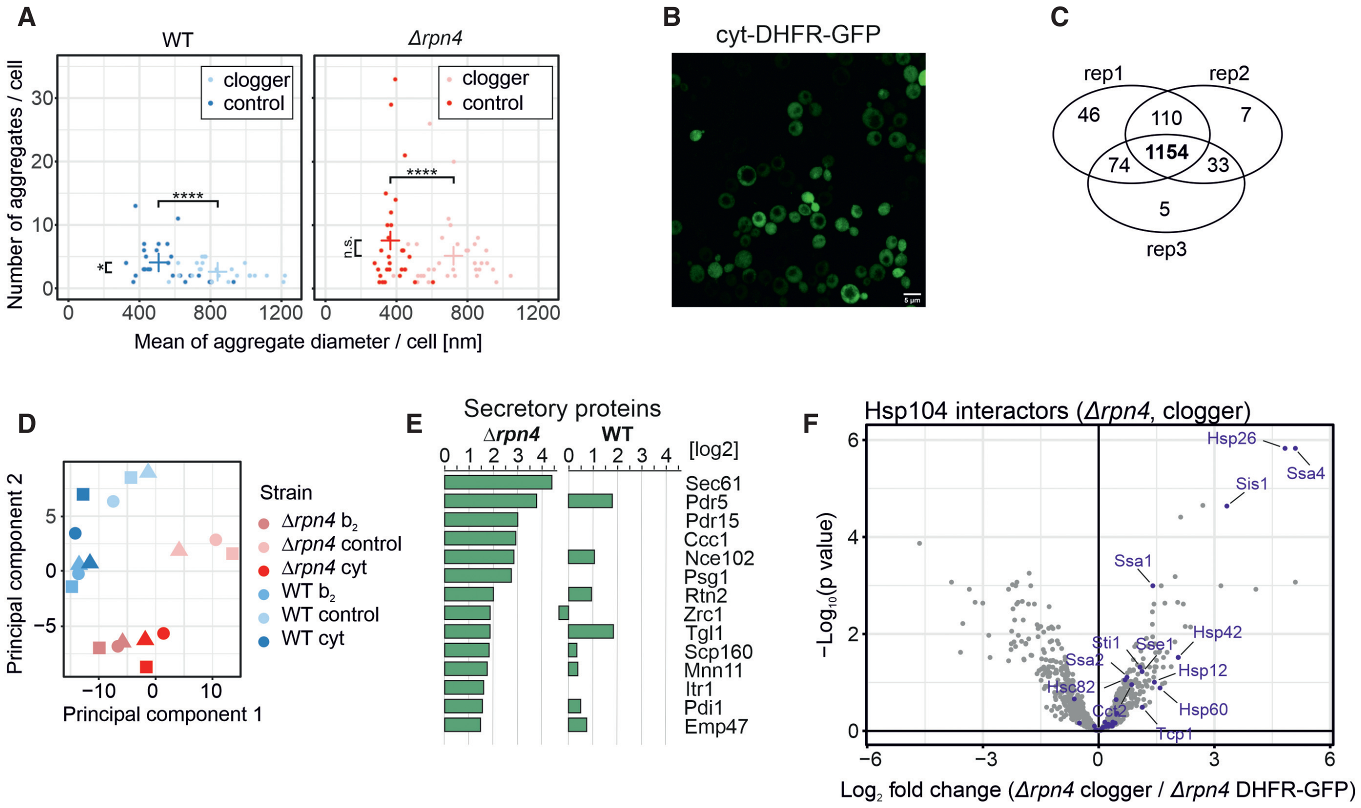

Figure EV4. Deletion of Rpn4 remodels the cytosolic chaperone network.

A Quantification of aggregate formation in WT and *Δrpn4* cells expressing either the clogger or a cytosolic DHFR as a control for 4.5 h. Plotted are the mean diameters of the individual aggregates in nm, as well as the number of aggregates per cell. Data are displayed as mean ± standard deviations from $n = 36$ independent biological replicates. Significance was assessed using a two-sided, paired Student's *t*-test. *P*-values are indicated as asterisks. asterisks $^{**}P \leq 0.01$, $^{****}P \leq 0.0001$.

B After expressing the cytosolic DHFR-GFP construct for 4.5 h in WT cells, the expression was visualized by confocal microscopy. Scale bars, 10 μm.

C Identification overview of the mass spectrometry. 1,154 proteins were measured in all samples of the three replicates.

D Principal component analysis. Rpn4 deletion and clogger expression caused specific changes in the proteome. The different shapes of the individual data points indicate the three biological replicates.

E Purified proteins of the secretory pathway interacting with Hsp104-GFP in *Δrpn4* expressing the clogger normalized to the control.

F Hsp104 interactors in the presence of clogger relative to interactors of DHFR-GFP. Chaperones are shown in purple.

Source data are available online for this figure.

© 2023 The Authors

Figure EV5. MitoStore formation depends on the Hsp42/Hsp104 chaperone system.

A Microscopy of *Δrnq1* and *Δrpn4Δrnq1* cells expressing Hsp104-GFP constitutively and the clogger or cytosolic control for 4.5 h. Please note that the formation of MitoStores did not depend on the yeast prion protein Rnq1. Scale bars, 2 μm.

B Quantification of the colocalization of Hsp104-GFP with Pdb1-RFP after clogger expression for 4.5 h.

C After 4.5 h of clogger and Aim17-RFP expression, WT and *Δrpn4* cells were incubated for 4 h in the absence of galactose. Whereas Hsp104-GFP was distributed throughout the cytosol after this chase period, Aim17-RFP showed a distribution pattern characteristic of mitochondrial proteins.

D Quantification of the colocalization of mito-GFP with Pdb1-RFP after clogger expression for 4.5 h in the *Δrpn4* mutant.

E Cells expressing mitochondria-targeted GFP (mtGFP) were grown in lactate medium containing 0.5% galactose for 4.5 h and visualized. Whereas *Δrpn4* cell mtGFP shows a typical mitochondrial staining even upon clogger expression, the fluorescence in the clogger-expressing wild-type cells is more patchy and less defined. Scale bars, 5 μm.

F Expression of a heat shock response reporter that uses a YFP cassette expressed under the control of a heat shock element in the different strains indicated (Zheng *et al*, 2016; Boos *et al*, 2019). Cells were grown in lactate containing medium. Note that induction to 37°C activated the heat shock promoter, but the deletion of Hsp42, Hsp104, or Rpn4 under the nonchallenged conditions of the experiment did not. Data are displayed as mean ± standard deviations from $n = 3$ independent biological replicates.

G Cells of the indicated strains were grown to log phase and diluted in glucose or galactose medium to 0.1 OD_{600}. Growth was monitored upon constant agitation at 37°C, respectively.

H Cells were grown in lactate to mid-log phase before 50°C heat stress was performed for the indicated time points. After each time aliquots were removed, and the number of living cells was assessed by a plating assay.

Source data are available online for this figure.

 © 2023 The Authors

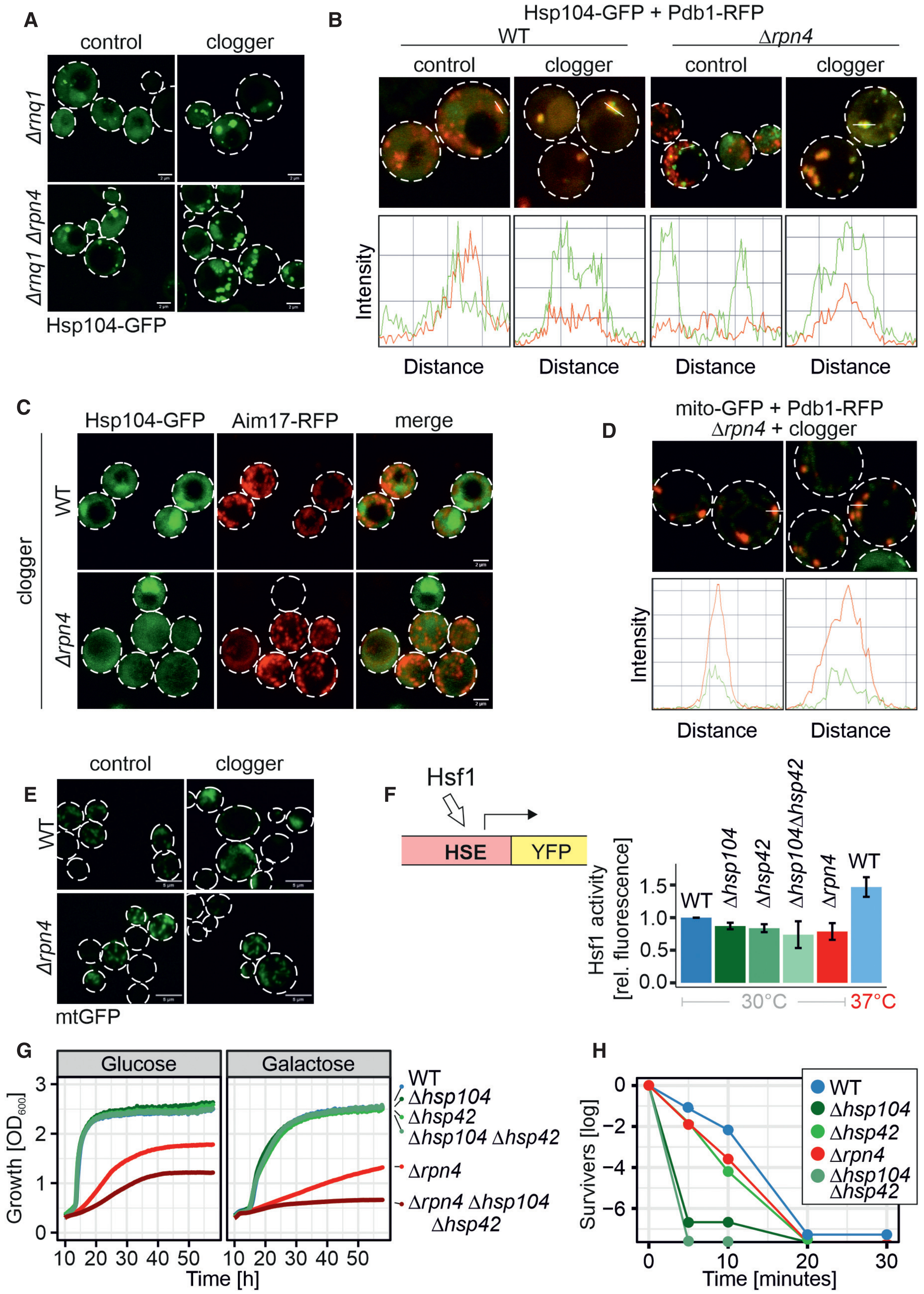
A
control
clogger
Δrnq1
Δrnq1 Δrpn4
Hsp104-GFP
B
Hsp104-GFP + Pdb1-RFP
WT
Δrpn4
control
clogger
control
clogger
Intensity
Distance
Distance
Distance
Distance
C
Hsp104-GFP
Aim17-RFP
merge
clogger
WT
Δrpn4
D
mito-GFP + Pdb1-RFP
Δrpn4 + clogger
Intensity
Distance
Distance
E
control
clogger
WT
Δrpn4
mtGFP
F
Hsf1
HSE
YFP
Hsf1 activity [rel. fluorescence]
WT
Δhsp104
Δhsp42
Δhsp104Δhsp42
Δrpn4
WT
1.5
1.0
0.5
0.0
30°C
37°C
G
Glucose
Galactose
Growth [OD600]
3
2
1
0
10 20 30 40 50
10 20 30 40 50
Time [h]
WT
Δhsp104
Δhsp42
Δhsp104 Δhsp42
Δrpn4
Δrpn4 Δhsp104 Δhsp42
H
Survivers [log]
0
−2
−4
−6
0
10
20
30
Time [minutes]
WT
Δhsp104
Δhsp42
Δrpn4
Δhsp104 Δhsp42

Figure EV5.
