# Supplementary material for: MitoStores: chaperone‐controlled protein granules store mitochondrial precursors in the cytosol
Source: EMBO J. 2023 Jan 27;42(7):e112309. doi: 10.15252/embj.2022112309 (PMC10068336; doi:10.15252/embj.2022112309)
Supplement: Supplementary file 4 — Movie EV1 [file EMBJ-42-e112309-s011.zip › EMBOJ-2022-112309_MovieEV1/Legend_MovieEV1.docx]

**Movie. EV1. MitoStores are build under non-fermentive conditions**

Wild type cells expressing Hsp104-GFP were grown on glucose to log phase. Cells were diluted to OD_600_ 0.1 and visualized by life cell imaging. Please note, that Hsp104-GFP is initially dispersed in cells but aggregates when cells become stationary.
