# Supplementary figures and images for: MitoStores: chaperone‐controlled protein granules store mitochondrial precursors in the cytosol
Source: EMBO J. 2023 Jan 27;42(7):e112309. doi: 10.15252/embj.2022112309 (PMC10068336; doi:10.15252/embj.2022112309)

**Fig.1B**

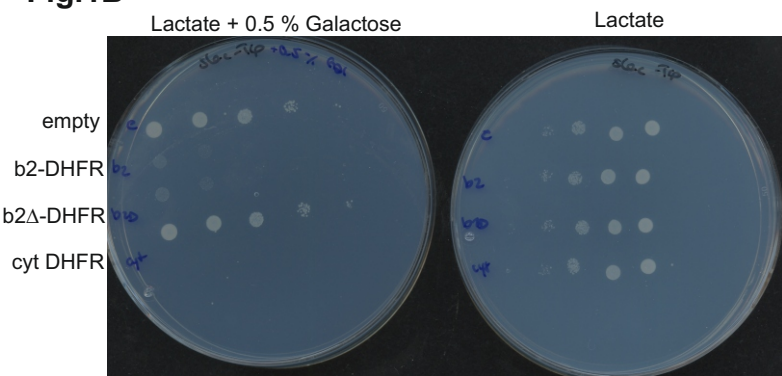

Supplement: Supplementary file 9 — Source Data for Figure 1 [file EMBJ-42-e112309-s013.zip › SourceData_Figure 1/SourceData_Figure 1B.pdf]

Fig.1E

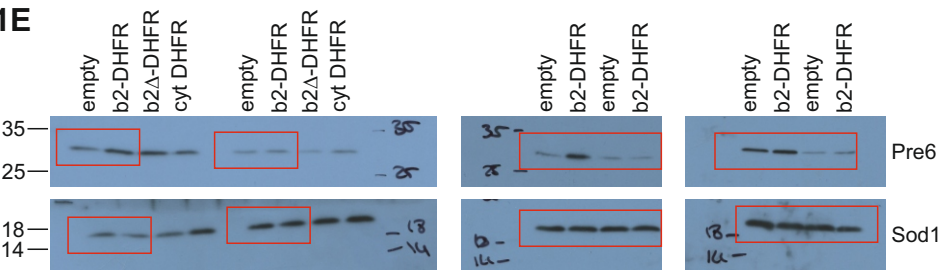

Supplement: Supplementary file 9 — Source Data for Figure 1 [file EMBJ-42-e112309-s013.zip › SourceData_Figure 1/SourceData_Figure 1E.pdf]

Fig.2A

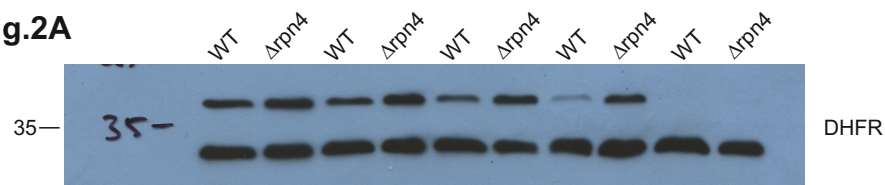

Supplement: Supplementary file 10 — Source Data for Figure 2 [file EMBJ-42-e112309-s006.zip › SourceData_Figure 2/SourceData_Figure 2A.pdf]

**Fig.3A**

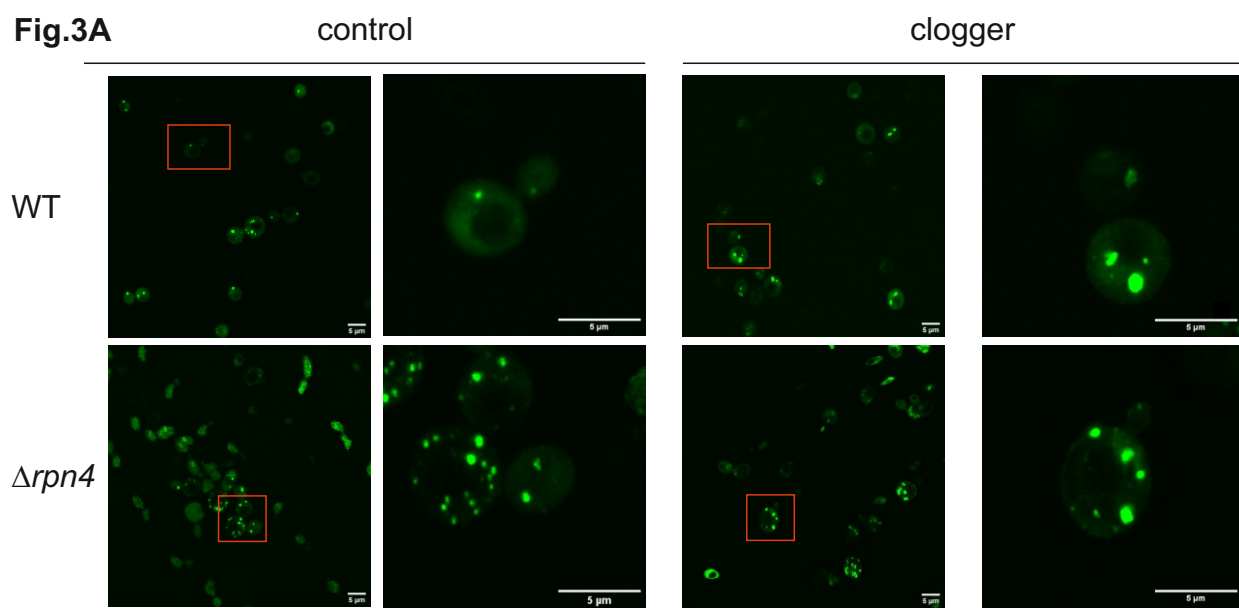

Supplement: Supplementary file 11 — Source Data for Figure 3 [file EMBJ-42-e112309-s003.zip › SourceData_Figure 3/SourceData_Figure 3A.pdf]

Fig.3B

GFP

Sod1

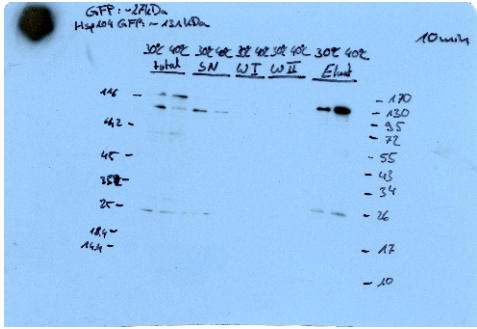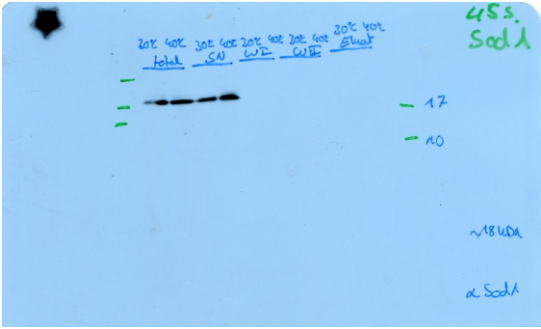

Ssa1

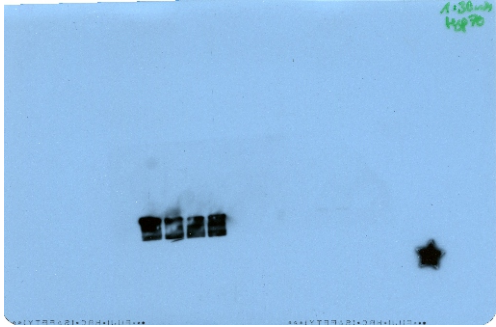

Supplement: Supplementary file 11 — Source Data for Figure 3 [file EMBJ-42-e112309-s003.zip › SourceData_Figure 3/SourceData_Figure 3B.pdf]

**Fig.5A**

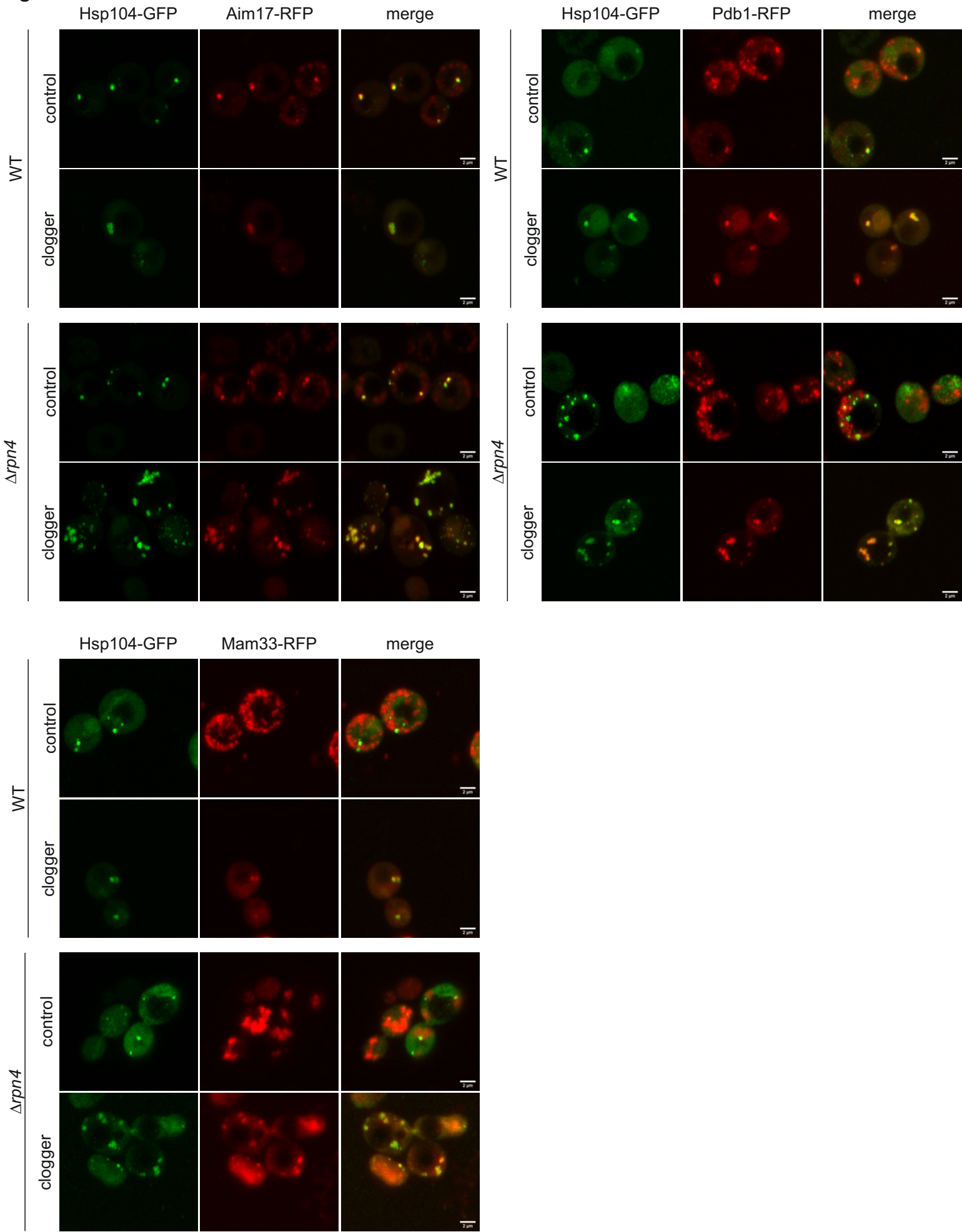

Supplement: Supplementary file 12 — Source Data for Figure 5 [file EMBJ-42-e112309-s004.zip › SourceData_Figure 5/SourceData_Figure 5A.pdf]

Fig.5B

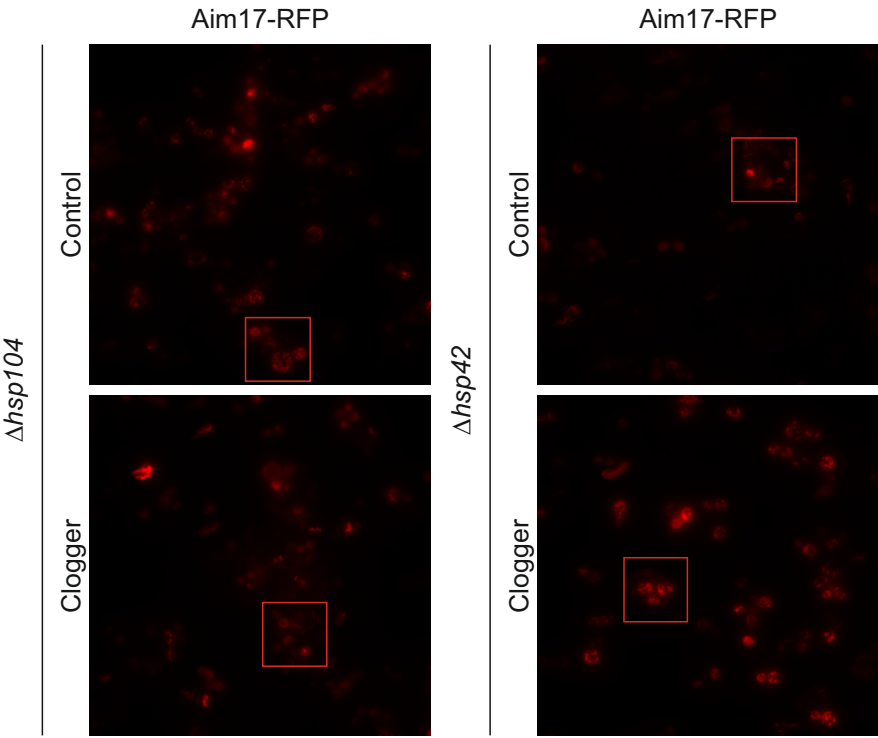

Supplement: Supplementary file 12 — Source Data for Figure 5 [file EMBJ-42-e112309-s004.zip › SourceData_Figure 5/SourceData_Figure 5B.pdf]

Fig.5C

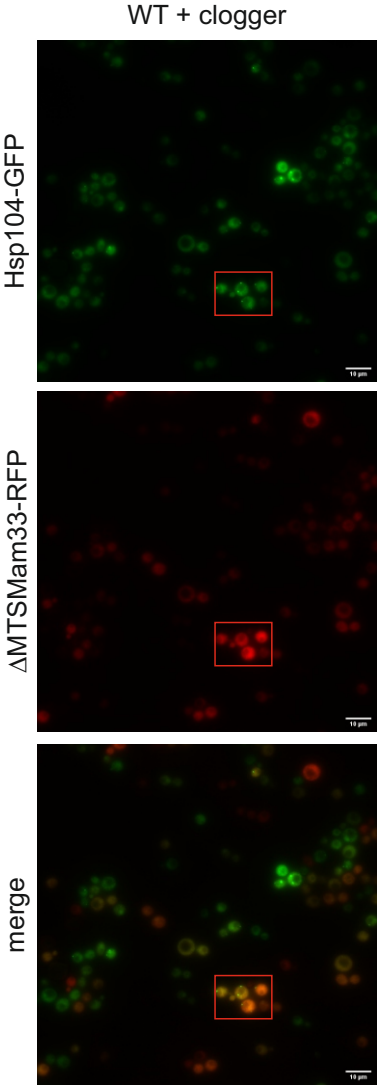

Supplement: Supplementary file 12 — Source Data for Figure 5 [file EMBJ-42-e112309-s004.zip › SourceData_Figure 5/SourceData_Figure 5C.pdf]

Fig.5D

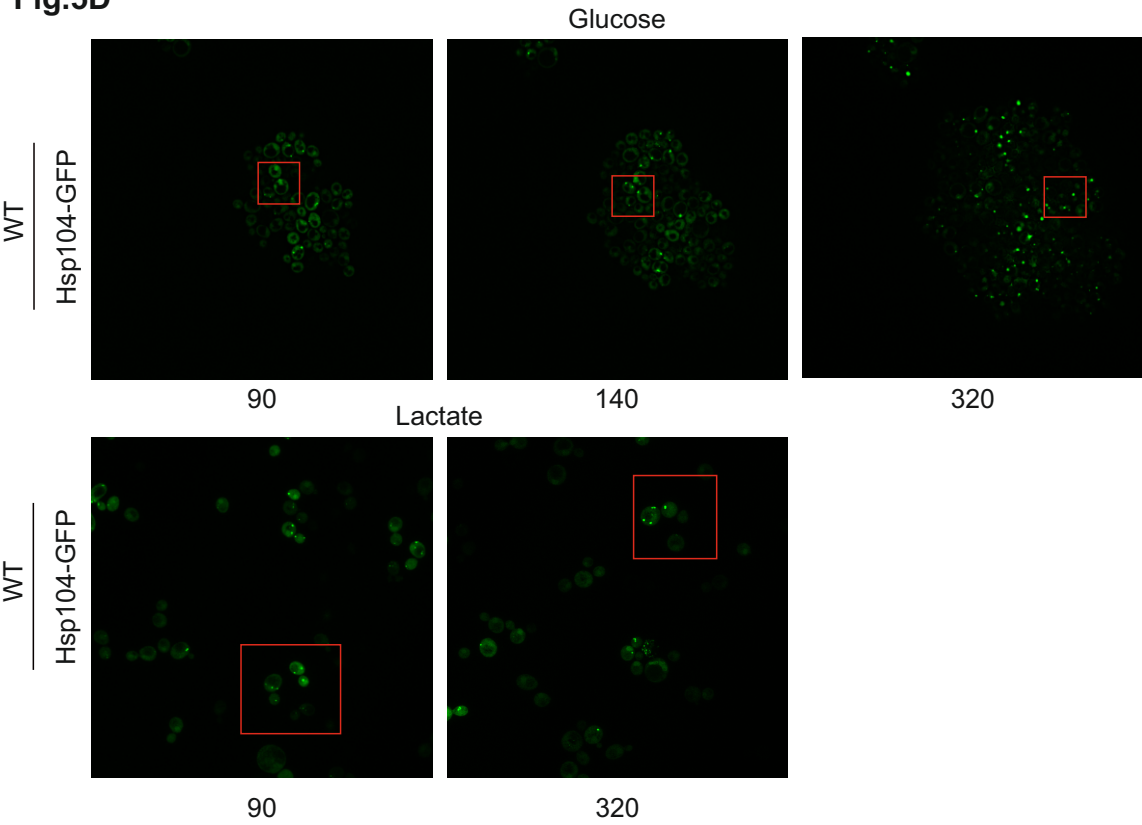

Supplement: Supplementary file 12 — Source Data for Figure 5 [file EMBJ-42-e112309-s004.zip › SourceData_Figure 5/SourceData_Figure 5D.pdf]

Fig.6A

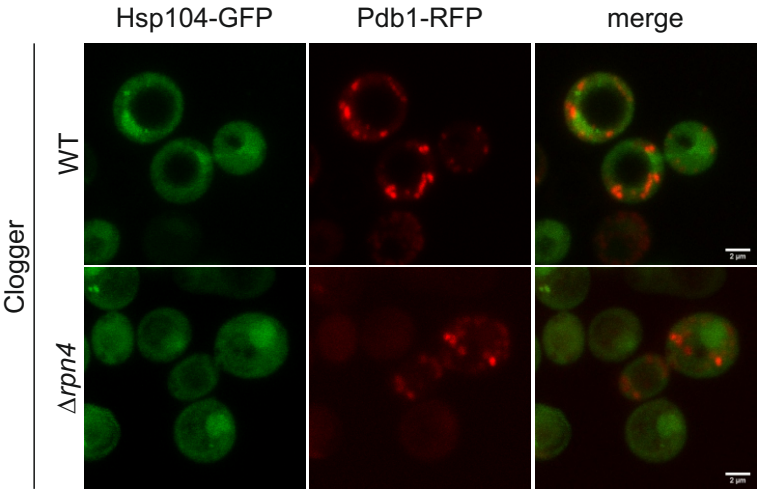

Supplement: Supplementary file 13 — Source Data for Figure 6 [file EMBJ-42-e112309-s008.zip › SourceData_Figure 6/SourceData_Figure 6A.pdf]

**Fig.6C**

$\Delta$ *rpn4* + Clogger

mito-GFP

Pdb1-RFP

merge

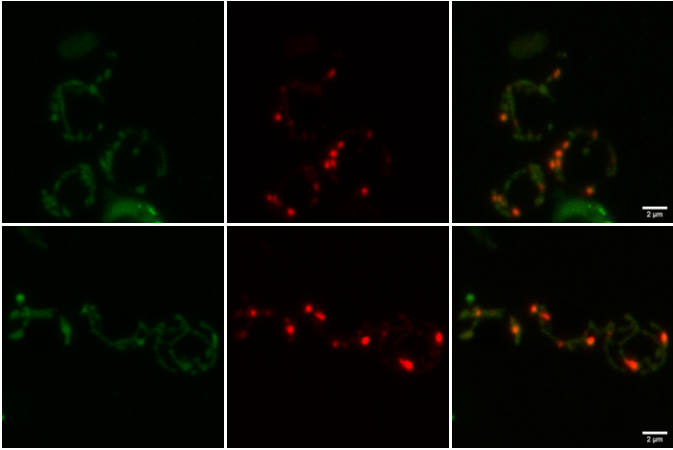

Supplement: Supplementary file 13 — Source Data for Figure 6 [file EMBJ-42-e112309-s008.zip › SourceData_Figure 6/SourceData_Figure 6C.pdf]

Fig.6D

$\Delta hsp42$

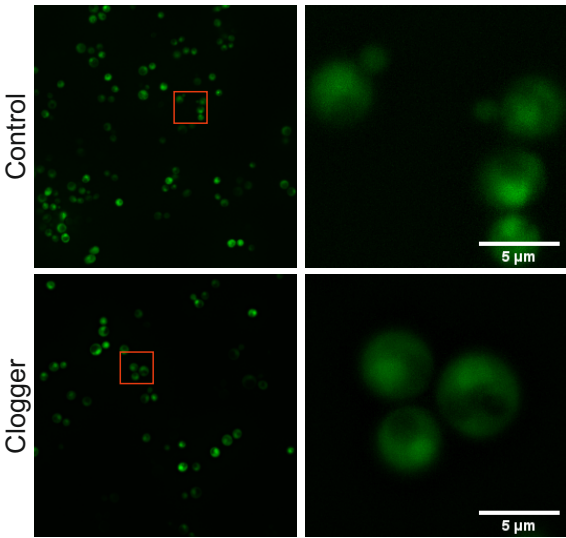

Supplement: Supplementary file 13 — Source Data for Figure 6 [file EMBJ-42-e112309-s008.zip › SourceData_Figure 6/SourceData_Figure 6D.pdf]

Fig.6F

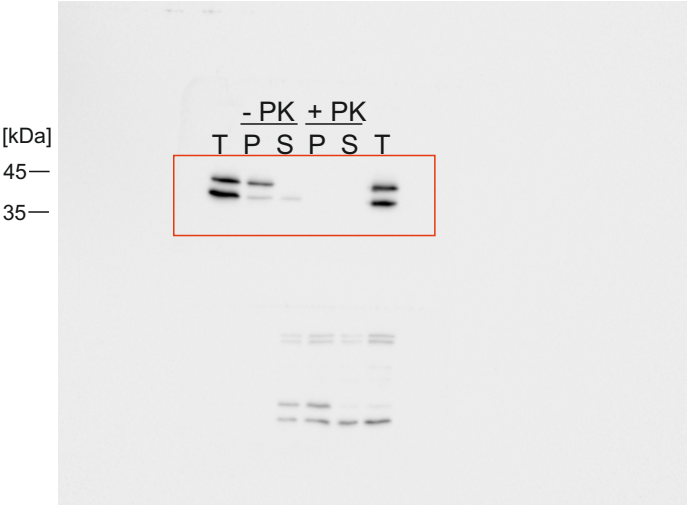

Supplement: Supplementary file 13 — Source Data for Figure 6 [file EMBJ-42-e112309-s008.zip › SourceData_Figure 6/SourceData_Figure 6F.pdf]
