# Supplementary material for: MitoStores: chaperone‐controlled protein granules store mitochondrial precursors in the cytosol
Source: EMBO J. 2023 Jan 27;42(7):e112309. doi: 10.15252/embj.2022112309 (PMC10068336; doi:10.15252/embj.2022112309)
Supplement: Supplementary file 14 [file EMBJ-42-e112309-s009.pdf]

THE EMBO JOURNAL

MitoStores: Chaperone-controlled protein granules store mitochondrial precursors in the cytosol

Lena Kraemer, Niko Dalheimer, Markus Räschle, Zuzana Storchova, Jan Pielage, Felix Boos, and Johannes Herrmann
DOI: 10.15252/embj.2022112309

Corresponding author(s): Johannes Herrmann (hannes.herrmann@biologie.uni-kl.de)

Review Timeline:

Submission Date:	5th Aug 22
Editorial Decision:	15th Sep 22
Revision Received:	28th Nov 22
Editorial Decision:	23rd Dec 22
Revision Received:	5th Jan 23
Accepted:	12th Jan 23

Editor: Stefanie Boehm

Transaction Report:

(Note: With the exception of the correction of typographical or spelling errors that could be a source of ambiguity, letters and reports are not edited. Depending on transfer agreements, referee reports obtained elsewhere may or may not be included in this compilation. Referee reports are anonymous unless the Referee chooses to sign their reports.)

Thank you again for submitting your manuscript to The EMBO Journal and sending the preliminary response to the referee comments (copied again below). In light of the overall positive referee feedback, we invite you to prepare and submit a revised manuscript.

As we discussed, a complete mechanistic experimental definition of all aspects of formation, regulation and physiological function of MitoStore granules will not be required. However, in the revised version it will nonetheless be crucial to further strengthen the main conclusions and to add to the characterization of the MitoStores, specifically addressing the referees' points regarding their dynamics, role for cell growth in the context of RPN4 deletion and effect on mitochondria. In addition, please include the experimental data on the role of Hsp42 and Hsp104 in resistance to clogger expression, as well as the effect of their simultaneous deletion. Furthermore, the proposed additional experiments to define Aim17's effect on condensate formation should be included and any potential model for its function clearly discussed as such. Please also resolve all concerns regarding necessary controls and missing experimental details. Finally, please also carefully consider all other referee comments and revise the manuscript and figures as needed, as well as providing a detailed response to each comment. Please also remember that the revised manuscript should fulfill all EMBO Journal formatting requirements when it is next submitted (please see below and: https://www.embopress.org/page/journal/14602075/authorguide#submissionofrevisions)

Referee #1:

Summary
In the manuscript by Krämer et al., the authors investigate intracellular quality control mechanisms that arise when mitochondrial protein import is impaired. The aim of the study was to uncover non-proteasomal mechanisms to deal with the toxicity arising from failed mitochondrial protein import. Using the budding yeast as a model system, the authors develop an approach to induce "clogging" of the import channel with an MTS-DHFR construct. A screen was performed on a rpn4 null background, which removes a key transcriptional regulator for the synthesis of the proteasomal subunits. This revealed sequestration of mitochondrial precursor proteins mediated by Hsp104 and Hsp42 into cytoplasmic granules that the authors call "MitoStores". The data showing the presence of granules enriched for chaperones and mitochondrial proteins is robust. That said, there are still some holes in this study with the current data to completely support the conclusions generated.

Major points

1. In Figure 1, the authors report that induction of the DHFR clogging construct results in a mild activation of the transcription factor Rpn4 and a corresponding synthesis of the Pre6 subunit of the proteasome. Whereas deletion of rpn4 reportedly results in a lower steady abundance of Pre6. However, the data in Figure 1E is quite variable on the WT background. Despite this, there still is residual Pre6 protein level that is not affected by the rpn4 deletion. Considering that the MitoStores granules are supposedly to compensate above and beyond the ubiquitin-proteasomal system upon clogging induction, it is important to demonstrate the activity of this degradation system. Simply measuring Pre6 protein level is not an adequate proxy of the ubiquitin-proteasomal system. What is the activity of ubiquitin-proteasomal system in the conditions investigated?

2. The enhanced growth rate in the rpn4 deletion strains following induction of the clogger compared to WT was very surprising. What are the possible mechanisms to account for that effect? Here, it would be important to establish the consequences on transcriptional responses of the nuclear genome and potential cytoplasmic translation regulation as compensatory mechanisms. In the rpn4 deletion background there could be translation repression in the synthesis of the mitochondrial targeted proteins or others, so the inherent proteotoxicity would be less despite the appearance of GFP positive foci following the clogger induction.

3. The authors should establish the consequences with a double deletion of rpn4 and hsp42 or hsp104.

4. How dynamic are the MitoStores and are these granules a sequestered pool that can be retargeted to mitochondria or are these proteins ultimately trapped within the granule and ultimately destined for degradation?

5. What consequence does the clogger have on the mitochondrial membrane network? Moreover, what is the mitochondrial morphology phenotype in the rpn4 deletion background?

6. The colocalization studies in Figure 6 require additional controls (e.g., a mitochondrial marker that is independent of the split GFP reporter system). As it currently stands, the spatial resolution of the imaging is poor to generate the conclusions that the authors propose.

7. The spatial distribution of the Hsp104-GFP differs dramatically in the WT background in the panels of Figure 5A. Therefore, what is a truly representative state for this factor?

Referee #2:

In their manuscript entitled "MitoStores: Chaperone-controlled protein granules store mitochondrial precursors in the cytosol", Krämer et al. reveal a new protein quality control mechanism that alleviates damaged caused by un-imported mitochondrial proteins. This mechanism, which requires the heat shock proteins Hsp42 and Hsp104, transiently stores mitochondrial precursors in cytosolic deposits which they named MitoStores. This discovery was initiated by the finding that rpn4 knockout cells do not exhibit a cell cycle arrest upon clogging mitochondrial import sites. The authors used proteomics to demonstrate that though the proteasome level is low in Δrpn4 cells, these cells contain higher levels of several heat shock proteins, including Hsp42 and Hsp104. Consistently, more Hsp104 containing aggregates (=MitoStores) were detected in Δrpn4 cells, especially when mitochondria were clogged. Interestingly, mitochondrial matrix proteins were enriched in the MitoStores, in a transient manner; when the clogger was cleared the proteins could be imported into mitochondria. The authors convincingly showed that MitoStores formation depends on Hsp42 and that they prevent degradation of mitochondrial precursors. Finally, the formation of MitoStores was shown to be necessary for recovery from stress induced by clogger expression, in Δrpn4 cells.

Although we largely understand how proteins are translocated into the mitochondria, little is known about the upstream processes that safely target proteins to these organelles. As both physiological and disease conditions are known to disrupt efficient protein translocation into mitochondria, better understanding of cytosolic quality control mechanisms that aid with targeting and prevent precursors proteotoxicity is important. This study is therefore impactful and greatly contributes to the field. Overall, this is a well-written manuscript and well-designed study, presenting convincing data with interest to the general scientific community.

Major comments:

1. The authors' model suggests that two alternative strategies reduce the proteotoxicity of un-imported mitochondrial proteins: 1. Degradation- mediated by Rpn4, 2. Sequestration by MitoStores- mediated by heat shock proteins, including Hsp104 and Hsp42. However, in wild type cells both pathways are activated leading to a RPN4 dependent cell cycle arrest. Cell proliferation and tolerance of mitochondrial clogging is greatly improved when this natural balance is disturbed, namely the degradation capacity is significantly reduced and the sequestration increases.
If MitoStores are superior to Rpn4 mediated degradation, why is this pathway activated in cells with clogged mitochondria? Is there a trade off in bypassing cell cycle arrest in Δrpn4 cells? Why is storage of un-imported proteins better than their degradation?
While these questions might be beyond the scope of this study, they are important for the model. These questions should be at least raised and hypotheses that reconcile the model should be included in the discussion session.

2. Fig 1F- It would be helpful to show that adding back RPN4 to Δrpn4 cells can suppress the growth phenotype. This will confirm that the increased proliferation of Δrpn4 cells is due to RPN4 deletion and not a background mutation.
Another option is to use a RPN4 wild type strain that is incapable of increasing proteasome levels (ΔPACE- PRE1. See PMID: 18832351), which will allow RPN4 separation of function (proteasome-induction from other transcription activities).

3. Fig S1D- It is unclear why nonfermentable carbon (lactate) was used here. Intact respiration is required for proliferation in lactate medium. Antimycin should block respiration, and indeed no growth was detected for at least one day. Is it possible that the cells started proliferating only after the Antimycin became less potent and the differences between strains are due to differences in the stability/ internalization of Antimycin?

4. Page 11- "Upon clogger induction, the RFP signal formed defined punctae that colocalized with Hsp104-GFP. Whereas Aim17-RFP binding to Hsp104-GFP was seen even without clogger induction, Pdb1 and Mam33 were only found in cytosolic granules when import was slowed down by clogger expression. Thus, the association of individual proteins with cytosolic MitoStores likely depends on the individual protein and the prevailing cellular conditions."
Protein tags, especially large tags such as RFP, can slowdown the import of proteins into mitochondria. The impact of RFP can vary depending on the protein. Indeed, in Aim17-RFP expressing cells, HSP104-GFP seems mostly in punctae and less in the cytosol.
Do the MS data support the difference stated above between the un-tagged Aim17, Pdb1, and Mam33? If yes, it would be helpful to state it. Otherwise, the statement above should be removed from the manuscript.

5. Page 11- "It should be noted that Hsp104-GFP-containing granules were frequently found in yeast cells even in the absence of the clogger or uncouplers as long as cells were grown on nonfermentable carbon sources which induce the expression of mitochondrial proteins (Fig. 5C)."
A control of a fermentable carbon source media is missing, as well as quantification. Are the Hsp104-GFP-containing granules, formed in nonfermentable carbon, similar to the clogger induced granules? Do they contain mitochondrial proteins? Are they localized in proximity to mitochondria? Such details could strengthen the statement above.

6. Fig 6B- The authors should exclude the possibility that the split reconstitution occurs in the cytosol rather than in mitochondria- it is possible that Oxa1-GFP1-10 also accumulates in the cytosol.

7. Fig 6B- Addition of the Δrpn4/Δhsp42 strain to this experiment could provide more evidence for MitoStores role in recovering the mitochondrial proteome following clogging (this point is further elaborated in comment 9).

8. Fig 6G- the conclusion could be strengthened by the addition of controls: I. Cytosolic DHFR control in all backgrounds. II. Δhsp42 and Δhsp104 alone (will the recovery of these be worse than wt?).
Please specify the whether the yeast were plated on nonfermentable or fermentable carbon source in the figure legend.

9. Fig 1F and 6G - The assays used in these figures are different and might indicate different phenotypes. In 1F growth curves were assessed during clogger expression= growth in stress conditions. Fig 6G on the other hand measure growth recovery following stress. As the impact of Hsp42/104 on recovery is convincing, it would be informative to examine whether these Hsps are crucial for bypassing the cell cycle arrest during stress.

10. What causes cell cycle arrest of clogger expressing cells, mitochondrial dysfunction or cytosolic proteotoxicity?
Fig 6 indirectly suggests a potential role for Hsp42/104 in preserving the mitochondrial proteome during and following stress, by stabilization and transient storage of precursors (Page 12- "Interestingly, in the absence of Rpn4, considerably higher levels were imported into the mitochondria indicating that Aim17 is indeed stabilized in the cytosol in an import-competent fashion"). This raises the possibility that the high proliferation rate of Δrpn4+clogger cells is a result of healthier mitochondria (particularly as the assay is done in nonfermentable conditions). However, this possibility is neither directly addressed experimentally nor discussed as a potential outcome and leaves the redear somewhat confused about the purpose of including these data.
In addition, nonfermentable medium, which requires respiration, was used throughout the manuscript. Will Rpn6 and Hsp42 have the same impact in fermentable conditions? Clearly the clogger expression system is limiting and excludes the use of fermentable medium (Fig 1F), however the recovery (Fig 6G) could be tested in fermentable media.
This might be beyond the scope of the manuscript but should be at least discussed.

11. The conclusion from figure 6G could be greatly strengthen by showing that the growth defect of wt cells could be reduced by overexpression of Hsp42/Hsp104/Hsp26. Alternatively, assuming that Hsp42/Hsp104/Hsp26 are regulated at the transcription level, their expression could be "fixed" in wt and Δrpn4 cells by switching their promoter (pADH for example). This will render Hsp42/Hsp104/Hsp26 levels unresponsive to the presence of RPN4 and equalize the growth rates of wt and Δrpn4 cell with clogged mitochondria.
It is possible that changing the levels of these 3 proteins will not be sufficient but this will be informative too. In such case a preconditional heat shock could be used prior to clogger expression to examine whether it will help tolerate the stress.

Minor comments:
1. Is it possible that in rpn4 del the clogger is sequestered in aggregates and is thus less accessible to clog mitochondria? Can this possibility be excluded by the MS data? It will be helpful to comment about the levels of Cyb2 in Hsp104 aggregates, in wt versus rpn4 del.
2. Fig 2G- what control is used here? Please add this detail.
3. Fig. S3A-F- luciferaseR188Q,R261Q and Ubc9Y68L are referred to as Lucts Ubc9ts in this figure. Please change to have consistent terminology.
4. "In glucose-grown wild type cells, Hsp104-GFP was evenly distributed throughout the cells whereas upon growth in non-fermenting lactate-based media cells showed a small number of aggregates (4 on average) (Fig. 3A)." Glucose conditions are not shown in the figure. Either add it to the figure or adjust the text.
5. Table 2 is not referred to in the main text. It would be helpful to add it when relevant
6. Fig 6D- Hsp104-GFP is not mentioned in the figure, only in the legend. Please add.
7. "This precursor was protease accessible and found in high-speed pellets after solubilizing mitochondrial membranes with detergent, indicating that MitoStores remain associated with the mitochondrial surface even during cellular fractionation procedures (Fig. 6E)" Typo- should be Fig 6F.

8. "Since Hsp42 was crucial for the formation of MitoStores (Fig. 6D) we tested whether deletion of Hsp42 suppressed the clogger resistance observed in Δrpn4 mutants (Fig. 6F)."
Typo - Fig. 6F is incorrect. Is it Fig 1F?
9. Fig S5E- legend mentions 30 and 37 degrees but it seems as only one of these temperatures was used?
10. Typo in the discussion session: "Hsp42 and Hsp104 are upregulated in Drpn4 cells even before clogger expression explaining the increased resistance against mitoprotein-induced stress of these cells." Drpn4 should be rpn4 deletion
11. Typo Figure 6: "(F) The mitochondria used for D were treated with proteinase K (PK) to remove surface-exposed proteins." For E not for D
12. Fig 6F- why is the mature band sensitive to ProK?

Referee #3:

In this study the authors investigated how yeast cells respond to mitoprotein-induced stress triggered by clogger proteins. Consistent with their previous studies, they showed that the transcription factor Rpn4 upregulates proteasomes, which degrades mitochondrial precursors, and this leads to growth arrest in clogger expressing cells. They showed that in the absence of Rpn4, cells fail to activate proteasomal degradation. These cells are surprisingly resistant to clogger-induced growth arrest, and upregulate chaperone proteins Hsp104 and Hsp42. Using proteomics and microscopy, the authors found that a subset of non-imported mitochondrial proteins, primarily matrix proteins that contain an MTS, are transiently targeted to Hsp104/Hsp42-regulated cytosolic granules, named as "MitoStores" by the authors. These proteins are re-imported into mitochondria after removal of clogger induction, and this potentially protects against the toxic accumulation of mitochondrial precursors in Δrpn4 cells. Collectively, these results suggest that upon clogger-induced mitochondrial import stress, Hsp104/Hsp42-induced MitoStores act as a second line of defense in addition to Rpn4-dependent proteasomal degradation to safeguard against mitoprotein-induced stress.

Overall, the question of how cells regulate nuclear-encoded mitochondrial precursors under mitochondrial import stress is an important one. This paper provides a step forward in our understanding of how the cytosol regulates mitochondrial precursors during stress. The authors used proteomics to clearly reveal the interactomes of Hsp104 in wild-type and Δrpn4 cells with or without expression of mitochondrial cloggers. The data is clear and solid. Some issues the authors should address prior to publication, especially with regard to whether mitostores are different than previously described protein aggregates, are listed below.

1. The authors named the Hsp104-containing granules in clogger-expressing cells or in cells cultured in non-fermentable carbon sources as "MitoStores", because these granules contain numerous mitochondrial proteins. However, this name suggests that these granules have specificity in incorporating mitochondrial proteins, but not proteins destined for the cytoplasm or other organelles. As shown in Fig. S4, proteins of the secretory pathways also interact with Hsp104 in Δrpn4 cells. Are they localized to the MitoStores as well? As the authors indicate, these structures are very similar to those identified previously as CytoQ or INQ (or JUNQ) compartments. If the authors would express a known CytoQ substrate (some they use in the paper like Ubc9 mutants or Q97-GFP, etc) in the presence of clogger, would this protein localize in the same Hsp104 puncta as mitochondrial proteins? I guess what I am getting at here is that while it's clear mitochondrial proteins can localize to Hsp104 protein aggregates when their import into mitochondria is impaired (and proteasome is inhibited), this is similar to what has been shown for other misfolded or mistargeted proteins that localize to other cellular destinations. It may not be that these aggregates are special for mitochondrial proteins, but rather, mito proteins may just represent the major class of misfolded substrates in the cell when clogger is expressed. Perhaps a good way to test the specificity of these structures is to determine whether known non-mitochondrial cytoQ substrates colocalize with mitochondrial proteins in clogger expressing cells. Things like CPY* lacking its signal sequence, or the substrates the authors use here including Ubc9 mutants or Q97-GFP. If these cytoQ substrates co-localize with mitochondrial proteins and Hsp104 when clogger is expressed, then I would recommend not using the term mitostores but rather call the structures cytoQ or just protein aggregates. Giving them the name mitostores implies that they are something different than previously described proteins aggregate structures, and this may not necessarily be the case.

2. The authors showed that MTS-containing mitochondrial matrix proteins are more likely to be targeted to the MitoStores. It would be important to know whether MitoStore-targeting is highly dependent on the MTSs. For example, does removing the MTSs from MitoStore-enriched mitochondrial proteins prevent their interaction with Hsp104, and also their localizations to the MitoStores? Also, for proteins like mitochondrial carriers that did not show up in the Hsp104 pull downs, it may be worthwhile to examine their colocalization with Hsp104 in clogger cells just in case their lack of identification in Hsp104 foci was due to a technical limitation because they are highly hydrophobic in nature.

3. Through clear proteomics data, the authors showed that the MitoStore its regulated by interaction between its clients and Hsp104. They also showed used genetics and microscopy to show the formation of these granules are Hsp42-dependent. As a result, they described MitoStores as Hsp104/Hsp42-regulated. However, functional differentiation between Hsp104 and Hsp42 will be very helpful in understanding the formation of these granules. For example, whether Δhsp104 cells are able to form MitoStores, especially given that Δrpn4 Δhsp104 mutant cells showed similar growth defects as in MitoStore-deficient Δrpn4 Δhsp42 mutant cells? Additionally, can the authors comment on why loss of both Rpn4 and Hsp42 or 104 does not lead to more

toxicity in WT cells?

Minor points that should be addressed:
1. In Fig. 3C, the lower right Mass Spectrometry result, label of x-axis, right bracket after DHFR-GFP is missing.
2. The label of y-axis of the bar graph in Fig. 6G is missing. Something like "OD600" or "Growth/Survival" will be helpful.
3. It would be clearer if the proximity of MitoStores to mitochondria is shown in the Fig. 6H.
4. Please correct the figure legend for Fig. S2. To me, it looks like the respiratory proteins are indicated in purple, not blue.
5. "Drpn4" in the first paragraph of discussion should be "Δrpn4".
6. It would be helpful to quantify and compare numbers/sizes of Hsp104 granules in Glucose- versus Lactate- containing media in Figure 5C.

Manuscript Number: EMBOJ-2022-112309R
MitoStores: Chaperone-controlled protein granules store mitochondrial precursors in the cytosol

We would like to thank all three reviewers for the thorough evaluation of our manuscript. We were very pleased to see that all three referees in general were positive and supported our study about the storage of mitochondrial precursor proteins in cytosolic MitoStore granules. We addressed the critical points that were raised by the reviewers by a the addition of new data (Figs 2H, 2I, 5B, 5C, 5D, EV1E, EV1F, EV1G, EV5E, and EV5F) and by changes of the text.

In particular, we followed the suggestion of all three referees

- to test whether the presence of a mitochondrial targeting sequence is necessary for the inclusion of substrates into MitoStore granules (novel Fig. 5C),
- to elucidate in more depth the relevance of Hsp42 (and Hsp104) in the protection against mitoprotein-induced stress, including data from respiring cells that were not challenged by clogger expression (novel Figs. 2H, 2I, 5B, EV1E, EV5E and EV5F),
- to test whether overexpression of Hsp42 and Hsp104 in wild type cells provides increased resistance to clogger expression (novel Fig. 2H), and
- to add data on the impact of MitoStores on mitochondrial morphology and function (novel Figs. 2I, 5D, EV1G, and EV5E).

With the inclusion of the new additional data and alterations to the text as proposed by the reviewers, we hope that we were able to satisfactorily address all points raised by the referees.

Point-by-point response to the comments of the referees

Reviewer #1:

We thank the referee for her/his very positive evaluation and for the comment that ‘the data showing the presence of granules enriched for chaperones and mitochondrial proteins is robust.’ We addressed the specific and helpful comments as described in the following:

Major points
1. In Figure 1, the authors report that induction of the DHFR clogging construct results in a mild activation of the transcription factor Rpn4 and a corresponding synthesis of the Pre6 subunit of the proteasome. Whereas deletion of rpn4 reportedly results in a lower steady abundance of Pre6. However, the data in Figure 1E is quite variable on the WT background. Despite this, there still is residual Pre6 protein level that is not affected by the rpn4 deletion. Considering that the MitoStores granules are supposedly to compensate above and beyond the ubiquitin-proteasomal system upon clogging induction, it is important to demonstrate the activity of this degradation system. Simply measuring Pre6 protein level is not an adequate proxy of the ubiquitin-proteasomal system. What is the activity of ubiquitin-proteasomal system in the conditions investigated?

The relevance of Rpn4 for the degradation of mitochondrial precursor proteins in the cytosol is demonstrated in Fig. EV1A. This Western blot shows that *Δrpn4* cells accumulate increased amounts of precursor proteins (such as Mdj1 and Rip1) upon clogger expression (see for example the 60 min time point), confirming their reduced degradation in cells lacking Rpn4. This supports our hypothesis of a reduced proteasomal degradation of mitochondrial precursor proteins that are of relevance for our study.
In addition, the Rpn4-driven expression of proteasomal subunits was studied in detail and resulted in almost 100 publications. This analysis was pioneered by the laboratories of Horst Feldmann (Munich), Alex Varshavsky (Pasadena), Youming Xie (Detroit), Vadim Karpov (Moscow) and Jürgen Dohmen (Cologne) but also other labs contributed to elucidate the regulatory circuit by which proteasome-dependent degradation of the transcription factor Rpn4 controls proteasome activity in yeast. In the absence of Rpn4, basal levels of proteasome complexes and proteasome activity are present. As a consequence, *Δrpn4* cells grow as well as wild type at physiological conditions (30°C, see Fig. 1F) and express proteasome subunits, albeit at somewhat reduced levels (e.g. Pre6, see Fig. 1E and EV1B). However, at an elevated temperature (37°C) or on non-fermentable carbon sources, Rpn4 becomes important for optimal growth (see Fig. EV3A, B). The levels of proteasome activity were measured before in many studies either in vitro by assays with fluorescent peptides (PMID: 30629175; 32821821; 33143019) or in vivo by following the degradation of proteasome substrates in Western blots (e.g. PMID: 11248031; 19933873; 22349505; 25157437). Since all studies came to consistent conclusions (which are described in our introduction), we decided not to repeat these measurements of the proteasome activity in wild type and *Δrpn4* cells again.

2. The enhanced growth rate in the rpn4 deletion strains following induction of the clogger compared to WT was very surprising. What are the possible mechanisms to account for that effect? Here, it would be important to establish the consequences on transcriptional responses of the nuclear genome and potential cytoplasmic translation regulation as compensatory mechanisms.

The clogger resistance of the *Δrpn4* strain depends on the presence of Hsp42 and Hsp104 (Fig. 5B, 6D). We expect that the increased resistance is due to the fact that both proteins are expressed at elevated levels in the *Δrpn4* strain even before the onset of clogger expression. Thus, the *Δrpn4* mutant is already preconditioned owing to its inherent Hsp42-Hsp104 induction. In order to support this hypothesis, we now added a novel experiment with strains which constitutively express elevated Hsp42 and Hsp104 levels (novel Fig. 2H). Constistant with our hypothesis, the ectopic expression of just these two proteins increases the growth in the presence of the clogger (and also under respiring conditions). On the contrary, deletion of Hsp104 and Hsp42 not only makes *Δrpn4* cells hypersensitive to clogger expression (Fig. 6G), but prevents growth on non-fermentable carbon sources completely (novel Fig. 2I). Thus, in order to respire, cells either need to upregulate the proteasome (via Rpn4) or employ Hsp42 and Hsp104 for MitoStore formation.

In the rpn4 deletion background there could be translation repression in the synthesis of the mitochondrial targeted proteins or others, so the inherent proteotoxicity would be less despite the appearance of GFP positive foci following the clogger induction.

Regarding the levels of clogger and mitochondrial precursors in wild type and *Δrpn4* cells: We show the levels of clogger in Fig. 2A, for which the precursor levels are much higher in *Δrpn4* mutants than in wild type. Moreover, we show the levels of precursors of other mitochondrial proteins (Mdj1, Rip1) in Fig. EV1A, again showing higher levels in the *Δrpn4* mutant than in wild type. These data show that the increased resistance of the *Δrpn4* cells is not caused by a reduced expression of mitochondrial proteins.

However, following the suggestion of the referee and to exclude the repression of protein synthesis, we now monitored translation rates by incubating cells in the presence of ^{35}S-methionine. As shown in the novel Fig. EV1F, the presence or absence of Rpn4 did not alter protein synthesis under our cultivation conditions.

3. The authors should establish the consequences with a double deletion of rpn4 and hsp42 or hsp104.

We now followed the suggestion of the referee. As shown in the novel Fig. 2I, mutants lacking both Rpn4 and Hsp42/Hsp104 are basically unable to grow on non-fermentable carbon sources, pointing to severe defects in mitochondrial biogenesis. Of note, these experiments were carried out without expression of the clogger and thus under physiological growth conditions. Thus, if cells cannot increase the levels of the ubiquitin-proteasome system, the ability to form MitoStores is crucial for mitochondrial biogenesis. We thank the referee for suggesting this interesting experiment.

4. How dynamic are the MitoStores and are these granules a sequestered pool that can be retargeted to mitochondria or are these proteins ultimately trapped within the granule and ultimately destined for degradation?

MitoStores are highly dynamic and rapidly disappear when clogger expression is repressed (Fig. 6A). Thereby Hsp104 gets dispersed in the cytosol and mitochondrial precursor proteins are chased into mitochondria (Fig. 6A, B, C). Thus, mitochondrial proteins can be released from MitoStores and imported. This re-distribution of mitochondrial proteins is found in wild type and in *Δrpn4* cells and thus not just the consequence of the reduced proteasomal activity of *Δrpn4* cells.

5. What consequence does the clogger have on the mitochondrial membrane network? Moreover, what is the mitochondrial morphology phenotype in the rpn4 deletion background?

We did not find apparent changes of the mitochondrial morphology in *Δrpn4* cells (novel Fig. EV1G). Prolonged expression of the clogger disturbs mitochondrial morphology in wild type cells (PMID: 30886345), and to a lesser degree in *Δrpn4* cells (novel Fig. EV5E). In the absence of clogger expression, *Δrpn4* cells show a wild type-like mitochondrial network (novel Fig. EV5E).

6. The colocalization studies in Figure 6 require additional controls (e.g., a mitochondrial marker that is independent of the split GFP reporter system). As it currently stands, the spatial resolution of the imaging is poor to generate the conclusions that the authors propose.

We agree with the referee that it is often difficult to distinguish signals from proteins on mitochondria from those in mitochondria. This is why we used the split-GFP system (Fig. 6B) in order to make sure that clients of the MitoStores were chased into mitochondria rather than just onto their surface.

7. The spatial distribution of the Hsp104-GFP differs dramatically in the WT background in the panels of Figure 5A. Therefore, what is a truly representative state for this factor?

Expression of Aim17-RFP induced the formation of condensates that are bound by Hsp104-GFP even under conditions were no clogger is expressed. Aim17 is a major constituent of the MitoStores and apparently a ‘problematic’ protein. We will study the behavior of this interesting protein in the future. We already excluded that Aim17 is essential for MitoStore formation. We show this here for inspection by the referee as Figure 1. We also improved the text to better explain the differences in the Hsp104-GFP patterns in the different strains.

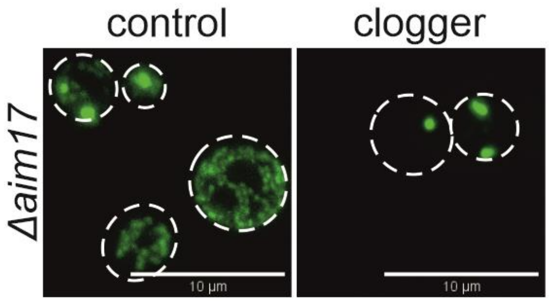

Fig. 1: **MitoStore formation does not depend on Aim17.** Hsp104-GFP was visualized in Aim17-deficient cells. Upon expression of the clogger, Hsp104-GFP showed the characteristic punctate distribution, indicating that Aim17 is not essential for the formation

Reviewer #2:

We thank the referee for the very positive evaluation and for her/his statement that ‘this study is therefore impactful and greatly contributes to the field. Overall, this is a well-written manuscript and well-designed study, presenting convincing data with interest to the general scientific community.’ We addressed her/his comments as follows:

Major comments:

1. The authors' model suggests that two alternative strategies reduce the proteotoxicity of un-imported mitochondrial proteins: 1. Degradation- mediated by Rpn4, 2. Sequestration by MitoStores- mediated by heat shock proteins, including Hsp104 and Hsp42. However, in wild type cells both pathways are activated leading to a RPN4 dependent cell cycle arrest. Cell proliferation and tolerance of mitochondrial clogging is greatly improved when this natural balance is disturbed, namely the degradation capacity is significantly reduced and the sequestration increases.

If MitoStores are superior to Rpn4 mediated degradation, why is this pathway activated in cells with clogged mitochondria? Is there a trade off in bypassing cell cycle arrest in Δrpn4 cells? Why is storage of un-imported proteins better than their degradation?

While these questions might be beyond the scope of this study, they are important for the model. These questions should be at least raised and hypotheses that reconcile the model should be included in the discussion session.

We fully agree with the referee that is a major question of our study: Why are *Δrpn4* cells more resistant? We assume that this increased resistance is caused by the constitutively increased Hsp42 and Hsp104 levels of this strain. Thus, this strain shows a kind of preconditioning similar to that of Susan Lindquist's famous temperature preconditioning experiment for 'induced thermotolerance' for which yeast cells were stepwise exposed via 37°C to 50°C (Borkovich et al. 1989. MCB 9, 3919f; Lindquist and Craig, 1988). The relevance of Hsp104 for this increased thermotolerance was shown already 30 years ago, long before its molecular function was unraveled (Sanches et al. 1992. EMBO J. 11, 2357f). In order to confirm that the increased Hsp40/Hsp104 levels are sufficient for the increased resistance, we now added an experiment for which we constitutively overexpressed Hsp40 and Hsp104 in wild type cells. In consistence with our hypothesis, this strain also shows hyperresistance to clogger expression. We added the data of this new experiment as novel Fig. 2H and added text in the Results and Discussion sections. Moreover, we added a novel experiment in which Hsp42/Hsp104 were deleted from *Δrpn4* cells (novel Fig. 2I). This strain grows well on glucose but is unable to grow on non-fermentable carbon sources, again supporting our conclusion that Rpn4-dependent upregulation of the UPS and Hsp42/Hsp104-mediated storage of precursors are two alternative mechanisms to protect cells from precursor-induced toxicity. See also answer to question 2 of referee #1.

2. Fig 1F- It would be helpful to show that adding back RPN4 to Δrpn4 cells can suppress the growth phenotype. This will confirm that the increased proliferation of Δrpn4 cells is due to RPN4 deletion and not a background mutation.
Another option is to use a RPN4 wild type strain that is incapable of increasing proteasome levels (ΔPACE- PRE1. See PMID: 18832351), which will allow RPN4 separation of function (proteasome-induction from other transcription activities).

We added the requested control which shows that an Rpn4-expressing plasmids renders the *Δrpn4* strain again sensitive to clogger expression. This novel experiment is now shown as Fig. EV1E.

3. Fig S1D- It is unclear why nonfermentable carbon (lactate) was used here. Intact respiration is required for proliferation in lactate medium. Antimycin should block respiration, and indeed no growth was detected for at least one day. Is it possible that the cells started proliferating only after the Antimycin became less potent and the differences between strains are due to differences in the stability/ internalization of Antimycin?

Antimycin inhibits the membrane potential so that growth on lactate is inhibited. Over time, antimycin becomes less potent and cells start growing despite the very low membrane potential. Thus, this experiment is similar to the one in which clogger is expressed. The low membrane potential of the antimycin-treated cells counteracts protein import. The *Δrpn4* mutant can better escape the inhibited growth than wild type cells, similar to what we observed for the clogger-induced inhibition. Since this experiment is

more difficult to interpret than the one with the clogger expression, we show it in the supplement. In order to better explain this experiment, we added further text to the legend of Fig. EV1.

4. Page 11- "Upon clogger induction, the RFP signal formed defined punctae that colocalized with Hsp104-GFP. Whereas Aim17-RFP binding to Hsp104-GFP was seen even without clogger induction, Pdb1 and Mam33 were only found in cytosolic granules when import was slowed down by clogger expression. Thus, the association of individual proteins with cytosolic MitoStores likely depends on the individual protein and the prevailing cellular conditions."
Protein tags, especially large tags such as RFP, can slowdown the import of proteins into mitochondria. The impact of RFP can vary depending on the protein. Indeed, in Aim17-RFP expressing cells, HSP104-GFP seems mostly in punctae and less in the cytosol.
Do the MS data support the difference stated above between the un-tagged Aim17, Pdb1, and Mam33? If yes, it would be helpful to state it. Otherwise, the statement above should be removed from the manuscript.
Please see the comment to point 7 of referee #1: Expression of Aim17-RFP induced the formation of condensates that are bound by Hsp104-GFP even under conditions were no clogger is expressed. Aim17 is a prominent constituent of the MitoStores and apparently a 'problematic' protein. We will study the behavior of this interesting protein in the future. We already excluded that Aim17 is essential for MitoStore formation. See also answer to question 6 of referee #1. We also improved the text to better explain the differences in the Hsp104-GFP patterns in the different strains.
The strong accumulation of Aim17 in MitoStores was also found in the proteomic analysis where Aim17 was not tagged. Also, Mam33 and Pdb1 were highly enriched in MitoStores. This indicates that their accumulation in the cytosol is not caused by a GFP-mediated mislocalization of these proteins.

5. Page 11- "It should be noted that Hsp104-GFP-containing granules were frequently found in yeast cells even in the absence of the clogger or uncouplers as long as cells were grown on nonfermentable carbon sources which induce the expression of mitochondrial proteins (Fig. 5C)."
A control of a fermentable carbon source media is missing, as well as quantification. Are the Hsp104-GFP-containing granules, formed in nonfermentable carbon, similar to the clogger induced granules? Do they contain mitochondrial proteins? Are they localized in proximity to mitochondria? Such details could strengthen the statement above.
We added this information as requested as novel Fig. 5D. Hsp104-GFP showed a dispersed distribution in glucose-grown cells during log phase. However, in stationary cultures, many cells show a punctate Hsp104-GFP distribution. On lactate medium, most cells contain Hsp104-GFP-bound granules even at log phase. The added figure shows still frames from life cell imaging. The corresponding movie is now also added as EV-data.

6. Fig 6B- The authors should exclude the possibility that the split reconstitution occurs in the cytosol rather than in mitochondria- it is possible that Oxa1-GFP1-10 also accumulates in the cytosol.

We verified that the split GFP signal reflects a mitochondrial distribution. In Figure 2 we show this control for inspection by the referee.

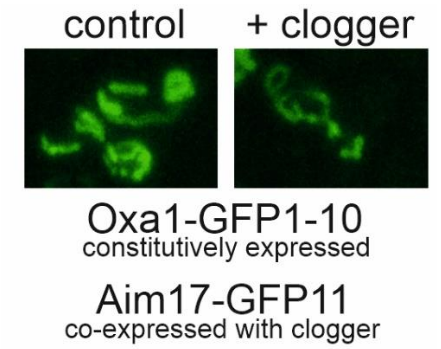

Fig. 2. The Oxa1-Aim17 split GFP pair shows a characteristic mitochondrial staining in cells. Microscopy pictures of cells used for the analysis shown in Fig. 6B of our study. For this assay, Oxa1-GFP1-10 was constitutively expressed and Aim17-GFP11 was co-expressed with clogger from a GAL promoter. The cells used here are *Δrpn4* cells. The pattern shows the distribution characteristic for mitochondria and not a general cytosolic staining. Thus, the two proteins contact each other at least predominantly in mitochondria.

7. Fig 6B- Addition of the Δrpn4/Δhsp42 strain to this experiment could provide more evidence for MitoStores role in recovering the mitochondrial proteome following clogging (this point is further elaborated in comment 9).

As now shown in the novel Fig. 5B, *Δhsp42* and *Δhsp104* cells do not form MitoStores. The additional deletion of Rpn4 in the *Δrpn4*/*Δhsp42* strain leads to cells which grow on non-fermentable carbon sources such as lactate (see novel Fig. 2I). In our view, a direct comparison of these mutants with the split-GFP reporter will be difficult. We therefore plan to explore the mitochondrial proteomes of these different combination mutants at different growth conditions in more depth in the future. This certainly requires a thorough analysis which, in our view, is beyond the immediate scope of this study.

8. Fig 6G- the conclusion could be strengthened by the addition of controls: I. Cytosolic DHFR control in all backgrounds. II. Δhsp42 and Δhsp104 alone (will the recovery of these be worse than wt?).
Please specify the whether the yeast were plated on nonfermentable or fermentable carbon source in the figure legend.

Expression of cytosolic DHFR did not reduce the number of viable colonies (see also Fig. 1B). The single *Δhsp42* and *Δhsp104* mutants behaved similar to wild type with respect to their sensitivity to clogger expression and growth phenotype. In order not to overload the figure, we decided not to add these controls. However, we added survival experiments with the single mutants below for inspection by the referee as Figure 3.

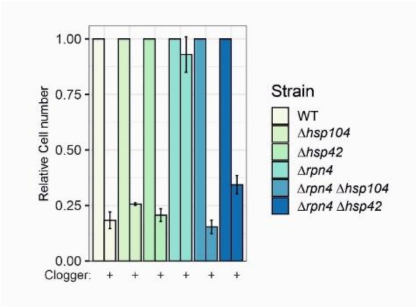

Fig. 3. Deletion of Hsp104 or Hsp42 alone leads to a wild type-like sensitivity to clogger. To measure the toxicity of clogger expression, clogger proteins were induced for 24 h in the strains indicated. Aliquots were removed and the number of viable cells were assessed by a plating assay.

Since we used the plating assay to determine the number of viable cells, we used glucose-containing plates for this experiment. This is now explicitly described in the legend.

9. Fig 1F and 6G - The assays used in these figures are different and might indicate different phenotypes. In 1F growth curves were assessed during clogger expression= growth in stress conditions. Fig 6G on the other hand measure growth recovery following stress. As the impact of Hsp42/104 on recovery is convincing, it would be informative to examine whether these Hsps are crucial for bypassing the cell cycle arrest during stress.

We generated and analyzed the *Δrpn4 Δhsp42 Δhsp104* triple mutant as suggested. This strain grows fine on glucose but is unable to grow on non-fermentable carbon sources (see novel Fig. 2I). Therefore, a direct comparison of this strain to the cell cycle measurements shown in Fig. 1C was not possible, for which cells were shifted from lactate to lactate-plus-galactose medium. Since the mutant is growing really slow on lactate, its cell cycle may be arrested. As a consequence of the bad growth behavior, it is very hard to measure the influence of the clogger for this strain.

10. What causes cell cycle arrest of clogger expressing cells, mitochondrial dysfunction or cytosolic proteotoxicity?

Fig 6 indirectly suggests a potential role for Hsp42/104 in preserving the mitochondrial proteome during and following stress, by stabilization and transient storage of precursors (Page 12- "Interestingly, in the absence of Rpn4, considerably higher levels were imported into the mitochondria indicating that Aim17 is indeed stabilized in the cytosol in an import-competent fashion"). This raises the possibility that the high proliferation rate of Δrpn4+clogger cells is a result of healthier mitochondria (particularly as the assay is done in nonfermentable conditions). However, this possibility is neither directly addressed experimentally nor discussed as a potential outcome and leaves the redear somewhat confused about the purpose of including these data.

In addition, nonfermentable medium, which requires respiration, was used throughout the manuscript. Will Rpn6 and Hsp42 have the same impact in fermentable conditions? Clearly the clogger expression system is limiting and excludes the use of fermentable medium (Fig 1F), however the recovery (Fig 6G) could be tested in fermentable media.

This might be beyond the scope of the manuscript but should be at least discussed.

This is a very interesting question. We noticed that the growth arrest is very sudden upon clogger expression. At early time points, cells remain fully viable. However, at longer periods of clogger expression, cells cannot grow further even when the medium is switched back to glucose. It seems that the initial growth arrest is due to a specific response that blocks transition from G1 to S phase. The underlying mechanism might be similar to what is known for the diauxic shift when proteins shift from fermentation to respiration. However, when mitochondrial import is blocked over longer periods, the situation is more reminiscent to starvation and mitochondrial defects are likely. We will study this in more detail in the future.

11. The conclusion from figure 6G could be greatly strengthen by showing that the growth defect of wt cells could be reduced by overexpression of Hsp42/Hsp104/Hsp26. Alternatively, assuming that Hsp42/Hsp104/Hsp26 are regulated at the transcription level, their expression could be

"fixed" in wt and Δrpn4 cells by switching their promoter (pADH for example). This will render Hsp42/Hsp104/Hsp26 levels unresponsive to the presence of RPN4 and equalize the growth rates of wt and Δrpn4 cell with clogged mitochondria.
It is possible that changing the levels of these 3 proteins will not be sufficient but this will be informative too. In such case a preconditional heat shock could be used prior to clogger expression to examine whether it will help tolerate the stress.

This is an excellent suggestion! We performed this experiment as suggested and expressed Hsp42 and Hsp104 from rather strong constitutive promoters. As shown in the novel Fig. 2H, this resulted in an improved growth rate on respiratory media and an increased tolerance to clogger expression. This nicely confirms our conclusion about a protective effect of the Hsp42/Hsp104 system.
We also followed the suggestion of the referee and tested whether pre-incubation of cells at elevated temperatures provides some resistance towards clogger expression (see Figure 4 of this letter). We observed that growth at 37°C (which is not a strong heat shock for yeast cells) already provides some clogger resistance. This is consistent with previous studies from Susan Lindquist's group who showed that heat pretreatment increases the ability of cells to respire and, vice versa, growth on non-fermentable carbon sources makes cells more resistant towards heat exposure long before Hsp104-bound structures were identified (Sanchez, Y., Taulien, J., Borkovich, K. A. & Lindquist, S. (1992) Hsp104 is required for tolerance to many forms of stress, EMBO J. 11, 2357-64).

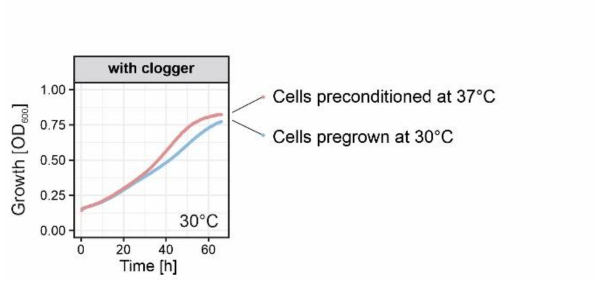

Fig. 4. Pre-conditioning at 37°C slightly increases the clogger resistance of wild type cells. Cells were grown in lactate medium at 30°C or 37°C for 24 h. Galactase was added to induce clogger expression and cell growth was monitored over time.

Minor comments:
1. Is it possible that in rpn4 del the clogger is sequestered in aggregates and is thus less accessible to clog mitochondria? Can this possibility be excluded by the MS data? It will be helpful to comment about the levels of Cyb2 in Hsp104 aggregates, in wt versus rpn4 del.

As shown in Fig. EV1A, mitochondrial precursors accumulate in the *Δrpn4* cells to even higher levels than in wild type. This excludes that *RPN4* deletion suppresses the clogger-induced import block.
We also measured comparable levels of the Cyb2 protein in both strains and show the results here for inspection of the referee in Figure 5.

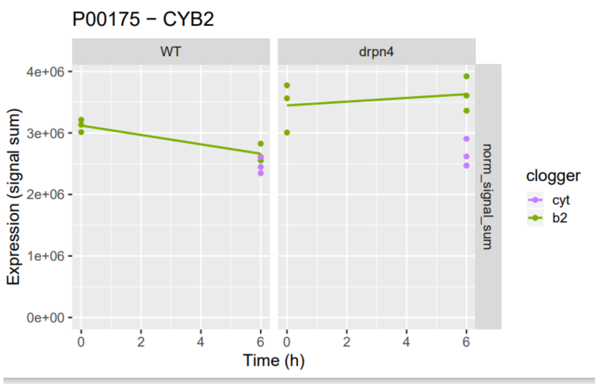

Fig. 5. The levels of the cytochrome b_2 are not considerably affect by clogger expression or the absence of Rpn4. The levels of Cyb2 were detected in the proteomics analysis described in Fig. 2B-F of our study and are here shown. Many mitochondrial proteins, including Cyb2, are reduced upon clogger expression, presumably owing to their direct competition during biogenesis. Deletion of Rpn4 prevents this depletion, again supporting our conclusion that preventing the upregulation of the proteasome in the Drpn4 cells can stabilize the mitochondrial proteome during mitochondrial dysfunction.

2. Fig 2G- what control is used here? Please add this detail.

For the control sample, a soluble cytosolic DHFR protein was expressed from a *GAL* promoter. This protein accumulates to similar levels as the cytb_2-DHFR clogger, but since it is not targeted to mitochondria it does not interfere with protein import (see Fig. 1B). We added this information now to the legend of Fig. 2 G.

3. Fig. S3A-F- luciferaseR188Q,R261Q and Ubc9Y68L are referred to as Lucts Ubc9ts in this figure. Please change to have consistent terminology.

We made the labeling now consistent.

4. "In glucose-grown wild type cells, Hsp104-GFP was evenly distributed throughout the cells whereas upon growth in non-fermenting lactate-based media cells showed a small number of aggregates (4 on average) (Fig. 3A)." Glucose conditions are not shown in the figure. Either add it to the figure or adjust the text.

We added the respective Figures to Figure 5D

5. Table 2 is not referred to in the main text. It would be helpful to add it when relevant

We refer to Table 2 now in the text.

6. Fig 6D- Hsp104-GFP is not mentioned in the figure, only in the legend. Please add.

We added this information.

7. "This precursor was protease accessible and found in high-speed pellets after solubilizing mitochondrial membranes with detergent, indicating that MitoStores remain associated with the mitochondrial surface even during cellular fractionation procedures (Fig. 6E)" Typo- should be Fig 6F.

We corrected the typo.

8. "Since Hsp42 was crucial for the formation of MitoStores (Fig. 6D) we tested whether deletion of Hsp42 suppressed the clogger resistance observed in Δrpn4 mutants (Fig. 6F)."
Typo - Fig. 6F is incorrect. Is it Fig 1F?

We corrected this.

9. Fig S5E- legend mentions 30 and 37 degrees but it seems as only one of these temperatures was used?

We corrected this.

10. Typo in the discussion session: "Hsp42 and Hsp104 are upregulated in Drpn4 cells even before clogger expression explaining the increased resistance against mitoprotein-induced stress of these cells." Drpn4 should be rpn4 deletion
We corrected this.

11. Typo Figure 6: "(F) The mitochondria used for D were treated with proteinase K (PK) to remove surface-exposed proteins." For E not for D
We corrected this.

12. Fig 6F- why is the mature band sensitive to ProK?
The total sample was not treated with Proteinase K. This is indicated by the labeling in the figure.

Reviewer #3:

We thank the reviewer for the overall positive assessment. We were pleased to read that the referee felt that 'This paper provides a step forward in our understanding of how the cytosol regulates mitochondrial precursors during stress.' and that 'The data is clear and solid.' We addressed the specific points in the following way:

Major points:
1. The authors named the Hsp104-containing granules in clogger-expressing cells or in cells cultured in non-fermentable carbon sources as "MitoStores", because these granules contain numerous mitochondrial proteins. However, this name suggests that these granules have specificity in incorporating mitochondrial proteins, but not proteins destined for the cytoplasm or other organelles. As shown in Fig. S4, proteins of the secretory pathways also interact with Hsp104 in Δrpn4 cells. Are they localized to the MitoStores as well? As the authors indicate, these structures are very similar to those identified previously as CytoQ or INQ (or JUNQ) compartments. If the authors would express a known CytoQ substrate (some they use in the paper like Ubc9 mutants or Q97-GFP, etc) in the presence of clogger, would this protein localize in the same Hsp104 puncta as mitochondrial proteins? I guess what I am getting at here is that while it's clear mitochondrial proteins can localize to Hsp104 protein aggregates when their import into mitochondria is impaired (and proteasome is inhibited), this is similar to what has been shown for other misfolded or mistargeted proteins that localize to other cellular destinations. It may not be that these aggregates are special for mitochondrial proteins, but rather, mito proteins may just represent the major class of misfolded substrates in the cell when clogger is expressed. Perhaps a good way to test the specificity of these structures is to determine whether known non-mitochondrial cytoQ substrates colocalize with mitochondrial proteins in clogger expressing cells. Things like CPY* lacking its signal sequence, or the substrates the authors use here including Ubc9 mutants or Q97-GFP. If these cytoQ substrates co-localize with mitochondrial proteins and Hsp104 when clogger is expressed, then I would recommend not using the term mitostores but rather call the structures cytoQ or just protein aggregates. Giving them the name mitostores implies that they are something different than previously described proteins aggregate structures, and this may not necessarily be the case.

In the past, cytosolic aggregates were categorized based on their intracellular localization (INQs in the nucleus, JUNQs on the nucleus, IPODs on the vacuole, or cytoQs in the cytosol) as well as by physiological aspects (stress granules, P-bodies or proteasome storage granules). These structures are not entirely distinct biochemically. For example, Hsp104 is binding to all of these types of condensates. However, they play very different roles. According to our observations, MitoStores are comparable to proteasome storage granules or PSGs which were first described in 2008 (JCB 181, 737f) and then characterized in many follow-up studies. PSGs serve as cytosolic reservoirs of proteasome subunits that form under specific growth or metabolic conditions.
We therefore prefer to use the MitoStore term which refers to the physiological relevance of this type of cytosolic granules. We fully agree with the referee that further studies in the future will have to show whether MitoStores are physically distinct from other cytoQs. We like the suggestion to co-express the clogger protein with cytosolic CPY* and to test whether both proteins form separated structures. However, we are convinced it will be important to study under more physiological conditions whether different cytosolic granules have different functions.
As a second approach, we will have to unravel whether the MitoStores described in our study are physically identical to mitochondria-associated protein aggregates that were described in the past (for example J Cell Biol 216, 2481f; Cell 159, 530f). In these previous studies, the physical association of aggregates with mitochondria was described but their protein content was not characterized so far. In will be an exciting next step to assess the potential physical association of MitoStores with the mitochondrial outer membrane and to identify outer membrane proteins which are relevant for MitoStore binding. However, we feel that this will go beyond our present study.

2. The authors showed that MTS-containing mitochondrial matrix proteins are more likely to be targeted to the MitoStores. It would be important to know whether MitoStore-targeting is highly dependent on the MTSs. For example, does removing the MTSs from MitoStore-enriched mitochondrial proteins prevent their interaction with Hsp104, and also their localizations to the MitoStores?
We followed the suggestion of the referee and tested the relevance of the MTS for MitoStore incorporation. To this end, we expressed mature versions of the MitoStore substrate Mam33 fused to RFP and tested their association with Hsp104-GFP in the presence of the clogger protein. As shown in the novel Fig. 5C, ΔMTSMam33 was equally distributed in the cytosol and did not form any aggregates. This points at a specific relevance of the mitochondrial targeting sequence for the incorporation of client proteins into MitoStores. We will elucidate the underlying molecular mechanisms in more depth in the future.

Also, for proteins like mitochondrial carriers that did not show up in the Hsp104 pull downs, it may be worthwhile to examine their colocalization with Hsp104 in clogger cells just in case their lack of identification in Hsp104 foci was due to a technical limitation because they are highly hydrophobic in nature.

Following the suggestion of the referee, we co-expressed RFP-tagged versions of the carrier proteins Dic1 and Odc1 with Hsp104-GFP. Upon clogger expression, these proteins accumulated in the cytosol but did not show colocalization with Hsp104-GFP. This confirms our observation of the non-inclusion of carriers into MitoStores. We show these data here for the inspection of the referee in Figure 6 of this letter.

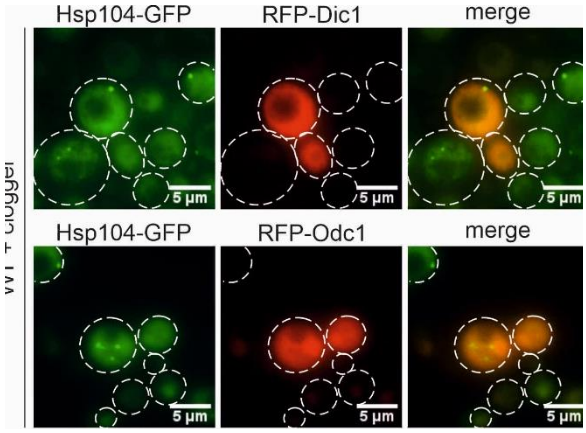

Fig. 6. Carriers are not colocalized with Hsp104-GFP. The two carrier proteins Dic1 and Odc2 were expressed as fusion proteins with N-terminal RFP domains together with Hsp104-GFP. In the presence of clogger, we saw these proteins throughout the cytosol but not accumulating in Hsp104-GFP-containing granules.

3. Through clear proteomics data, the authors showed that the MitoStore its regulated by interaction between its clients and Hsp104. They also showed used genetics and microscopy to show the formation of these granules are Hsp42-dependent. As a result, they described MitoStores as Hsp104/Hsp42-regulated. However, functional differentiation between Hsp104 and Hsp42 will be very helpful in understanding the formation of these granules. For example, whether Δhsp104 cells are able to form MitoStores, especially given that Δrpn4 Δhsp104 mutant cells showed similar growth defects as in MitoStore-deficient Δrpn4 Δhsp42 mutant cells?

We followed the suggestion of the referee to test whether Aim17-RFP is found in cytosolic granules in the absence of Hsp42 or Hsp104. No Aim17-RFP granules (MitoStores) were found in *Δhsp42* cells nor in *Δhsp104* cells. We show this now in the novel Fig. 5B. This is reminiscent to what was shown by Susan Lindquist, Bernd Bukau, Axel Mogk, Thomas Nyström and others for the Hsp42/Hsp104-dependent formation of cytoQ condensates (e.g. PMID: 15845535; 25079602; 25681695; 31649258).

Additionally, can the authors comment on why loss of both Rpn4 and Hsp42 or 104 does not lead to more toxicity in WT cells?

Clogger expression kills about 75% of all wild type and *Δrpn4 Δhsp104* cells within 24 hours. The latter mutant cannot upregulate the proteasome and does not form MitoStores but the essential parts of the chaperone system are not affected. As shown in the novel Fig. S5F, mutants lacking Rpn4, Hsp42 or Hsp104 show no generally increased heat shock response, suggesting that other chaperones such as the very potent Hsp70 and Hsp90 chaperones of the cytosol (Ssa1/2, Hsp82) are fully sufficient to maintain proteostasis under non-challenged conditions.

Minor points that should be addressed:

1. In Fig. 3C, the lower right Mass Spectrometry result, label of x-axis, right bracket after DHFR-GFP is missing.

We corrected this.

2. The label of y-axis of the bar graph in Fig. 6G is missing. Something like "OD600" or "Growth/Survival" will be helpful.

We added this.

3. It would be clearer if the proximity of MitoStores to mitochondria is shown in the Fig. 6H.

We changed the model accordingly and thank the referee for this suggestion.

4. Please correct the figure legend for Fig. S2. To me, it looks like the respiratory proteins are indicated in purple, not blue.

We corrected this.

5. "Drpn4" in the first paragraph of discussion should be "∆rpn4".

We corrected this.

6. It would be helpful to quantify and compare numbers/sizes of Hsp104 granules in Glucose- versus Lactate- containing media in Figure 5C.

We made additional pictures and show them in the revised version of our ms as Fig. 5D

1st Revision - Editorial Decision — 23rd Dec 2022

Thank you for submitting your revised manuscript. We have received comments from all referees (please see below) and I am happy to say that they now support publication. Therefore, I would ask you to please resolve a number of editorial issues that are listed in detail below. Please use the document that the data editors have added their comments to for any changes. If you have any further questions regarding the specific points listed below or the final manuscript version, please contact me.

Referee #1:

The authors have addressed all concerns.

Referee #2:

In their revised manuscript, Krämer et al. address the reviewers' criticisms and provide additional experiments to support their conclusions. I appreciate the extensive work and believe that it further strengthens the presented model.
All my comments were adequately addressed. Overall, the authors present exciting work revealing an important role for MitoStores in mitochondrial protein quality control and homeostasis. This study will greatly contribute to the field and should be published in EMBO Journal.

Referee #3:

The authors have addressed all of my concerns. Congratulations on an exciting study!

All editorial and formatting issues were resolved by the authors.

2nd Revision - Editorial Decision 12th Jan 2023

Thank you for submitting the final revised version of your manuscript and addressing the remaining points. I am happy to inform you that we have now formally accepted your study for publication in The EMBO Journal.

EMBO Press Author Checklist

Corresponding Author Name: Johannes M. Herrmann	
Journal Submitted to: EMBO Journal	
Manuscript Number: EMBOJ-2022-112309	

USEFUL LINKS FOR COMPLETING THIS FORM
The EMBO Journal - Author Guidelines
EMBO Reports - Author Guidelines
Molecular Systems Biology - Author Guidelines
EMBO Molecular Medicine - Author Guidelines

Reporting Checklist for Life Science Articles (updated January
This checklist is adapted from Materials Design Analysis Reporting (MDAR) Checklist for Authors. MDAR establishes a minimum set of requirements in transparent reporting in the life sciences (see Statement of Task: 10.31222/osf.io/9sm4x). Please follow the journal's guidelines in preparing your
Please note that a copy of this checklist will be published alongside your article.

Abridged guidelines for figures
1. Data
The data shown in figures should satisfy the following conditions:
- the data were obtained and processed according to the field's best practice and are presented to reflect the results of the experiments in an accurate and unbiased manner.
- ideally, figure panels should include only measurements that are directly comparable to each other and obtained with the same assay.
- plots include clearly labeled error bars for independent experiments and sample sizes. Unless justified, error bars should not be shown for technical
- if n<5, the individual data points from each experiment should be plotted. Any statistical test employed should be justified.
- Source Data should be included to report the data underlying figures according to the guidelines set out in the authorship guidelines on Data

2. Captions
Each figure caption should contain the following information, for each panel where they are relevant:
- a specification of the experimental system investigated (eg cell line, species name).
- the assay(s) and method(s) used to carry out the reported observations and measurements.
- an explicit mention of the biological and chemical entity(ies) that are being measured.
- an explicit mention of the biological and chemical entity(ies) that are altered/varied/perturbed in a controlled manner.
- the exact sample size (n) for each experimental group/condition, given as a number, not a range;
- a description of the sample collection allowing the reader to understand whether the samples represent technical or biological replicates (including how many animals, litters, cultures, etc.).
- a statement of how many times the experiment shown was independently replicated in the laboratory.
- definitions of statistical methods and measures:
  - common tests, such as t-test (please specify whether paired vs. unpaired), simple χ2 tests, Wilcoxon and Mann-Whitney tests, can be unambiguously identified by name only, but more complex techniques should be described in the methods section;
  - are tests one-sided or two-sided?
  - are there adjustments for multiple comparisons?
  - exact statistical test results, e.g., P values = x but not P values < x;
  - definition of 'center values' as median or average;
  - definition of error bars as s.d. or s.e.m.

Please complete ALL of the questions below.
Select "Not Applicable" only when the requested information is not relevant for your study.

Materials

Newly Created Materials	Information included in the manuscript?	In which section is the information available? (Reagents and Tools Table, Materials and Methods, Figures, Data Availability Section)
New materials and reagents need to be available; do any restrictions apply?	Yes	Materials and Methods

Antibodies	Information included in the manuscript?	In which section is the information available? (Reagents and Tools Table, Materials and Methods, Figures, Data Availability Section)
For **antibodies** provide the following information:<br>- Commercial antibodies: RRID (if possible) or supplier name, catalogue number and or/clone number<br>- Non-commercial: RRID or citation	Yes	Materials and Methods

DNA and RNA sequences	Information included in the manuscript?	In which section is the information available? (Reagents and Tools Table, Materials and Methods, Figures, Data Availability Section)
**Short novel DNA or RNA including primers, probes**: provide the sequences.	Yes	Materials and Methods, Table EV4

Cell materials	Information included in the manuscript?	In which section is the information available? (Reagents and Tools Table, Materials and Methods, Figures, Data Availability Section)
**Cell lines:** Provide species information, strain. Provide accession number in repository **OR** supplier name, catalog number, clone number, and/**OR** RRID.	Not Applicable	
**Primary cultures:** Provide species, strain, sex of origin, genetic modification status.	Not Applicable	
Report if the cell lines were recently **authenticated** (e.g., by STR profiling) and tested for mycoplasma contamination.	Not Applicable	

Experimental animals	Information included in the manuscript?	In which section is the information available? (Reagents and Tools Table, Materials and Methods, Figures, Data Availability Section)
**Laboratory animals or Model organisms:** Provide species, strain, sex, age, genetic modification status. Provide accession number in repository **OR** supplier name, catalog number, clone number, **OR** RRID.	Not Applicable	
**Animal observed in or captured from the field:** Provide species, sex, and age where possible.	Not Applicable	
Please detail **housing and husbandry conditions**.	Not Applicable	

Plants and microbes	Information included in the manuscript?	In which section is the information available? (Reagents and Tools Table, Materials and Methods, Figures, Data Availability Section)
**Plants:** provide species and strain, ecotype and cultivar where relevant, unique accession number if available, and source (including location for collected wild specimens).	Not Applicable	
**Microbes:** provide species and strain, unique accession number if available, and source.	Yes	Materials and Methods, Table EV3

Human research participants	Information included in the manuscript?	In which section is the information available? (Reagents and Tools Table, Materials and Methods, Figures, Data Availability Section)
If collected and within the bounds of privacy constraints report on age, sex and gender or ethnicity for all study participants.	Not Applicable	

Core facilities	Information included in the manuscript?	In which section is the information available? (Reagents and Tools Table, Materials and Methods, Figures, Data Availability Section)
If your work benefited from core facilities, was their service mentioned in the acknowledgments section?	Not Applicable	

Design

Study protocol	Information included in the manuscript?	In which section is the information available? (Reagents and Tools Table, Materials and Methods, Figures, Data Availability Section)
If study protocol has been **pre-registered, provide DOI in the manuscript**. For clinical trials, provide the trial registration number **OR** cite DOI.	Not Applicable	
Report the **clinical trial registration number** (at ClinicalTrials.gov or equivalent), where applicable.	Not Applicable	

Laboratory protocol	Information included in the manuscript?	In which section is the information available? (Reagents and Tools Table, Materials and Methods, Figures, Data Availability Section)
Provide DOI OR other citation details if **external detailed step-by-step protocols** are available.	Not Applicable	

Experimental study design and statistics	Information included in the manuscript?	In which section is the information available? (Reagents and Tools Table, Materials and Methods, Figures, Data Availability Section)
Include a statement about **sample size** estimate even if no statistical methods were used.	Not Applicable	
Were any steps taken to minimize the effects of subjective bias when allocating animals/samples to treatment (e.g. **randomization procedure**)? If yes, have they been described?	Not Applicable	
Include a statement about **blinding** even if no blinding was done.	Not Applicable	
Describe **inclusion/exclusion criteria** if samples or animals were excluded from the analysis. Were the criteria pre-established?<br><br>If sample or data points were omitted from analysis, report if this was due to attrition or intentional exclusion and provide justification.	Not Applicable	
For every figure, are **statistical tests** justified as appropriate? Do the data meet the assumptions of the tests (e.g., normal distribution)? Describe any methods used to assess it. Is there an estimate of variation within each group of data? Is the variance similar between the groups that are being statistically compared?	Yes	Materials and Methods, Paired student T tests and Limma for proteomics according to established procedures

Sample definition and in-laboratory replication	Information included in the manuscript?	In which section is the information available? (Reagents and Tools Table, Materials and Methods, Figures, Data Availability Section)
In the figure legends: state number of times the experiment was **replicated** in laboratory.	Yes	Materials and Methods, Figure Legends
In the figure legends: define whether data describe **technical or biological replicates**.	Yes	Materials and Methods, Figure Legends

Ethics

Ethics	Information included in the manuscript?	In which section is the information available? (Reagents and Tools Table, Materials and Methods, Figures, Data Availability Section)
Studies involving **human participants**: State details of **authority granting ethics approval** (IRB or equivalent committee(s), provide reference number for approval.	Not Applicable	
Studies involving **human participants**: Include a statement confirming that **informed consent** was obtained from all subjects and that the experiments conformed to the principles set out in the WMA Declaration of Helsinki and the Department of Health and Human Services Belmont Report.	Not Applicable	
Studies involving **human participants:** For publication of **patient photos**, include a statement confirming that consent to publish was obtained.	Not Applicable	
Studies involving experimental **animals**: State details of **authority granting ethics approva**l (IRB or equivalent committee(s), provide reference number for approval. Include a statement of compliance with ethical regulations.	Not Applicable	
Studies involving **specimen and field samples:** State if relevant **permits** obtained, provide details of authority approving study; if none were required, explain why.	Not Applicable	

Dual Use Research of Concern (DURC)	Information included in the manuscript?	In which section is the information available? (Reagents and Tools Table, Materials and Methods, Figures, Data Availability Section)
Could your study fall under dual use research restrictions? Please check biosecurity documents and list of **select agents and toxins** (CDC): https://www.selectagents.gov/sat/list.htm	Not Applicable	
If you used a select agent, is the security level of the lab appropriate and reported in the manuscript?	Not Applicable	
If a study is subject to dual use research of concern regulations, is the name of the **authority granting approval and reference number** for the regulatory approval provided in the manuscript?	Not Applicable	

Reporting

The MDAR framework recommends adoption of discipline-specific guidelines, established and endorsed through community initiatives. Journals have their own policy about requiring specific guidelines and recommendations to complement MDAR.

Adherence to community standards	Information included in the manuscript?	In which section is the information available? (Reagents and Tools Table, Materials and Methods, Figures, Data Availability Section)
State if relevant guidelines or checklists (e.g., **ICMJE, MIBBI, ARRIVE, PRISMA**) have been followed or provided.	Not Applicable	
For **tumor marker prognostic studies,** we recommend that you follow the **REMARK** reporting guidelines (see link list at top right). See author guidelines, under 'Reporting Guidelines'. Please confirm you have followed these guidelines.	Not Applicable	
For **phase II and III randomized controlled trials**, please refer to the **CONSORT** flow diagram (see link list at top right) and submit the CONSORT checklist (see link list at top right) with your submission. See author guidelines, under 'Reporting Guidelines'. Please confirm you have submitted this list.	Not Applicable	

Data Availability

Data availability	Information included in the manuscript?	In which section is the information available? (Reagents and Tools Table, Materials and Methods, Figures, Data Availability Section)
Have **primary datasets** been deposited according to the journal's guidelines (see 'Data Deposition' section) and the respective accession numbers provided in the Data Availability Section?	Yes	PRIDE database, See Materials and Methods
Were **human clinical and genomic datasets** deposited in a public access-controlled repository in accordance to ethical obligations to the patients and to the applicable consent agreement?	Not Applicable	
Are **computational models** that are central and integral to a study available without restrictions in a machine-readable form? Were the relevant accession numbers or links provided?	Not Applicable	
If publicly available data were reused, provide the respective **data citations in the reference list**.	Not Applicable	
