# Supplementary material for: MitoStores: chaperone‐controlled protein granules store mitochondrial precursors in the cytosol
Source: EMBO J. 2023 Jan 27;42(7):e112309. doi: 10.15252/embj.2022112309 (PMC10068336; doi:10.15252/embj.2022112309)
Supplement: Supplementary file 7 — Source Data for Expanded View [file EMBJ-42-e112309-s001.zip › SourceData_EV Figures/SourceData_Figure EV1.zip!/SourceData_Figure EV1/SourceData_Figure EV1A.pdf]

**Fig.EV1A**

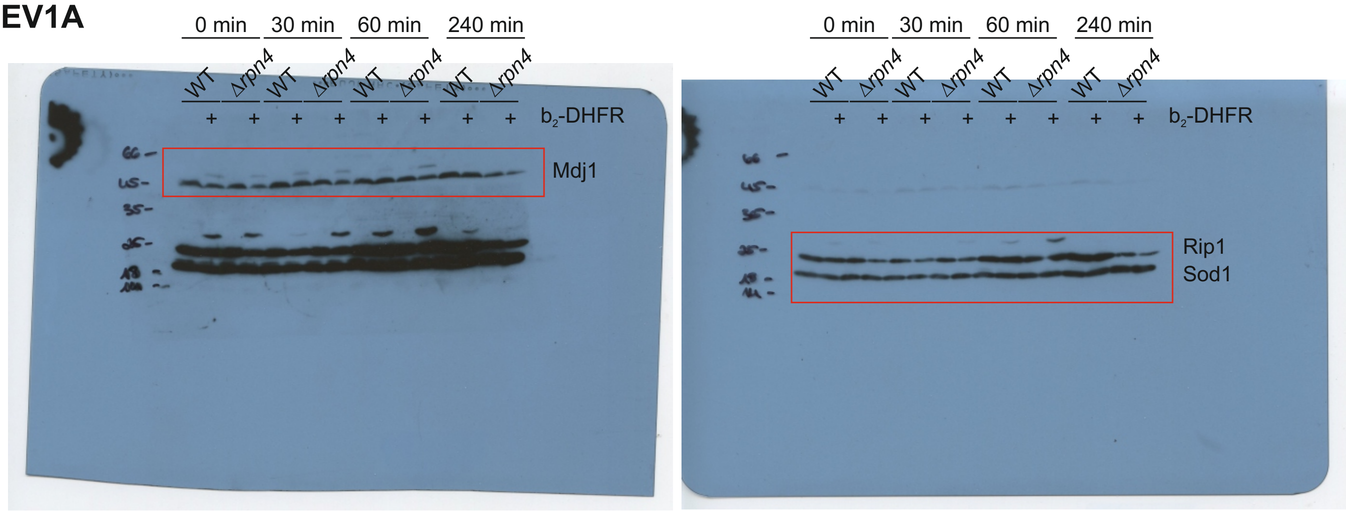
0 min
30 min
60 min
240 min
WT
Δrpn4
+
b2-DHFR
Mdj1
0 min
30 min
60 min
240 min
WT
Δrpn4
+
b2-DHFR
Rip1
Sod1
