# Supplementary material for: MitoStores: chaperone‐controlled protein granules store mitochondrial precursors in the cytosol
Source: EMBO J. 2023 Jan 27;42(7):e112309. doi: 10.15252/embj.2022112309 (PMC10068336; doi:10.15252/embj.2022112309)
Supplement: Supplementary file 7 — Source Data for Expanded View [file EMBJ-42-e112309-s001.zip › SourceData_EV Figures/SourceData_Figure EV3.zip!/SourceData_Figure EV3/SourceData_Figure EV3E.pdf]

Fig.EV3E

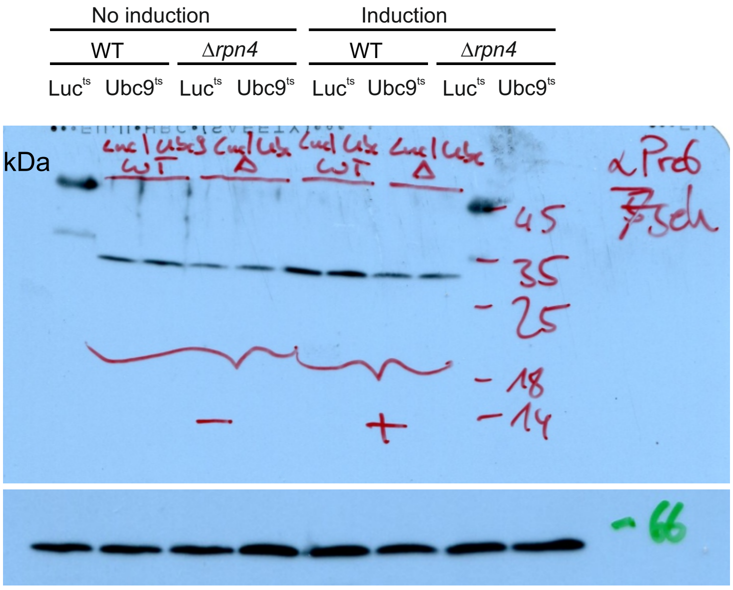

No induction
Induction
WT
Δrpn4
WT
Δrpn4
Luc^ts Ubc9^ts Luc^ts Ubc9^ts Luc^ts Ubc9^ts Luc^ts Ubc9^ts
kDa
45
35
25
18
14
66
