# Supplementary material for: MitoStores: chaperone‐controlled protein granules store mitochondrial precursors in the cytosol
Source: EMBO J. 2023 Jan 27;42(7):e112309. doi: 10.15252/embj.2022112309 (PMC10068336; doi:10.15252/embj.2022112309)
Supplement: Supplementary file 7 — Source Data for Expanded View [file EMBJ-42-e112309-s001.zip › SourceData_EV Figures/SourceData_Figure EV5.zip!/SourceData_Figure EV5/SourceData_Figure EV5C.pdf]

Fig.EV5C

Hsp104-GFP + Pdb1-RFP

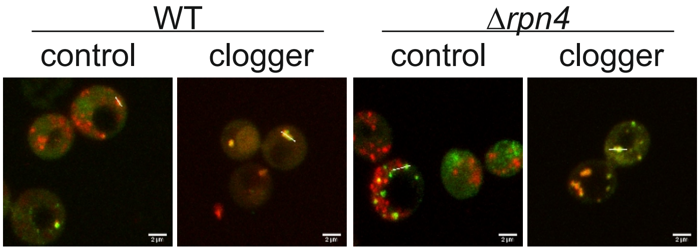

Intensities:

WT control

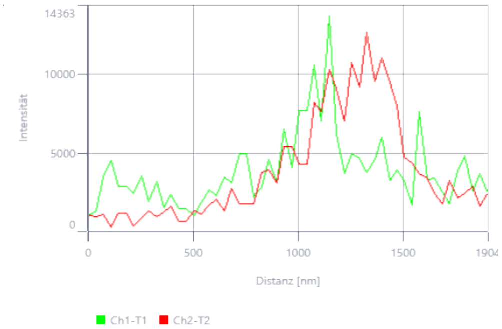

WT clogger

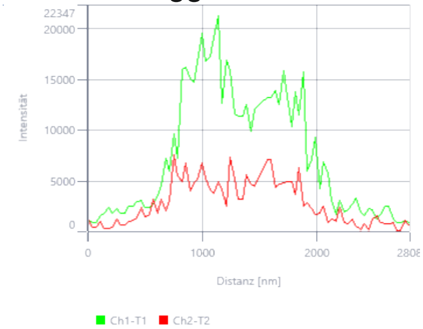

Δ*rpn4* control

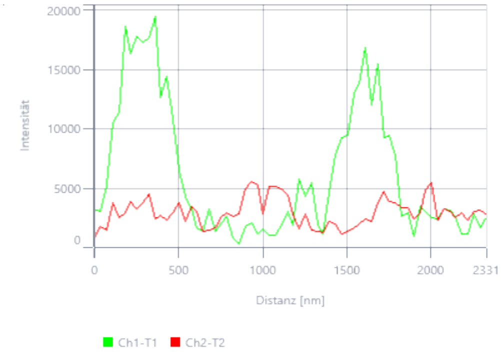

Δ*rpn4* clogger

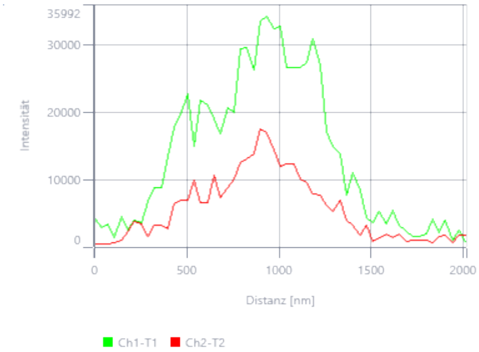

Ch1-T1= Channel 1 = GFP

Ch2-T2 = Channel 2 = RFP
